# Drug Triggers and Clinic of Acute Generalized Exanthematous Pustulosis (AGEP): A Literature Case Series of 297 Patients

**DOI:** 10.3390/jcm11020397

**Published:** 2022-01-13

**Authors:** Enriqueta Vallejo-Yagüe, Adrian Martinez-De la Torre, Omar S. Mohamad, Shweta Sabu, Andrea M. Burden

**Affiliations:** Institute of Pharmaceutical Sciences, Department of Chemistry and Applied Biosciences, ETH Zurich, CH-8093 Zurich, Switzerland; enriqueta.vallejo@pharma.ethz.ch (E.V.-Y.); adrian.martinez@pharma.ethz.ch (A.M.-D.l.T.); omohamad@student.ethz.ch (O.S.M.); shwetavenugopal.sabu@uzh.ch (S.S.)

**Keywords:** AGEP, adverse drug reaction, skin reaction, adverse event

## Abstract

Acute generalized exanthematous pustulosis (AGEP) is a rare skin reaction, commonly caused by drugs. Available evidence mostly relies on small studies or case reports. We collected published AGEP case reports and, subsequently, described the patient characteristics, suspect and concomitant drugs, time to onset, disease management, and clinical prognosis. This study included 297 AGEP patients (64.3% women) obtained from 250 published case reports or case series with individual patient data. AGEP affected patients of all ages, but the majority of patients (88.2%) were ≥25 years old. The most frequently reported suspect drugs were anti-infectives for systemic use (36.5%), particularly antibacterials for systemic use (31.0%), and especially beta-lactam antibacterials (18.3%) and macrolides (4.3%). Other frequent suspect drugs were antineoplastics (12.2%), and anti-inflammatory/anti-rheumatic products (5.2%) plus hydroxychloroquine (12.8%). Mean time to onset was 9.1 days (standard deviation SD 13.94). Some patients developed fever (64.3%) and systemic involvement (18.9%), and most patients (76.4%) received pharmacological treatment for AGEP. Seven patients died, although five of them were already critically ill prior to AGEP. In conclusion, antibiotics remain the most common suspected cause of AGEP. While case mortality rate may be up to 2.5%, disentangling the role of AGEP on the fatal outcome from the role of the preexisting health conditions remains challenging.

## 1. Introduction

Acute generalized exanthematous pustulosis (AGEP) is a rare and severe skin reaction. While AGEP occurs most frequently as an adverse effect of pharmacological treatment or drugs, it may also result from contact with chemicals (e.g., mercury) or dyes [1,2], or as a response to certain organisms (e.g., cytomegalovirus, Chlamydia pneumonia, and Escherichia coli) [3]. It has been suggested that AGEP affects one to five patients per million per year, and it has been associated with up to a 5% mortality rate, often due to multi-organ failure or secondary infections [3,4]. The key clinical feature of AGEP is the sudden presence of several miniscule, non-follicular intradermal pustules on an erythematous edematous background [5,6]. Additionally, pyrexia and pruritic or burning sensation at the erythema may also be present [7]. 

Pharmacological treatments reported as suspect agents triggering AGEP include, among others, antibiotics and other anti-infectives, cardiovascular drugs, proton pump inhibitors, non-steroidal anti-inflammatory drugs (NSAIDs), and hydroxychloroquine [7,8,9,10,11,12,13]. In drug-driven AGEP, symptoms may appear within a few hours, a few days, or even a couple of weeks after the initial drug intervention [6]. Thus, identification of the suspect drug may be challenging.

The majority of the available evidence on AGEP relies on relatively small observational studies [7,8,9,10,12,14] or individual case reports. Thus, we conducted a comprehensive narrative review of published case reports to summarize and investigate patient characteristics, suspect drugs, AGEP management, and mortality. 

## 2. Methods

### 2.1. Data Collection

A literature review in Embase and PubMed databases was conducted, aiming to collect case reports of drug-associated AGEP published before December 2021. We used keyword search terms for “acute generalized exanthematous pustulosis” or “AGEP” to identify potential articles. The study inclusion was restricted to case reports describing drug-associated AGEP in humans and providing information at the individual patient level. Only literature in English language was included. Studies of AGEP caused by insect bites, dyes, or infective agents (viruses or bacteria) were excluded. Articles describing more than one case (e.g., case series) were only included if sufficient data at the individual patient level were provided. Cases of acute localized exanthematous pustulosis (ALEP) were included and treated as AGEP. 

The articles were initially scanned through the title and abstract, and when potentially relevant, a full-text review was conducted. A database was created to record and unify data from the included case reports. Collected information comprised patient characteristics (i.e., sex, age, nationality, ethnicity, comorbidities), country of the event, every drug that the patient was taking at the onset of the reaction, the suspect drug(s) according to the original authors and their indication, record of patch test investigation, the treatment to address the AGEP reaction, and the outcome of the reaction (i.e., fever, systemic involvement, hospitalization, sequalae, death, AGEP-related death). 

The following assumptions were taken for the data collection process: If the patient’s age was reported as, for example, ‘in their 40s’, the start of the corresponding 10-year range was chosen, thus, 40 years old in this example. Ethnicity reported as ‘white’ was recorded as ‘Caucasian’. In the absence of a clear mention of the country of the event, the country of the affiliation from the main author of the corresponding paper was used. In the absence of a mention or indication of the presence of systemic involvement, surgery, or sequelae, it was assumed that there was no systemic involvement, surgery, or sequelae, respectively. If surgery after AGEP was reported, but this surgery was the consequence of pre-AGEP circumstances (e.g., trauma), it was not recorded as surgery. If AGEP was developed in already hospitalized patients, we classified them as yes for hospitalization; likewise, patients requiring hospitalization after AGEP. Performance of a percutaneous test was recorded as a patch test. Time to onset was recorded in days. In the cases when AGEP was suspected from a drug after several administrations, time to onset was considered as the number of days from first administration to the AGEP event. When time to onset was reported as ‘less than a week’ or ‘a few days’, we recorded it as six days. Similarly, time to onset of ‘a few hours’, ‘several hours’, or ‘less than 24 h’ was recorded as 0.5 days. Sudden onset was recorded as zero days, and one month as 30 days. 

### 2.2. Synthesis Methods and Reporting

Patient characteristics, number of reported drugs, number of suspect drugs, number of patients receiving pharmacological treatment to address AGEP, and the clinical outcome of the reaction were described overall and stratified by sex. Categorical variables were presented with counts and proportions, and continuous variables were described by mean and standard deviation (SD). In the stratified analysis, findings in men were compared to the findings in women using the chi-squared test for categorical variables and t-test for continuous variables. Statistical significance was defined as *p* ≤ 0.05.

Reported drugs were classified by therapeutic class, with the aid of the Anatomical Therapeutic Chemical Classification System (ATC) system and their described indication. The frequency of reporting of suspect drugs, as well as the frequency of reporting of every reported drug independently of being or not labeled as the suspect drug, were presented, providing counts and proportions, overall and stratified by sex. Subsequently, the pharmacological treatment of AGEP was similarly presented. 

The analyses were performed using R Statistical Software (R 4.0.0. R Foundation for Statistical Computing, Vienna, Austria) [15].

## 3. Results

From the >800 identified articles in PubMed and Embase, 250 published case reports or case series were included in the analysis [5,16,17,18,19,20,21,22,23,24,25,26,27,28,29,30,31,32,33,34,35,36,37,38,39,40,41,42,43,44,45,46,47,48,49,50,51,52,53,54,55,56,57,58,59,60,61,62,63,64,65,66,67,68,69,70,71,72,73,74,75,76,77,78,79,80,81,82,83,84,85,86,87,88,89,90,91,92,93,94,95,96,97,98,99,100,101,102,103,104,105,106,107,108,109,110,111,112,113,114,115,116,117,118,119,120,121,122,123,124,125,126,127,128,129,130,131,132,133,134,135,136,137,138,139,140,141,142,143,144,145,146,147,148,149,150,151,152,153,154,155,156,157,158,159,160,161,162,163,164,165,166,167,168,169,170,171,172,173,174,175,176,177,178,179,180,181,182,183,184,185,186,187,188,189,190,191,192,193,194,195,196,197,198,199,200,201,202,203,204,205,206,207,208,209,210,211,212,213,214,215,216,217,218,219,220,221,222,223,224,225,226,227,228,229,230,231,232,233,234,235,236,237,238,239,240,241,242,243,244,245,246,247,248,249,250,251,252,253,254,255,256,257,258,259,260,261,262,263,264], resulting in 297 patients included in the extracted database. 

A comprehensive database with the collected information from every patient, together with a table describing every included variable, are provided in Supplementary Material S2 Excel File.

Among the 297 included patients, 102 (34.3%) patients were men, 191 (64.3%) patients were women, and 4 patients missed information on sex. These patients’ characteristics are described in Table 1. Overall, patients’ age ranged from 1 to 93 years old, with a mean of 48.9 years (SD 20.92). The majority (88.2%) of the population were 25 years or older, with the highest overall percentage observed in the group of patients between 40 and 64 years (41.1%). Overall, 91.2% of the included articles missed a description of the ethnicity of the patient. Approximately one-quarter of patients (23.2%) had an infection at AGEP onset. Other reported observed comorbidities included cardiac/cardiovascular event/disease (21.9%), rheumatic/musculoskeletal diseases or immune-mediated disease (16.8%), skin disease/manifestations (13.8%), and cancer (11.4%) (Supplementary Material S1 Table S1). Among the overall study cases, 135 (45.5%) patients reported taking only one drug, 104 (35.0%) two or three drugs, and 58 (19.5%) four or more drugs. However, in most of the included AGEP cases (90.9%), only one drug was reported as a suspect trigger of the skin reaction, and only approximately one-third of the cases had a patch or percutaneous test recorded. Half of the cases had a time to onset ≤4 days, and the overall mean time was 9.1 days (SD 13.9) (Supplementary Material S1 Figure S1). Characterizing the AGEP event, fever and systemic involvement were reported in 191 (64.3%) and 56 (18.9%) patients, respectively. Most patients (n = 227, 76.4%) received pharmacological treatment, while 70 (23.6%) did not require or did not report pharmacological intervention. The most commonly used treatments for AGEP were corticosteroids for systemic (31.3%) and topical use (22.4%), and other dermatologicals (26.3%) (Supplementary Material S1 Table S2). Sequalae, including long-lasting skin manifestations, such as scarring and hyperpigmentation, were described in 13 (4.4%) patients. No records of surgery due to AGEP were observed. 

Stratifying by sex (Table 1), women’s mean age (51.1 years [SD 18.6]) was significantly higher than men’s mean age (45.5 years [SD 23.2]; *p* = 0.026). Across the sex strata, non-significant differences were found for the number of all and suspect reported drugs. However, a tendency of a higher number of drugs may be observed in male (24.5% with >4 drugs) versus female patients (16.8% with >4 drugs). Similarly, although there were no significant differences across the sex strata with regard to the percentage of patients developing fever, systemic involvement, or having sequalae due to AGEP, a tendency of more men (23.5%) than women (16.8%) having systemic involvement may be observed. In the men and women cohorts, 4 (3.9%) and 3 (1.6%) deaths were registered, respectively. 

In the study cohort, seven deaths were recorded [75,91,104,132,151,182,221]. A summary description of the patients with a fatal outcome is included in Table 2. Among these seven patients (four men, three women), five were critically ill before AGEP diagnosis (e.g., cancer, extent burns, pneumonia, viral infection). The most common death reasons were sepsis and multiorgan failure, and the time from AGEP varied from 20 days to 1 year since AGEP diagnosis. In two cases, AGEP had not been previously recovered.

Overall, among the 297 patients, a total of 154 unique drugs were identified as suspect triggers of AGEP. The frequency of reporting of these suspect drugs is presented in Table 3, and in more detail in the Supplementary Material S1 Table S3. Five substances were not included in these frequency tables due to the lack of an ATC code (i.e., curcumin, neurotropin, probiotics, traditional Chinese medicine, and dai-kenchu-to herbal medicine). Among the suspect drugs, the most commonly reported were anti-infectives for systemic use (36.5%), and particularly antibacterials for systemic use (31.0%), especially beta-lactam antibacterials (18.3%) and macrolides (4.3%). Antivirals for systemic use and vaccines were identified as the suspect cause in eight (2.3%) and nine (2.6%) occasions, respectively. Antineoplastic agents and immunomodulating agents represented 14.5% of the suspected drugs, with the majority of this category including antineoplastics (12.2%). Anti-inflammatory and anti-rheumatic products represented 5.2% of the suspect drugs, and this figure increased when considering hydroxychloroquine as an anti-rheumatic treatment or conventional synthetic disease-modifying anti-rheumatic drug (csDMARD), despite its classification as an anti-malarial drug following the ATC system. Indications for hydroxychloroquine included lupus, rheumatoid arthritis, other rheumatic and immune-mediating diseases, and COVID-19. Hydroxychloroquine was reported as an AGEP trigger in 44 (12.8%) patients. Nervous system treatments represented 8.1% of the suspect drug, and other less frequently reported drugs included dermatologic treatments, cardiovascular and cardiac treatments, and drugs for the alimentary tract, respiratory system, genito-urinary system, and hormonal treatments. 

Stratifying by sex, visual assessment of the findings shows a higher frequency of hydroxychloroquine and anti-inflammatory and anti-rheumatic products as suspect drugs in women than men. Conversely, a higher frequency of analgesics may be observed in the men cohort. 

A description of all drugs reported in patients developing AGEP, independently of the suspect label, is given in Table 4, and in more detail in the Supplementary Material S1 Table S4. Overall, antibacterials for systemic use were the most frequent drugs (25.7%), and other drugs with a high presence were blood/cardiovascular treatments (13.9%), antineoplastic agents (6.3%), anti-inflammatory and anti-rheumatic drugs (3.9%), hydroxychloroquine (6.6%), analgesics (4.5%), corticosteroids for systemic use (4.3%), and contrast media (5.7%).

## 4. Discussion

This literature case series included 297 patients with drug-associated AGEP, whose information was obtained from 250 published scientific articles. AGEP affected both women (64.3%) and men (34.3%), and while patients’ age covered a wide spectrum, a higher frequency was observed in patients 25 years or older (88.2%). Approximately half (50.5%) of the patients reported the use of more than one drug at the time of AGEP onset, but in 90.9% of cases, only one drug was reported as the suspect agent. Among the suspect drugs, the most frequently reported ones were antibacterials for systemic use (31.0%), particularly beta-lactam antibacterials (18.3%) and macrolides (4.3%). Other frequently reported suspect drugs were antineoplastic agents (12.2%), anti-inflammatory and anti-rheumatic products (5.2%), and hydroxychloroquine (12.8%). Time to onset was ≤4 days in 50% of the patients. Fever was reported in more than half of the cases (64.3%), and systemic involvement in one in every six patients (18.9%). Three-fourths of the patients received pharmacological treatment to address the AGEP reaction. Overall, seven case reports reported the death of the patient (four men, three women), six of which were seriously ill prior to AGEP. It remains unclear whether AGEP was the cause of every fatal outcome. 

The patient demographics in this case series are generally comparable to other observational studies. Indeed, the higher frequency of women patients in our study is in line with the existing evidence [7,11,265,266], and there is agreement on the lower age observed among men in comparison to women patients [266]. However, we note that the overall mean age in our case series (48.6 years [SD 21.6]) was slightly lower than the 51.7–62 years mean age reported in other observational and multi-center studies [7,8,10,12], as well as the 57.3 years reported in a recent pharmacovigilance study including 2649 case reports from the World Health Organization pharmacovigilance database (WHO-VigiBase) [266]. 

Aging is associated with increased frailty, higher disease burden, more medication use, and higher risk for adverse drug reactions [267]. Thus, since the majority of the study patients (67%) were older than 40, and more than the half of the study case reports included the use of two or more drugs, it remains unclear if the observed higher frequency of AGEP with increased age is due to higher frailty with aging, or due to a higher polypharmacy and its associated increased risk of interaction or additive drug effects.

Systemic involvement in AGEP patients has been described as abnormal hepatic and liver function [9,12], as well as acute respiratory distress [265]. Our observed percentage of AGEP patients developing fever (64.3%) was slightly higher than the previously reported frequency of 52% [10,11], but our estimation of the frequency of systemic involvement in AGEP patients (18.9%) lies within the previous reported range, which was 13.9% in a study of 43 cases using electronic medical records in Singapore [12], 17.2% in a French study with 58 cases [265], and 23.5% in study in Taiwan with 51 cases [9]. 

In the literature, the AGEP case-fatality rate has been suggested to be 2–5%, which is often due to multi-organ failure or secondary infections [3,4,12,13]. The 7 out of 297 patients in this case series whose death was reported and collected [75,91,104,132,151,182,221] would yield a similar case-fatality rate of 2.4%. However, disentangling the cause of death in these patients is challenging. Most of the fatal cases (five of seven) had compromised health at the time of AGEP onset (i.e., two patients had cancer [151,221], one was critically burned [104], and two had respiratory infections [75,182]). Moreover, five out the seven patients died after AGEP was deemed resolved. Only one case report clearly stated the drug reaction as the cause of death [132]. Thus, the role of AGEP in the mortality risk of these patients is questionable. Nevertheless, we note that five out of seven patients died from multiorgan failure and/or sepsis, which suggests a common path towards the fatal outcome. Additionally, death occurred within the first four months after AGEP but for one patient, who continued treatment with the suspect drug and had what we may called ‘mild chronic AGEP’ for one year [221]. While the relatively short time to outcome may support the hypothesis of AGEP influencing the mortality risk, we should consider a potential publication bias. Case reports often describe the events and circumstances after the study event but within a limited timeframe. As such, deaths occurring shortly after AGEP are more likely to be included in published case reports. Consequently, we believe the suggested 2.4% case-fatality rate should be taken with caution as both over- and under-estimation is possible.

Our findings confirmed previous observational studies indicating the high frequency of antibiotics as drugs clinically associated with AGEP onset, especially beta-lactam antibacterials. Antibiotics are often the most prevalent drugs associated with AGEP onset [7,8,9,10,11,12,13,14,265,268,269], accounting for 17 out of the 26 (61.4%) AGEP cases studied by Barbaud et al. [8], 26 of the 58 (44.8%) AGEP cases studied by Hotz et al. [265], and 14 (66.7%) out of the 21 AGEP patients studied by Choon et al. [11]. Additionally, the beta-lactam antibacterial amoxicillin was the most reported drug (21.6%) in the study of 2649 AGEP individual case safety reports (ICSRs) with at least two reported drugs in the World Health Organization (WHO) pharmacovigilance database (WHO VigiBase) [266]. In the multinational EuroSCAR case-control study, which included 97 validated AGEP cases, antibiotics, such as pristinamycin, aminopenicillins, and quinolones, were suspect drugs in 10%, 19%, and 9% of the AGEP patients, respectively. Moreover, these agents were identified as being highly associated with AGEP [7]. In our review, the most common cause of AGEP was antinfectives, including antibiotics, antimycotics, antivirals, and vaccines. Almost one-third of the AGEP reports identified antibacterials as the suspected trigger of the skin reaction, and among those, beta-lactam antibacterials were identified in more than half of the events, followed by macrolides. 

Following antibacterials and other anti-infective agents, the anti-inflammatory and anti-rheumatic products including hydroxychloroquine were also frequent among the AGEP triggers. Hydroxychloroquine was identified as the suspect drug in one of every eight patients (12.8%). This result was higher than the 7% previously observed by the EuroSCAR case-control study [7]. Since our data from 22 out of the 44 patients with hydroxychloroquine as the suspect drug were obtained from 15 articles from 2019 onwards, it might suggest a potential overestimation of the frequency of this drug in comparison to other treatments, maybe due to over-reporting as a consequence of the elevated attention given to this drug during the COVID-19 pandemic [270]. 

Anti-cancer drugs were also identified as a leading cause of AGEP in our case series (8.1% of cases). In a study investigating the adverse effects associated with targeted and non-targeted chemotherapy, imatinib was identified as one of the common anti-cancer drugs responsible for AGEP [271]. Our study was in line with these findings.

While the medications most frequently associated with AGEP events do not always share a common pharmacological pathway, many reflect a patient profile with an “active” immune response (e.g., infection, inflammation, or autoimmune disease), or abnormal cell regulation (cancer). This could also be observed across the patient comorbidities and/or health burdens prior to AGEP. This may suggest that the patient condition for which the treatment is provided may have an impact on the development of AGEP. However, the presence of other drugs also commonly associated with this reaction, such as those for cardiovascular disease, or treatments for neurological diseases (e.g., antiepileptics), does not necessarily support this hypothesis. 

In our secondary analysis where we investigated all reported drugs at the moment of AGEP onset, independently of being considered the suspect drug or not, we confirmed the strong presence of antibacterials among the patients. Interestingly, the frequency of blood/cardiovascular treatments in the all-drugs analysis was two to four times higher than in the analysis where only the suspect drugs were included, and 21.9% of patients had pre-existing cardiac/cardiovascular disease/event. While this could indicate an underlying disease–drug interaction, whereby patients with cardiac/cardiovascular diseases are at a higher susceptibility to experience AGEP, it could also suggest the potential for currently unknown drug–drug interactions or additive effects in patients with polypharmacy. A previous study identified the potential for drug interactions to play a role in the onset of Stevens–Johnson syndrome [272]. Similarly, it has been suggested that the high frequency of cardiovascular drugs observed in a data-driven pharmacovigilance study may suggest potential off-target drug–drug interactions [266]. Conversely, an alternative hypothesis could be the consequence of cardiovascular drugs being less commonly known as AGEP triggers, and therefore being less frequently identified as the suspect drug.

### Strengths and Limitations

This study is an extensive compilation of published case reports, and it provides a detailed overview of both the drug triggers of AGEP and the clinical characteristics of the reaction. Additionally, we provide a summary table and reference to the included articles (Supplementary Material S2 Excel File), enabling other researchers to investigate additional queries in this case series. However, we acknowledge some limitations. First, while we completed a comprehensive literature review, we may have missed case-reports indexed elsewhere or published in a language other than English. This study’s focus was on extracting case reports with detailed patient information and while case-series were eligible, larger studies (e.g., by Hotz et al. [265]) may have been missed in the search. For example, the study by Hotz. et al. [265] included 58 patients from a single center and was not identified as a case-report and therefore it was missed in our analysis. We did not include the patients described in the multi-center study by Barbaud et al. [8] due to limited information on the individual patient level (26 patients with provided sex, age, and drug(s) with a positive patch test). While the inclusion of the patients from these case-series papers would increase the overall size of the database, we do not expect it to have shifted the overall interpretation as our findings are comparable to those from Hotz et al. and Barbaud et al. Second, there are intrinsic limitations of literature case series. This study design does not allow for assessment of disease incidence or prevalence at the population level, we are heavily reliant on the accuracy of the initial case-report data, and we may be subject to some selection bias (publication bias) due to only including those cases with published reports. Thus, the external validity of the findings should be taken with caution. However, it is expected that the informative character of the study findings would aid to better understand the clinic of AGEP. Following the similarities between AGEP and ALEP, we included both as AGEP; however, we recorded information on the extent of skin involvement. This information is available in the Supplementary Material S2 Excel File. The assessment of certain information was additionally a challenge due to the lack of uniformity across study reports. To address this limitation, we established internal rules for data collection (described in the methods section) to ensure consistency along the process. We noticed that the number of all drugs may be underestimated, since there were case reports in which one would have expected more medications than the reported ones in patients with comorbidities. Similarly, while we have trusted the judgement of the authors of each corresponding case report and accepted their selected suspect drug, we acknowledge the potential misclassification of the suspect drugs. We did not collect information on the performance of the EuroSCAR score, and only less than half of the included cases reported the performance of the patch test for identification of the responsible drug. Moreover, given that the pathogenesis and pathomechanism of AGEP are not well understood, it may be that the drugs most frequently reported in the literature may have in a subsequently higher likelihood of being identified as causative agents. Hence, we acknowledge that the causative agent could have been one of the other concomitant drugs not considered as the suspect drug. Thus, to address this limitation, we provided a secondary analysis including every reported drug, independently of the suspect label. Additionally, since 50.5% of patients were treated with more than one drug, studying the impact of drug combinations with the collected data would be of interest. 

## 5. Conclusions

This comprehensive overview of published case reports provides a large case series of drug-associated AGEP, thereby permitting further understanding of the patient characteristics and drugs associated with this rare, and potentially fatal, adverse drug reaction. Due to the difficulties of studying rare adverse drug reactions, we expect that our study strongly contributes to the current evidence on AGEP. Among the 297 studied patients (mean age of 48.8 years old; women 64.3%), more than half developed fever, and almost 20% had systemic involvement. The majority received pharmacological treatment to address the skin reaction. Antibacterial drugs were the most commonly reported drug class, both overall and as the identified suspect agent. Seven patients died, but the role of AGEP in the cause of death may be difficult to disentangle from the previous health condition of the corresponding patients, and the publication bias. Finally, we provide a comprehensive database with the 297 patients from the included published case reports, thereby providing the largest publicly available case-series. In particular, the generated dataset may be used by others to describe AGEP cases and address additional questions related to polypharmacy, drug–drug interactions, and drug–disease interactions. Nevertheless, additional observational data is required to further elucidate the case-fatality rates and longitudinal outcomes within the population.

## Figures and Tables

**Table 1:** Characteristics of the included patients with acute generalized exanthematous pustulosis (AGEP).

	Overall	Men	Women	
Number of Patients:	(n = 297)	(n = 102)	(n = 191)	*p*-Value
Age, years (mean (SD))	48.9 (20.9)	45.5 (23.2)	51.1 (18.6)	0.026
Age group, years				0.001
<12	17 (5.7)	12 (11.8)	3 (1.6)	
12–17	9 (3.0)	5 (4.9)	3 (1.6)	
18–24	9 (3.0)	5 (4.9)	4 (2.1)	
25–39	64 (21.5)	17 (16.7)	47 (24.6)	
40–64	122 (41.1)	38 (37.3)	84 (44.0)	
≥65	76 (25.6)	25 (24.5)	50 (26.2)	
Ethnicity (%)				0.781
African American	3 (1.0)	1 (1.0)	2 (1.0)	
Asian	2 (0.7)	0 (0.0)	2 (1.0)	
Caucasian	18 (6.1)	5 (4.9)	13 (6.8)	
Hispanic	1 (0.3)	0 (0.0)	1 (0.5)	
Indo-Asian	1 (0.3)	0 (0.0)	1 (0.5)	
Latin-American	1 (0.3)	0 (0.0)	1 (0.5)	
Unknown	271 (91.2)	96 (94.1)	171 (89.5)	
Country of AGEP event				0.086
Africa	13 (4.4)	0 (0.0)	13 (6.8)	
Americas	62 (20.9)	22 (21.6)	37 (19.4)	
Asia	90 (30.3)	35 (34.3)	55 (28.8)	
Europe	124 (41.8)	44 (43.1)	79 (41.4)	
Oceania	7 (2.4)	1 (1.0)	6 (3.1)	
Unknown	1 (0.3)	0 (0.0)	1 (0.5)	
Number of all reported drugs (mean (SD))	2.4 (2.1)	2.6 (2.3)	2.3 (2.0)	0.277
Number of all reported drugs				0.431
1	135 (45.5)	44 (43.1)	90 (47.1)	
2	69 (23.2)	23 (22.5)	45 (23.6)	
3	35 (11.8)	10 (9.8)	24 (12.6)	
>4	58 (19.5)	25 (24.5)	32 (16.8)	
Number of suspect drugs (mean (SD))	1.2 (0.8)	1.3 (1.0)	1.1 (0.7)	0.261
Number of suspect drugs				0.311
1	270 (90.9)	89 (87.3)	179 (93.7)	
2	18 (6.1)	9 (8.8)	8 (4.2)	
3	5 (1.7)	2 (2.0)	2 (1.0)	
>4	4 (1.3)	2 (2.0)	2 (1.0)	
Patch test performed	103 (34.7)	36 (35.3)	66 (34.6)	0.583
missing/unknown	2 (0.7)	0 (0.0)	2 (1.0)	
AGEP time to onset, days (mean (SD))	9.1 (13.9)	8.5 (12.5)	9.5 (14.8)	0.56
AGEP characteristics				
Fever yes	191 (64.3)	70 (68.6)	119 (62.3)	0.456
missing/unknown	62 (20.9)	20 (19.6)	40 (20.9)	
Systemic involvement	56 (18.9)	24 (23.5)	32 (16.8)	0.212
Hospitalization	169 (56.9)	68 (66.7)	99 (51.8)	0.025
missing/unknown	118 (39.7)	33 (32.4)	83 (43.5)	
Pharmacological treatment of AGEP	227 (76.4)	81 (79.4)	142 (74.3)	0.488
missing/unknown	32 (10.8)	11 (10.8)	21 (11.0)	
Skin sequalae/scarring/hyperpigmentation	13 (4.4)	4 (3.9)	9 (4.7)	0.988
Death	7 (2.4)	4 (3.9)	3 (1.6)	0.393

Results as counts and percentage of the total number of patients in the corresponding group, unless otherwise specified. Findings in men were compared to those in women using the chi-squared test for categorical variables and *t*-test for continuous variables. For these tests, missing values were dropped. The overall category includes four patients with unknown sex; thus, four patients were not classified in the women or men categories. Abbreviations: SD standard deviation; AGEP Acute Generalized Exanthematous Pustulosis.

**Table 2:** Characteristics of the patients with reported death after acute generalized exanthematous pustulosis (AGEP).

Reference	Sex; Age	Baseline Comorbidities	Suspect Drug; Days to AGEP Onset	AGEP Treatment; Recovery	Death Reason; Days after AGEP
Shih et al. 2006 [221]	man 71	lung cancer and brain metastasis	gefitinib; 10 days	PT; continued gefitinib with few pustules	pneumonia with sepsis; 1 year
Liang et al. 2011 [151]	woman59	hepatocellular carcinoma; hepatitis C; ascitis; peptic ulcer	sorafenib; 14 days	no-PT; SDW; recovered/ New episode after rechallenge; SDW; recovered	pneumonia with septic shock; 2 months
Hagiya et al. 2014 [104]	man 77	critically burn (ca. 80% BSA)	daptomycin; 3 days	PT; SDW; recovered	multiple organ failure and sepsis; 20 days
Ozturk et al. 2014 [182]	man 39	ventilator associated pneumonia (VAP)	tigecycline; 2 days	PT; SDW; recovered	multiple organ failure and sepsis; 3 months (reported “unrelated to AGEP”)
Krishna et al. 2014 [132]	woman 78	metabolic syndrome; presumed pneumonia	levofloxacin; vancomycin; 12 days	PT; SDW; not-recovered	“multisystem organ failure caused by complications of AGEP without internal sources of sepsis” 4–6 days
Gambini et al. 2020 [91]	woman 80	blepharitis	betamethasone (possible cross-reaction with prior dexamethasone); 1 days	PT; recovered	“unexpected death by acute myocardial infarction”; 4 months
Delaleu et al. 2020 [75]	man 76	diabetes; COVID-19, with acute respiratory distress syndrome	hydroxychloroquine; 9 days	SDW; recovered	pulmonary embolism; 10 days

Abbreviations: PT pharmacological treatment; SDW suspect drug withdrawal; BSA body surface area.

**Table 3:** Information on the reported suspect drugs for the 297 identified patients. Patients may contribute more than one drug each.

	Overall	Men	Women
Drugs Reported as Suspect Drugs	(n = 344)	(n = 127)	(n = 210)
**ALIMENTARY TRACT AND METABOLISM**	**6 (1.7)**	**1 (0.8)**	**5 (2.4)**
Digestives, incl. enzymes	1 (0.3)	0 (0)	1 (0.5)
Drugs for acid related disorders	1 (0.3)	1 (0.8)	0 (0)
Drugs used in diabetes	2 (0.6)	0 (0)	2 (0.9)
Stomatological preparations	1 (0.3)	0 (0)	1 (0.5)
Vitamins	1 (0.3)	0 (0)	1 (0.5)
**ANTIINFECTIVES FOR SYSTEMIC USE**	**126 (36.5)**	**55 (43.3)**	**66 (31.3)**
Antibacterials for systemic use	107 (31.0)	45 (35.4)	60 (28.4)
*tetracyclines*	2 (0.6)	1 (0.8)	1 (0.5)
*beta-lactam antibacterials, penicillins*	39 (11.3)	14 (11)	25 (11.8)
*other beta-lactam antibacterials*	24 (7)	10 (7.9)	13 (6.2)
*sulfonamides and trimethoprim*	8 (2.3)	6 (4.7)	2 (0.9)
*macrolides, lincosamides and streptogramins*	15 (4.3)	6 (4.7)	9 (4.3)
*quinolone antibacterials, fluoroquinolones*	5 (1.4)	3 (2.4)	2 (0.9)
*other antibacterials*	14 (4.1)	5 (3.9)	8 (3.8)
Antimycobacterials	1 (0.3)	0 (0)	1 (0.5)
Antimycotics for systemic use	1 (0.3)	1 (0.8)	0 (0)
Antivirals for systemic use	8 (2.3)	7 (5.5)	1 (0.5)
Vaccines	9 (2.6)	2 (1.6)	4 (1.9)
*bacterial vaccines*	2 (0.6)	0 (0)	0 (0)
*viral vaccines*	7 (2)	2 (1.6)	4 (1.9)
**ANTINEOPLASTIC AND IMMUNOMODULATING AGENTS**	**50 (14.5)**	**18 (14.2)**	**33 (15.6)**
Antineoplastic agents	42 (12.2)	15 (11.8)	28 (13.3)
Endocrine therapy	1 (0.3)	1 (0.8)	0 (0)
Immunostimulants	1 (0.3)	1 (0.8)	0 (0)
Immunosuppressants	6 (1.7)	1 (0.8)	5 (2.4)
**ANTIPARASITIC PRODUCTS, INSECTICIDES AND REPELLENTS**	50 (14.5)	12 (9.4)	38 (18)
Anthelmintics	1 (0.3)	1 (0.8)	0 (0)
Antiprotozoals	48 (13.9)	10 (7.9)	38 (18)
hydroxychloroquine *	44 (12.8)	7 (5.5)	37 (17.5)
*other*	4 (1.2)	3 (2.4)	1 (0.5)
Ectoparasiticides, including scabicides, insecticides and repellents	1 (0.3)	1 (0.8)	0 (0)
**BLOOD AND BLOOD FORMING ORGANS**	**8 (2.3)**	**3 (2.4)**	**5 (2.4)**
Antianemic preparations	2 (0.6)	1 (0.8)	1 (0.5)
Antithrombotic agents	6 (1.7)	2 (1.6)	4 (1.9)
**CARDIOVASCULAR SYSTEM**	**9 (2.6)**	**2 (1.6)**	**6 (2.8)**
Agents acting on the renin-angiotensin system	1 (0.3)	0 (0)	1 (0.5)
Beta blocking agents	1 (0.3)	0 (0)	0 (0)
Calcium channel blockers	4 (1.2)	2 (1.6)	2 (0.9)
Cardiac therapy	1 (0.3)	0 (0)	1 (0.5)
Diuretics	1 (0.3)	0 (0)	1 (0.5)
Lipid modifying agents	1 (0.3)	0 (0)	1 (0.5)
**DERMATOLOGICALS**	**10 (2.9)**	**3 (2.4)**	**7 (3.3)**
Antifungals for dermatological use	10 (2.9)	3 (2.4)	7 (3.3)
**GENITO URINARY SYSTEM AND SEX HORMONES**	**3 (0.9)**	**1 (0.8)**	**2 (0.9)**
Other gynecologicals	1 (0.3)	0 (0)	1 (0.5)
Sex hormones and modulators of the genital system	1 (0.3)	0 (0)	1 (0.5)
Urologicals	1 (0.3)	1 (0.8)	0 (0)
**MUSCULO-SKELETAL SYSTEM**	**20 (5.8)**	**5 (3.9)**	**15 (7.1)**
Antigout preparations	1 (0.3)	1 (0.8)	0 (0)
Anti-inflammatory and antirheumatic products	18 (5.2)	4 (3.1)	14 (6.6)
Muscle relaxants	1 (0.3)	0 (0)	1 (0.5)
**NERVOUS SYSTEM**	**28 (8.1)**	**15 (11.8)**	**13 (6.2)**
Analgesics	10 (2.9)	6 (4.7)	4 (1.9)
Anesthetics	2 (0.6)	0 (0)	2 (0.9)
Antiepileptics	6 (1.7)	3 (2.4)	3 (1.4)
Other nervous system drugs	2 (0.6)	1 (0.8)	1 (0.5)
Psychoanaleptics	4 (1.2)	2 (1.6)	2 (0.9)
Psycholeptics	4 (1.2)	3 (2.4)	1 (0.5)
**RESPIRATORY SYSTEM**	**7 (2)**	**2 (1.6)**	**5 (2.4)**
Antihistamines for systemic use	3 (0.9)	1 (0.8)	2 (0.9)
Cough and cold preparations	3 (0.9)	1 (0.8)	2 (0.9)
Nasal preparations	1 (0.3)	0 (0)	1 (0.5)
**SENSORY ORGANS**	**1 (0.3)**	**0 (0)**	**1 (0.5)**
Ophthalmologicals	1 (0.3)	0 (0)	1 (0.5)
**SYSTEMIC HORMONAL PREPARATIONS, EXCL. SEX HORMONES AND INSULINS**	**3 (0.9)**	**1 (0.8)**	**2 (0.9)**
Corticosteroids for systemic use	3 (0.9)	1 (0.8)	2 (0.9)
**VARIOUS**	**38 (11)**	**13 (10.2)**	**24 (11.4)**
Contrast media	38 (11)	13 (10.2)	24 (11.4)

Results as counts and percentage of the total number of suspect drugs in the corresponding group. The following substances, reported in one case report each, were not included due to missing ATC code: curcumin, neurotropin, probiotics, traditional Chinese medicine, and dai-kenchu-to herbal medicine. * Note that hydroxychloroquine was classified as antimalarial following the ATC system; however, it was indicated for lupus, rheumatoid arthritis, other rheumatic and immune-mediating diseases, and COVID-19.

**Table 4:** All reported drugs, independently of being suspect or not. Information from 297 patients, who can contribute more than one drug each.

	Overall	Men	Women
All Reported Drugs, Independently of Suspect or Not	(n = 716)	(n = 265)	(n = 441)
**ALIMENTARY TRACT AND METABOLISM**	**49 (6.8)**	**13 (4.9)**	**36 (8.2)**
Antiemetics and antinauseants	3 (0.4)	1 (0.4)	2 (0.5)
Bile and liver therapy	1 (0.1)	0 (0)	1 (0.2)
Digestives, including enzymes	1 (0.1)	0 (0)	1 (0.2)
Drugs for acid-related disorders	16 (2.2)	6 (2.3)	10 (2.3)
Drugs for functional gastrointestinal disorders	3 (0.4)	2 (0.8)	1 (0.2)
Drugs used in diabetes	13 (1.8)	3 (1.1)	10 (2.3)
Mineral supplements	3 (0.4)	0 (0)	3 (0.7)
Other alimentary tract and metabolism products	2 (0.3)	0 (0)	2 (0.5)
Stomatological preparations	1 (0.1)	0 (0)	1 (0.2)
Vitamins	6 (0.8)	1 (0.4)	5 (1.1)
**ANTIINFECTIVES FOR SYSTEMIC USE**	**184 (25.7)**	**76 (28.7)**	**103 (23.3)**
Antibacterials for systemic use	154 (21.5)	62 (23.4)	90 (20.4)
Antimycobacterials	1 (0.1)	0 (0)	1 (0.2)
Antimycotics for systemic use	4 (0.6)	2 (0.8)	2 (0.5)
Antivirals for systemic use	16 (2.2)	10 (3.8)	6 (1.4)
Vaccines	9 (1.3)	2 (0.8)	4 (0.9)
**ANTINEOPLASTIC AND IMMUNOMODULATING AGENTS**	**69 (9.6)**	**31 (11.7)**	**38 (8.6)**
Antineoplastic agents	45 (6.3)	22 (8.3)	23 (5.2)
Endocrine therapy	4 (0.6)	4 (1.5)	0 (0)
Immunostimulants	3 (0.4)	1 (0.4)	2 (0.5)
Immunosuppressants	17 (2.4)	4 (1.5)	13 (2.9)
**ANTIPARASITIC PRODUCTS, INSECTICIDES AND REPELLENTS**	**55 (7.7)**	**12 (4.5)**	**43 (9.7)**
Anthelmintics	1 (0.1)	1 (0.4)	0 (0)
Antiprotozoals	53 (7.4)	10 (3.8)	43 (9.7)
hydroxychloroquine *	47 (6.6)	7 (2.6)	40 (9)
*other*	6 (0.8)	3 (1.1)	3 (0.7)
Ectoparasiticides, including scabicides, insecticides, and repellents	1 (0.1)	1 (0.4)	0 (0)
**BLOOD AND BLOOD FORMING ORGANS**	**34 (4.7)**	**17 (6.4)**	**17 (3.8)**
Antianemic preparations	5 (0.7)	2 (0.8)	3 (0.7)
Antithrombotic agents	28 (3.9)	14 (5.3)	14 (3.2)
Blood substitutes and perfusion solutions	1 (0.1)	1 (0.4)	0 (0)
**CARDIOVASCULAR SYSTEM**	**66 (9.2)**	**22 (8.3)**	**41 (9.3)**
Agents acting on the renin-angiotensin system	11 (1.5)	3 (1.1)	8 (1.8)
Beta blocking agents	8 (1.1)	2 (0.8)	5 (1.1)
Calcium channel blockers	10 (1.4)	4 (1.5)	6 (1.4)
Cardiac therapy	10 (1.4)	5 (1.9)	5 (1.1)
Diuretics	15 (2.1)	5 (1.9)	9 (2)
Lipid-modifying agents	11 (1.5)	3 (1.1)	7 (1.6)
Peripheral vasodilators	1 (0.1)	0 (0)	1 (0.2)
**DERMATOLOGICALS**	**21 (2.9)**	**9 (3.4)**	**12 (2.7)**
Antibiotics and chemotherapeutics for dermatological use	2 (0.3)	0 (0)	2 (0.5)
Antifungals for dermatological use	11 (1.5)	4 (1.5)	7 (1.6)
Antipsoriatics	4 (0.6)	3 (1.1)	1 (0.2)
Corticosteroids, dermatological preparations	4 (0.6)	2 (0.8)	2 (0.5)
**GENITO URINARY SYSTEM AND SEX HORMONES**	**8 (1.1)**	**3 (1.1)**	**5 (1.1)**
Other gynecologicals	1 (0.1)	0 (0)	1 (0.2)
Sex hormones and modulators of the genital system	4 (0.6)	0 (0)	4 (0.9)
Urologicals	3 (0.4)	3 (1.1)	0 (0)
**MUSCULO-SKELETAL SYSTEM**	**35 (4.9)**	**9 (3.4)**	**26 (5.9)**
Antigout preparations	3 (0.4)	1 (0.4)	2 (0.5)
Anti-inflammatory and antirheumatic products	28 (3.9)	7 (2.6)	21 (4.8)
Drugs for treatment of bone diseases	1 (0.1)	0 (0)	1 (0.2)
Muscle relaxants	3 (0.4)	1 (0.4)	2 (0.5)
**NERVOUS SYSTEM**	**87 (12.1)**	**41 (15.5)**	**45 (10.2)**
Analgesics	32 (4.5)	13 (4.9)	18 (4.1)
Anesthetics	13 (1.8)	10 (3.8)	3 (0.7)
Anti-Parkinson drugs	2 (0.3)	2 (0.8)	0 (0)
Antiepileptics	14 (2)	6 (2.3)	8 (1.8)
Other nervous system drugs	3 (0.4)	2 (0.8)	1 (0.2)
Psychoanaleptics	11 (1.5)	3 (1.1)	8 (1.8)
Psycholeptics	12 (1.7)	5 (1.9)	7 (1.6)
**RESPIRATORY SYSTEM**	**23 (3.2)**	**8 (3)**	**15 (3.4)**
Antihistamines for systemic use	7 (1)	3 (1.1)	4 (0.9)
Cough and cold preparations	9 (1.3)	3 (1.1)	6 (1.4)
Drugs for obstructive airway diseases	2 (0.3)	0 (0)	2 (0.5)
Nasal preparations	5 (0.7)	2 (0.8)	3 (0.7)
**SENSORY ORGANS**	**9 (1.3)**	**0 (0)**	**9 (2)**
Ophthalmologicals	9 (1.3)	0 (0)	9 (2)
**SYSTEMIC HORMONAL PREPARATIONS, EXCL. SEX HORMONES AND INSULINS**	**35 (4.9)**	**10 (3.8)**	**25 (5.7)**
Corticosteroids for systemic use	31 (4.3)	8 (3)	23 (5.2)
Pituitary and hypothalamic hormones and analogues	1 (0.1)	1 (0.4)	0 (0)
Thyroid therapy	3 (0.4)	1 (0.4)	2 (0.5)
**VARIOUS**	**41 (5.7)**	**14 (5.3)**	**26 (5.9)**
Contrast media	41 (5.7)	14 (5.3)	26 (5.9)

Results as counts and percentage of the total number of all reported drugs in the corresponding group. The following substances, reported in one case report each, were not included due to missing ATC code: acetyl choline chloride, loxoprofen, carperitide, prophylline, curcumin, neurotropin, probiotics, traditional Chinese medicine, and dai-kenchu-to herbal medicine. * Note that hydroxychloroquine was classified as antimalarial following the ATC system; however, it was indicated for lupus, rheumatoid arthritis, other rheumatic and immune-mediating diseases, and COVID-19.

## Data Availability

The created dataset and the list of articles included in this case series is provided as Supplementary Material S2 Excel File.

## Supplementary Materials

The following are available online at https://www.mdpi.com/article/10.3390/jcm11020397/s1, Supplementary Material S1 Figure S1: Time to onset (days) histogram, Supplementary Material S1 Table S1: Patient baseline comorbidities, Supplementary Material S1 Table S2: Treatment for the Acute Generalized Exanthematous Pustulosis (AGEP) reaction, Supplementary Material S1 Table S3: Suspect drugs. Information from 297 patients, who can contribute with more than one drug each, Supplementary Material S1 Table S4: All reported drugs, independently of being or not suspect. Information from 297 patients, who can contribute with more than one drug each, Supplementary Material S2 Excel File.

## References

[1] Belhadjali H., Mandhouj S., Amri M., Zakhama A. (2008). Mercury-Induced Acute Generalized Exanthematous Pustulosis Misdiagnosed as a Drug-Related Case. Contact Dermat..

[2] Ameur K., Youssef M., Belhadjali H., Soua Y., Korbi M., Henchi M.A., Zili J. (2019). Occupational Acute Generalized Exanthematous Pustulosis Induced by Disperse Dyes in a Textile. Contact Dermat..

[3] Fernando S.L. (2012). Acute Generalised Exanthematous Pustulosis. Australas. J. Dermatol..

[4] Roujeau J. (2005). Clinical Heterogeneity of Drug Hypersensitivity. Toxicology.

[5] Bostan E., Yalcin H.B., Akdogan N., Ozdemir D.A., Karaduman A. (2020). Acute Generalized Exanthematous Pustulosis Induced by Iron Carboxymaltose Infusion: A Case Report. Dermatol. Ther..

[6] Sidoroff A., Halevy S., Bavinck J.N.B., Vaillant L., Roujeau J.C. (2001). Acute Generalized Exanthematous Pustulosis (AGEP)—A Clinical Reaction Pattern. J. Cutan. Pathol..

[7] Sidoroff A., Dunant A., Viboud C., Halevy S., Bavinck J.N.B., Naldi L., Mockenhaupt M., Fagot J.P., Roujeau J.C. (2007). Risk Factors for Acute Generalized Exanthematous Pustulosis (AGEP)—Results of a Multinational Case-Control Study (EuroSCAR). Br. J. Dermatol..

[8] Barbaud A., Collet E., Milpied B., Assier H., Staumont D., Avenel-Audran M., Grange A., Amarger S., Girardin P., Guinnepain M.-T. (2013). A Multicentre Study to Determine the Value and Safety of Drug Patch Tests for the Three Main Classes of Severe Cutaneous Adverse Drug Reactions. Br. J. Dermatol..

[9] Lee Y.-Y., Chung W.-H. (2014). Acute Generalized Exanthematous Pustulosis: A Retrospective Study of 51 Cases in Taiwan. Dermatol. Sin..

[10] Thienvibul C., Vachiramon V., Chanprapaph K. (2015). Five-Year Retrospective Review of Acute Generalized Exanthematous Pustulosis. Dermatol. Res. Pract..

[11] Choon S.E., Der Y.S., Lai N.L.J., Yu S.E.E., Yap X.L., Nalini N.M. (2018). Clinical Characteristics, Culprit Drugs and Outcome of Patients with Acute Generalised Exanthematous Pustulosis Seen in Hospital Sultanah Aminah, Johor Bahru. Med. J. Malays..

[12] Oh D.A.Q., Yeo Y.W., Choo K.J.L., Pang S.M., Oh C.C., Lee H.Y. (2021). Acute Generalized Exanthematous Pustulosis: Epidemiology, Clinical Course, and Treatment Outcomes of Patients Treated in an Asian Academic Medical Center. JAAD Int..

[13] Saissi E.-H., Beau-Salinas F., Jonville-Béra A.-P., Lorette G., Autret-Leca E. (2003). Centres Régionaux de Pharmacovigilance [Drugs associated with acute generalized exanthematic pustulosis]. Ann. Dermatol. Venereol..

[14] Zhang C., Van D.N., Hieu C., Craig T. (2019). Drug-Induced Severe Cutaneous Adverse Reactions: Determine the Cause and Prevention. Ann. Allergy Asthma Immunol..

[15] R Core Team (2020). R: A Language and Environment for Statistical Computing.

[16] Agaronov A., Makdesi C., Hall C.S. (2021). Acute Generalized Exanthematous Pustulosis Induced by Moderna COVID-19 Messenger RNA Vaccine. JAAD Case Rep..

[17] Aiempanakit K., Apinantriyo B. (2020). Clindamycin-Induced Acute Generalized Exanthematous Pustulosis: A Case Report. Medicine.

[18] Álava-Cruz C., Rojas Perez-Ezquerra P., Pelta-Fernández R., Zubeldia-Ortuño J.M., de Barrio-Fernández M. (2014). Acute Generalized Exanthematous Pustulosis Due to Benznidazole. J. Allergy Clin. Immunol. Pract..

[19] Alberto C., Konstantinou M.P., Martinage C., Casassa E., Tournier E., Bagheri H., Sibaud V., Mourey L., Mazereeuw-Hautier J., Meyer N. (2016). Enzalutamide Induced Acute Generalized Exanthematous Pustulosis. J. Dermatol. Case Rep..

[20] Alegre-Sánchez A., de Perosanz-Lobo D., Pinilla-Pagnon I., Muñoz-Zato E. (2017). Sorafenib-Induced Acute Generalized Exanthematous Pustulosis: An Increasing Association?. Actas Dermosifiliogr..

[21] Altaykan A., Boztepe G., Erkin G., Ozkaya O., Ozden E. (2004). Acute Generalized Exanthematous Pustulosis Induced by Bleomycin and Confirmed by Patch Testing. J. Dermatolog. Treat..

[22] Alzahrani M.J., Moussa M.M., Alfaraj D. (2020). Acute Generalized Exanthematous Pustulosis after COVID-19 Infection: A Case Report From Saudi Arabia. Cureus.

[23] Amaral L., Carneiro-Leão L., Cernadas J.R. (2020). Acute Generalized Exanthematous Pustulosis Due to Clavulanic Acid. J. Allergy Clin. Immunol. Pract..

[24] Arsuaga M., de Miguel R., Trigo E., Barreiro P., de la Calle F., Tarin E.J., Loli-Ausejo D., Diaz M. (2020). A Case of Acute Generalized Exanthematous Pustulosis Caused by Exposure to Atovaquone/Proguanil. J. Travel Med..

[25] Assier H., Gener G., Chosidow O., Wolkenstein P., Ingen-Housz-Oro S. (2021). Acute Generalized Exanthematous Pustulosis Induced by Enoxaparin: 2 Cases. Contact Dermat..

[26] Atak M.F., Farabi B., Akbayrak A., Kalelioğlu M.B., Rao B.K. (2021). Acute Generalized Exanthematous Pustulosis Following Treatment with Favipiravir in a Patient with COVID-19 without Hydroxychloroquine Use: Report of the First Case. J. Cosmet. Dermatol..

[27] Atasoy M., Erdem T., Sari R.A. (2003). A Case of Acute Generalized Exanthematous Pustulosis (AGEP) Possibly Induced by Iohexol. J. Dermatol..

[28] Attili S.K., Ferguson J. (2009). Varenicline-Induced Acute Generalized Exanthematous Pustulosis. Clin. Exp. Dermatol..

[29] Avram M.M., Hoang M. (2009). Case 20-2009: A 79-Year-Old Woman with a Blistering Cutaneous Eruption. N. Engl. J. Med..

[30] Azad A., Connelly N. (2006). Case of Rifampicin-Induced Acute Generalized Exanthematous Pustulosis. Intern. Med. J..

[31] Bahuguna A. (2013). Acute Generalized Exanthematous Pustulosis: A Rare Side Effect of a Common over-the-Counter Drug, Acetylsalicylic Acid. Indian Dermatol. Online J..

[32] Bailey K., Mckee D., Wismer J., Shear N. (2013). Acute Generalized Exanthematous Pustulosis Induced by Hydroxychloroquine: First Case Report in Canada and Review of the Literature. J. Cutan. Med. Surg..

[33] Basnet S., Dhital R., Tharu B. (2019). Acute Generalized Exanthematous Pustulosis: A Rare Side Effect of Clindamycin. J. Community Hosp. Intern. Med. Perspect..

[34] Bavbek S., Sözener Z.Ç., Aydin Ö., Özdemir S.K., Gül Ü., Heper A.O. (2014). First Case Report of Acute Generalized Exanthematous Pustulosis Due to Intravenous Iopromide. J. Investig. Allergol. Clin. Immunol..

[35] Beaulieu V., Fournier S., Bernard J. (2019). A Rare Case of Acute Generalized Exanthematous Pustulosis (AGEP) in a 1-Year-Old Child. J. Am. Acad. Dermatol..

[36] Belda Junior W., Ferolla A.C.J. (2005). Acute Generalized Exanthematous Pustulosis (AGEP). Case Report. Rev. Inst. Med. Trop. Sao Paulo.

[37] Beltraminelli H.S., Lerch M., Arnold A., Bircher A.J., Haeusermann P. (2005). Acute Generalized Exanthematous Pustulosis Induced by the Antifungal Terbinafine: Case Report and Review of the Literature. Br. J. Dermatol..

[38] Belz D., Persa O.D., Haese S., Hunzelmann N. (2018). Acute Generalized Exanthematous Pustulosis Caused by Ibuprofen-Diagnosis Confirmed by Patch Testing. Contact Dermat..

[39] Betto P., Germi L., Bonoldi E., Bertazzoni M. (2008). Acute Localized Exanthematous Pustulosis (ALEP) Caused by Amoxicillin–Clavulanic Acid. Int. J. Dermatol..

[40] Blanes Martínez M., Silvestre Salvador J.F., Vergara Aguilera G., Betlloch Mas I., Pascual Ramírez J.C. (2003). Acute Generalized Exanthematous Pustulosis Induced by Ranitidine Hydrochloride. Contact Dermat..

[41] Bluestein S., Morris L. (2019). Acute Generalized Exanthematous Pustulosis Mimicking Toxic Shock Syndrome. Ann. Allergy Asthma Immunol..

[42] Boccaletti V., Cortelazzi C., Fantini C., Tognetti E., Fabrizi G., Pagliarello C., Di Nuzzo S. (2015). Acute Generalized Exanthematous Pustulosis Following Paracetamol Ingestion in a Child. Pediatr. Allergy Immunol..

[43] Bordel Gómez M.T., Martín García C., Meseguer Yebra C., Zafra Cobo M.I., Cardeñoso Álvarez M.E., Sánchez Estella J. (2018). First Case Report of Acute Generalized Exanthematous Pustulosis (AGEP) Caused by Gadolinium Confirmed by Patch Testing. Contact Dermat..

[44] Bosanquet D.C., Davies W.L., May K., Harding K.G., Patel G.K. (2011). Acute Generalised Exanthematous Pustulosis Following Intravitreal Ranibizumab. Int. Wound J..

[45] Botelho L.F.F., Picosse F.R., Padilha M.H., Michalany N., Góis A., Porro A.M. (2010). Acute Generalized Exanthematous Pustulosis Induced by Cefepime: A Case Report. Case Rep. Dermatol..

[46] Bressler M.Y., Minkowitz J., Pathak N., Mekaiel A., Tamez R. (2020). Acute Generalized Exanthematous Pustulosis in an African American Male Caused by Trimethoprim-Sulfamethoxazole. Cureus.

[47] Brouard M.C., Prins C., Mach-Pascual S., Saurat J.-H. (2001). Acute Generalized Exanthematous Pustulosis Associated with STI571 in a Patient with Chronic Myeloid Leukemia. Dermatology.

[48] Byerly F.L., Nelson K.C., Granko R.P., Morrell D.S., Cairns B.A. (2005). Valdecoxib-Associated Acute Generalized Exanthematous Pustulosis. Burns.

[49] Carnio L.R., Shaw M.E.J., Schnur J., Casadesus D. (2021). Concurrent Terbinafine-Induced Acute Generalised Exanthematous Pustulosis and Hepatitis. BMJ Case Rep. CP.

[50] Caro Gutiérrez D., Gómez de la Fuente E., Feltes Ochoa R.A., López Estebaranz J.L., Salamanca Santamaría F.J. (2014). Acute Generalized Exanthematous Pustulosis Caused by Varenicline. Int. J. Dermatol..

[51] Castner N.B., Harris J.C., Motaparthi K. (2018). Cyclosporine for Corticosteroid-Refractory Acute Generalized Exanthematous Pustulosis Due to Hydroxychloroquine. Dermatol. Ther..

[52] Catho G., Ader F., Chidiac C., Ferry T. (2013). Acute Generalised Exanthematous Pustulosis Due to Pristinamycin. BMJ Case Rep..

[53] Chaabane A., Aouam K., Gassab L., Njim L., Boughattas N.A. (2010). Acute Generalized Exanthematous Pustulosis (AGEP) Induced by Cefotaxime. Fundam. Clin. Pharmacol..

[54] Chaabouni R., Bahloul E., Ennouri M., Atheymen R., Sellami K., Marrakchi S., Charfi S., Boudaya S., Amouri M., Bougacha N. (2021). Hydroxychloroquine-Induced Acute Generalized Exanthematous Pustulosis: A Series of Seven Patients and Review of the Literature. Int. J. Dermatol..

[55] Chang H.-C., Ko P.-H., Chang Y.-S., Chou P.-C. (2021). Acute Generalized Exanthematous Pustulosis Induced by Atezolizumab in a Patient with Lung Adenocarcinoma. Lung Cancer.

[56] Charfi O., Kastalli S., Sahnoun R., Lakhoua G. (2015). Hydroxychloroquine-Induced Acute Generalized Exanthematous Pustulosis with Positive Patch-Testing. Indian J. Pharmacol..

[57] Chaucer B., Stone A., Whelan D., Demanes A., Fischer J.L. (2018). AGEP from a Dog Bite Treated with Ibuprofen. Am. J. Emerg. Med..

[58] Chen C.-Y., Chiang C.-P., Chen H.-C., Gao H.-W., Wang W.-M., Hung C.-T. (2020). Coexisting Mutations in CARD14 and NUDT15 Detected in a Young Female with Azathioprine Hypersensitivity Syndrome Manifested as Acute Generalized Exanthematous Pustulosis. J. Dermatol..

[59] Chen X., Yang Y.-M., Li B., Li X.-Q., Zhou C., Jin J., Cai L., Zhang J.-Z., Mu Z.-L. (2020). Acute Generalized Exanthematous Pustulosis Induced by Mycophenolate Mofetil: A Case Complicated with Pemphigus Foliaceus. Chin. Med. J..

[60] Cherif Y., Jallouli M., Mseddi M., Turki H., Bahloul Z. (2014). Acute Generalized Exanthematous Pustulosis Induced by Piroxicam: A Case Report. Indian J. Pharmacol..

[61] Chowdhury T.A., Talib K.A., Patricia J., Nye K.D., Moosa S.A. (2021). Rare and Complicated Overlap of Stevens-Johnson Syndrome and Acute Generalized Exanthematous Pustulosis. Cureus.

[62] Chu T.W., Wang S.-H., Chi C.-C., Hsiao C.-H., Su L.-H. (2014). Coexisting Staphylococcal Scalded Skin Syndrome and Acute Generalized Exanthematous Pustulosis. Dermatol. Sin..

[63] Cleminson K., Cunningham N. (2020). Acute Generalized Exanthematous Pustulosis. CMAJ.

[64] Coleman I., Ruiz G., Brahmbhatt S., Ackerman L. (2020). Acute Generalized Exanthematous Pustulosis and Stevens-Johnson Syndrome Overlap Due to Hydroxychloroquine: A Case Report. J. Med. Case Rep..

[65] Contreras-Steyls M., Vílchez-Márquez F., Mota A., Moyano B., Herrera-Ceballos E. (2013). Acute Generalized Exanthematous Pustulosis Induced by Gliclazide: A Case Report. Int. J. Dermatol..

[66] Corbalán-Vélez R., Peón G., Ara M., Carapeto F.-J. (2000). Localized Toxic Follicular Pustuloderma. Int. J. Dermatol..

[67] Corral de la Calle M., Martín Díaz M.A., Flores C.R., Vidaurrazaga C. (2005). Acute Localized Exanthematous Pustulosis Secondary to Levofloxacin. Br. J. Dermatol..

[68] Correia B., Costa J., Egipto P., Reis P. (2021). Terbinafine-Induced Acute Generalized Exanthematous Pustulosis. J. Burn. Care Res..

[69] Couture-Lapointe C., Houle M.-C., Schreiber A. (2021). Acute Generalized Exanthematous Pustulosis Due to Nystatin Confirmed by Scratch Patch Test. Contact Dermat..

[70] Crespo J., Lainez-Nuez A., Cuevas-Bravo C., Tornero P., Mateos-Mayo A., Rojas-Pérez-Ezquerra P., Noguerado-Mellado B. (2020). Acute Generalized Exanthematous Pustulosis Due to Teicoplanin. J. Investig. Allergol. Clin. Immunol..

[71] DaCunha M., Moore S., Kaplan D. (2018). Cephalexin-Induced Acute Generalized Exanthematous Pustulosis. Dermatol. Rep..

[72] Daneshpazhooh M., Tavakolpour S., Salehi Farid A., Ebadi M., Nili A., Rashidian M., Mahmoudi H. (2021). Pustular Eruption after Biosimilar Rituximab Infusion: Report of Acute Generalized Exanthematous Pustulosis in Two Patients with Pemphigus. Int. J. Dermatol..

[73] Daye M., Oltulu P. (2021). An AGEP Case Due to COVİD-19 or Favipiravir or Enoxaparin. J. Cosmet. Dermatol..

[74] De Cruz R., Ferguson J., Wee J.S., Akhras V. (2015). Acute Localised Exanthematous Pustulosis (ALEP) Induced by Clindamycin in Pregnancy. Australas J. Dermatol..

[75] Delaleu J., Deniau B., Battistella M., de Masson A., Bensaid B., Jachiet M., Lazaridou I., Bagot M., Bouaziz J.-D., Archer G. (2020). Acute Generalized Exanthematous Pustulosis Induced by Hydroxychloroquine Prescribed for COVID-19. J. Allergy Clin. Immunol. Pract..

[76] Di Lernia V., Grenzi L., Guareschi E., Ricci C. (2009). Rapid Clearing of Acute Generalized Exanthematous Pustulosis after Administration of Ciclosporin. Clin. Exp. Dermatol..

[77] Di Maso V., Cozzi M., Gerini U., Bedina E., Olivo E., Bianco F., Signoretto D., Berlot G., Boscutti G. (2020). Coupled Plasma Filtration Adsorption for Treatment of Capillary Leak Syndrome Superimposed to Acute Generalized Exanthematous Pustolosis: A Case Report. Blood Purif..

[78] di Meo N., Stinco G., Patrone P., Trevisini S., Trevisan G. (2016). Acute Localized Exanthematous Pustulosis Caused by Flurbiprofen. Cutis.

[79] Distel C., Bollea Garlatti M.L., Torre A.C., Riganti J. (2020). Acute Generalized Exanthematous Pustulosis with Features Mimicking Toxic Epidermal Necrolysis Secondary to Amiodarone. An. Bras. Dermatol..

[80] Doğan S., Kılıç A., Artüz F., Kadan E. (2019). Case of Erythema Multiforme Associated with Pustules: Coexistence of Erythema Multiforme Minor and Acute Generalized Exanthamatous Pustulosis. Turk J. Dermatol..

[81] Duman H., Topal I.O., Kocaturk E., Cure K., Mansuroglu I. (2017). Acute Generalized Exanthematous Pustulosis Induced by Hydroxychloroquine: A Case with Atypical Clinical Presentation. An. Bras. Dermatol..

[82] Elston G.E., Johnston G.A., Mortimer N.J., Harman K.E. (2007). Acute Generalized Exanthematous Pustulosis Associated with Azathioprine Hypersensitivity. Clin. Exp. Dermatol..

[83] Enos T., Jeong H.S., Vandergriff T., Jacobe H.T., Chong B.F. (2020). Acute Generalized Exanthematous Pustulosis Induced by Empiric Hydroxychloroquine for Presumed COVID-19. Dermatol. Ther..

[84] Ersoy S., Paller A.S., Mancini A.J. (2004). Acute Generalized Exanthematous Pustulosis in Children. Arch. Dermatol..

[85] Evans C.C., Bergstresser P.R. (2004). Acute Generalized Exanthematous Pustulosis Precipitated by Hydroxychloroquine. J. Am. Acad. Dermatol..

[86] Fernando S.L., Li J., Toon C.W., Weir C. (2021). Acute Generalized Exanthematous Pustulosis to a Novel Oral Anticoagulant (Apixaban). Ann. Allergy Asthma Immunol..

[87] Fitzgerald D.A., Heagerty A.H.M., Stephens M., Smith A.G. (1994). Follicular Toxic Pustuloderma Associated with Allopurinol. Clin. Exp. Dermatol..

[88] Fu L.W., Jagdis A., Lee J.K. (2014). Acute Generalized Exanthematous Pustulosis from Imatinib. Allergy Asthma Clin. Immunol..

[89] Furukawa H., Omura R., Sugiura K., Kanazawa N., Inoue N., Qian H., Li X., Tsuruta D., Hashimoto T. (2021). Granular C3 Dermatosis-like Immunological Manifestation Found in a Case of Acute Generalized Exanthematous Pustulosis: Implication for the Mechanism in C3 Deposition to the Epidermal Basement Membrane Zone. J. Dermatol..

[90] Gaibino N., Bigotte Vieira M., Filipe P., Oliveira A. (2016). Acute Generalised Exanthematous Pustulosis Due to Amoxicillin-Clavulanate. BMJ Case Rep..

[91] Gambini D., Sena P., Raponi F., Bianchi L., Hansel K., Tramontana M., Stingeni L. (2020). Systemic Allergic Dermatitis Presenting as Acute Generalized Exanthematous Pustulosis Due to Betamethasone Sodium Phosphate. Contact Dermat..

[92] Gammoudi R., Ben Salem C., Boussofara L., Fathallah N., Ghariani N., Slim R., Sriha B., Belajouza C., Nouira R., Denguezli M. (2018). Acute Generalized Exanthematous Pustulosis Induced by Oxacillin Confirmed by Patch Testing. Contact Dermat..

[93] Gesierich A., Rose C., Brocker E.-B., Trautmann A., Leverkus M. (2006). Acute Generalised Exanthematous Pustulosis with Subepidermal Blisters of the Distal Extremities Induced by Diltiazem. Dermatology.

[94] Gey A., Milpied B., Dutriaux C., Mateus C., Robert C., Perro G., Taieb A., Ezzedine K., Jouary T. (2016). Severe Cutaneous Adverse Reaction Associated with Vemurafenib: DRESS, AGEP or Overlap Reaction?. J. Eur. Acad. Dermatol. Venereol..

[95] Ghazawi F.M., Colantonio S., Bradshaw S., Lacroix J., Pratt M. (2020). Acute Generalized Exanthematous Pustulosis Induced by Topical Morphine and Confirmed by Patch Testing. Dermatitis.

[96] Ghoshal L., Nandi S., Sarkar A., Das S. (2015). Acute Generalized Exanthematous Pustulosis Due to Meropenem: An Unusual Side Effect of a Commonly Used Drug. Indian Dermatol. Online J..

[97] Gilissen L., Huygens S., Goossens A., Breynaert C., Schrijvers R. (2020). Utility of Patch Testing for the Diagnosis of Delayed-Type Drug Hypersensitivity Reactions to Clindamycin. Contact Dermat..

[98] Goeschke B., Braathen L.R. (2004). Acute Generalized Exanthematic Pustulosis: A Case and an Overview of Side Effects Affecting the Skin Caused by Celecoxib and Other COX-2 Inhibitors Reported so Far. Dermatology.

[99] Torrijos E., Calle M., Díaz Y., Lozano L., Ortega A., Bonilla P., Alfaya T., Rodríguez R. (2017). Acute Localized Exanthematous Pustulosis Due to Bemiparin. J. Investig. Allergol. Clin. Immunol..

[100] Gonzalo-Garijo M.A., Pérez-Calderón R., De Argila D., Rodríguez-Nevado I. (2003). Metamizole-Induced Acute Generalized Exanthematous Pustulosis. Contact Dermat..

[101] Gordon J. (2016). Acute Generalized Exanthematous Pustulosis: An Uncommon Cause of Fever and Rash. Am. J. Emerg. Med..

[102] Grandvuillemin A., Ripert C., Sgro C., Collet E. (2014). Iodinated Contrast Media-Induced Acute Generalized Exanthematous Pustulosis Confirmed by Delayed Skin Tests. J. Allergy Clin. Immunol. Pract..

[103] Gualtieri B., Solimani F., Hertl M., Buhl T., Möbs C., Pfützner W. (2020). Interleukin 17 as a Therapeutic Target of Acute Generalized Exanthematous Pustulosis (AGEP). J. Allergy Clin. Immunol. Pract..

[104] Hagiya H., Kimura M., Miyamoto T., Haruki Y., Otsuka F. (2014). Acute Generalized Exanthematous Pustulosis Caused by Daptomycin in a Critically Ill Burn Victim. Intern. Med..

[105] Hammerbeck A.A., Daniels N.H., Callen J.P. (2009). Ioversol-Induced Acute Generalized Exanthematous Pustulosis: A Case Report. Arch. Dermatol..

[106] Haraszti S., Sendil S., Jensen N. (2020). Delayed Presentation of Acute Generalized Exanthematous Pustulosis Following Treatment with Cefepime in a Patient with COVID-19 without the Use of Hydroxychloroquine. Am. J. Case Rep..

[107] Harries M.J., McIntyre S.J., Kingston T.P. (2006). Co-Amoxiclav-Induced Acute Generalized Exanthematous Pustulosis Confirmed by Patch Testing. Contact Dermat..

[108] Heinzerling L.M., Pichler W., Anliker M.D. (2011). Acute Generalized Exanthematous Pustulosis Induced by Methylphenidate: A New Adverse Effect. Arch. Dermatol..

[109] Henning M.A., Opstrup M.S., Taudorf E.H. (2019). Acute Generalized Exanthematous Pustulosis to Amoxicillin. Dermatitis.

[110] Ho W.-T., Chang M.-E., Chen T.-J. (2016). An 82-Year-Old Woman Admitted for Intermittent Fever With Skin Pustulosis. Infect. Dis. Clin. Pract..

[111] Hopkins Z., Frigerio A., Clarke J.T. (2018). Acute Localized Exanthematous Pustulosis (ALEP) Caused by Lamotrigine. JAAD Case Rep..

[112] Hsieh C.-H., Yao C.-A., Lo Y. (2021). Acute Generalized Exanthematous Pustulosis Induced by Hydroxychloroquine. Kaohsiung J. Med. Sci..

[113] Huilaja L., Kallioinen M., Soronen M., Riekki R., Tasanen K. (2014). Acute Localized Exanthematous Pustulosis on Inguinal Area Secondary to Piperacillin/Tazobactam. Acta Derm. Venereol..

[114] Hung C.-J., Cheng J.-J., Lai P.-J., Lin W.-L., Hsiao Y.-P. (2015). Leucomycin-Induced Acute Generalized Exanthematous Pustulosis Complicated with Pitting Edema of the Legs—ScienceDirect. Dermatol. Sin..

[115] Hwang S.J.E., Carlos G., Wakade D., Sharma R., Fernandez-Penas P. (2016). Ipilimumab-Induced Acute Generalized Exanthematous Pustulosis in a Patient with Metastatic Melanoma. Melanoma Res..

[116] İslamoğlu Z.G.K., Karabağli P. (2019). A Case of Recalcitrant Acute Generalized Exanthematous Pustulosis with Sjogren’s Syndrome: Successfully Treated with Low-Dose Cyclosporine. Clin. Case Rep..

[117] Izquierdo J.H., Bonilla-Abadía F., Ochoa C.D., Agualimpia A., Tobón G.J., Cañas C.A. (2012). Acute Generalized Exanthematous Pustulosis Due to Tocilizumab in a Rheumatoid Arthritis Patient. Case Rep. Rheumatol..

[118] Jakhar J., Badyal R., Kumar S., Prasad S. (2021). Olanzapine-Induced Acute Generalized Exanthematous Pustulosis: A Case Report. Indian J. Psychiatry.

[119] Juan W.-H., Yang L.-C., Hong H.-S. (2004). Acute Generalized Exanthematous Pustulosis Induced by Topical Lindane. Dermatology.

[120] Kaneko S., Shimizu M., Irabu H., Yachie A. (2019). Acute Generalized Exanthematous Pustulosis in a Child with Fasciitis. Pediatr. Int..

[121] Kang S.-Y., Park S.-Y., Kim J.-H., Lee S.M., Lee S.P. (2021). COVID-19 Vaccine-Induced Acute Generalized Exanthematous Pustulosis. Korean J. Intern. Med..

[122] Kardaun S.H., de Monchy J.G. (2006). Acute Generalized Exanthematous Pustulosis Caused by Morphine, Confirmed by Positive Patch Test and Lymphocyte Transformation Test. J. Am. Acad. Dermatol..

[123] Kavala M., Zindancı I., Türkoglu Z., Can B., Kocatürk E., Senol S., Topaloglu F. (2013). Acute Generalized Exanthematous Pustulosis Induced by Etanercept: Another Dermatologic Adverse Effect. Case Rep. Dermatol. Med..

[124] Khan M., Wakelin S. (2020). Cetirizine Induced Acute Generalized Exanthematous Pustulosis Confirmed by Patch Testing. Contact Dermat..

[125] Kim H.-J., Jung K.-D., Lee K.-T., Byun J.-Y., Lee D.-Y., Lee J.-H., Yang J.-M., Lee E.-S. (2011). Acute Generalized Exanthematous Pustulosis Caused by Diltiazem. Ann. Dermatol..

[126] Kim S.-J., Lee T., Lee Y.S., Bae Y.-J., Cho Y.S., Moon H.-B., Kim T.-B. (2010). Acute Generalized Exanthematous Pustulosis Caused by Radiocontrast Media. Ann. Allergy Asthma Immunol..

[127] Kim S.W., Lee U.H., Jang S.J., Park H.S., Kang Y.S. (2010). Acute Localized Exanthematous Pustulosis Induced by Docetaxel. J. Am. Acad. Dermatol..

[128] Kline A., Fischer G. (2016). Acute Generalised Exanthematous Pustulosis and Other Severe Drug Eruptions from over the Counter Medications: A Case Report and Review of the Literature. Australas J. Dermatol..

[129] Knoell K.A., Lynch J.M. (2009). Photoinduced Acute Exanthematous Pustulosis Caused by Ciprofloxacin and Sunlight Exposure. Int. J. Dermatol..

[130] Komiya N., Takahashi K., Kato G., Kubota M., Tashiro H., Nakashima C., Nakamura T., Iwanaga K., Kimura S., Sueoka-Aragane N. (2021). Acute Generalized Exanthematous Pustulosis Caused by Erlotinib in a Patient with Lung Cancer. Case Rep. Oncol..

[131] Kostaki M., Polydorou D., Adamou E., Chasapi V., Antoniou C., Stratigos A. (2019). Acute Localized Exanthematous Pustulosis Due to Metronidazole. J. Eur. Acad. Dermatol. Venereol..

[132] Krishna S., Ortega-Loayza A., Malakouti N., Brinster N. (2014). A Rapidly Progressive and Fatal Case of Atypical Acute Generalized Exanthematous Pustulosis. J. Am. Acad. Dermatol..

[133] Kubin M.E., Jackson P., Riekki R. (2016). Acute Generalized Exanthematous Pustulosis Secondary to Acyclovir Confirmed by Positive Patch Testing. Acta Derm. Venereol..

[134] Kumar S.L., Rai R. (2011). Hydroxyzine-Induced Acute Generalized Exanthematous Pustulosis: An Uncommon Side Effect of a Common Drug. Indian J. Dermatol..

[135] Kusutani N., Nishida M., Sowa-Osako J., Maekawa N., Fukai K. (2021). Acute Generalized Exanthematous Pustulosis Induced by Pseudoephedrine in a Combination Tablet with Fexofenadine. Int. J. Dermatol..

[136] Kyriakou A., Zagalioti S.-C., Patsatsi A., Galanis N., Lazaridou E. (2019). Piperacillin/Tazobactam as Cause of Acute Generalized Exanthematous Pustulosis. Case Rep. Dermatol. Med..

[137] Ladhari C., Mokni S., Fathallah N., Zariaa S., Boussofara L., Aounallah A., Sriha B., Belajouza C., Ben Salem C., Denguezli M. (2019). An Unusual Photodistributed Acute Generalized Exanthematous Pustulosis Induced by Terbinafine. Therapie.

[138] Lakshmi C., Pillai S., Srinivas C.R. (2010). Lapatinib-Induced Acute Generalized Exanthematous Pustulosis. Indian Dermatol. Online J..

[139] Larif S., Slim R., Fathallah N., Ghariani N., Hmouda H., Ben Salem C. (2016). Lornoxicam-Induced Acute Generalized Exanthematous Pustulosis. Int. J. Dermatol..

[140] Lateo S., Boffa M.J. (2002). Localized Toxic Pustuloderma Associated with Nimesulide Therapy Confirmed by Patch Testing. Br. J. Dermatol..

[141] Leclair M.-A., Maynard B., St-Pierre C. (2009). Acute Generalized Exanthematous Pustulosis with Severe Organ Dysfunction. CMAJ.

[142] Lee E.Y., Koh M.J.A. (2021). Acute Generalized Exanthematous Pustulosis in Children and Adolescents in Singapore: A Ten-Year Retrospective Review. Pediatric Dermatol..

[143] Lee I., Turner M., Lee C.-C.R. (2010). Acute Patchy Exanthematous Pustulosis Caused by Sulfamethoxazole-Trimethoprim. J. Am. Acad. Dermatol..

[144] Lee J., Endicott A., Shinkai K. (2021). Acute Generalized Exanthematous Pustulosis. JAMA Dermatol..

[145] Lee S.K., Kim M.S., Lee U.H. (2018). Acute Generalized Exanthematous Pustulosis Induced by a Digestive Enzyme Drug, Festal^®^. Clin. Exp. Dermatol..

[146] Lee U.H., Yang J.H., Choi J.C., Chun D.K. (2004). Acute Generalized Exanthematous Pustulosis in a Six-Year-Old Boy. J. Dermatol..

[147] Leng T.Y., Aan M.K.J., Chan M., Tsien L.T. (2011). Acute Generalized Exanthematous Pustulosis Caused by Daptomycin. Ann. Dermatol..

[148] Levy Z.D., Slowey M., Schulder M. (2017). Acute Generalized Exanthematous Pustulosis Secondary to Levetiracetam and Valproic Acid Use. Am. J. Emerg. Med..

[149] Li L.-L., Lu Y.-Q., Li T. (2020). Acute Generalized Exanthematous Pustulosis with Airway Mucosa Involvement: A Case Report. WJCC.

[150] Li P.H., Wong J.C., Lau C. (2020). Importance of Allergological Evaluation and Skin Testing for Severe Cutaneous Adverse Reactions: A Case Report. Hong Kong Med. J..

[151] Liang C.-P., Yang C.-S., Shen J.-L., Chen Y.-J. (2011). Sorafenib-Induced Acute Localized Exanthematous Pustulosis in a Patient with Hepatocellular Carcinoma. Br. J. Dermatol..

[152] Liccioli G., Marrani E., Giani T., Simonini G., Barni S., Mori F. (2019). The First Pediatric Case of Acute Generalized Exanthematous Pustulosis Caused by Hydroxychloroquine. Pharmacology.

[153] Liquete E., Ali S., Kammo R., Ali M., Alali F., Challa H., Fata F. (2012). Acute Generalized Exanthematous Pustulosis Induced by Erlotinib (Tarceva) with Superimposed Staphylococcus Aureus Skin Infection in a Pancreatic Cancer Patient: A Case Report. Case Rep. Oncol..

[154] Lombardo M., Cerati M., Pazzaglia A. (2003). Acute Generalized Exanthematous Pustulosis Induced by Terbinafine. J. Am. Acad. Dermatol..

[155] Lospinoso K., Nichols C.S., Malachowski S.J., Mochel M.C., Nutan F. (2021). A Case of Severe Cutaneous Adverse Reaction Following Administration of the Janssen Ad26.COV2.S COVID-19 Vaccine. JAAD Case Rep..

[156] Machet P., Marcé D., Ziyani Y., Dumont M., Cornillier H., Jonville-Bera A.-P., Machet L. (2019). Acute Generalized Exanthematous Pustulosis Induced by Iomeprol with Cross-Reactivity to Other Iodinated Contrast Agents and Mild Reactions after Rechallenge with Iopromide and Oral Corticosteroid Premedication. Contact Dermat..

[157] Mäkelä L., Lammintausta K. (2008). Etoricoxib-Induced Acute Generalized Exanthematous Pustulosis. Acta. Derm. Venereol..

[158] Mameli C., Tadini G., Cattaneo D., Cerini C., Zuccotti G.V. (2013). Acute Generalized Exanthematous Pustulosis Induced by Paroxetine in an Adolescent Girl. Acta Derm. Venereol..

[159] Masood S., Rizwan M., Fatima S., Jalil P. (2021). Acute Generalized Exanthematous Pustulosis Induced by Cetuximab. Cureus.

[160] Masuda Y., Aoshima M., Shimauchi T., Funakoshi A., Fujiyama T., Ito T., Tokura Y. (2018). Acute Generalized Exanthematous Pustulosis Caused by Fexofenadine. J. Cutan. Immunol. Allergy.

[161] Matsubara T., Uchi H., Haratake N., Takamori S., Toyozawa R., Miura N., Yamaguchi M., Seto T., Takenoyama M. (2020). Acute Generalized Exanthematous Pustulosis Caused by the Combination of Pembrolizumab Plus Chemotherapy in a Patient With Squamous-Cell Carcinoma. Clin. Lung Cancer.

[162] Matsuda-Hirose H., Sho Y., Yamate T., Nakamura Y., Saito K., Takeo N., Nishida H., Ishii K., Sugiura K., Hatano Y. (2020). Acute Generalized Exanthematous Pustulosis Induced by Hydroxychloroquine Successfully Treated with Etretinate. J. Dermatol..

[163] Matsuo S., Nishizawa A., Oshio-Yoshii A., Satoh T. (2017). Influenza Vaccine-Induced Acute Generalized Exanthematous Pustulosis during Pregnancy. J. Dermatol..

[164] Mawri S., Jain T., Shah J., Hurst G., Swiderek J. (2015). Vancomycin-Induced Acute Generalized Exanthematous Pustulosis (AGEP) Masquerading Septic Shock-an Unusual Presentation of a Rare Disease. J. Intensiv. Care.

[165] Mercogliano C., Khan M., Lin C., Mohanty E., Zimmerman R. (2018). AGEP Overlap Induced by Hydroxychloroquine: A Case Report and Literature Review. J. Community Hosp. Intern. Med. Perspect..

[166] Mitri F., Toberer F., Enk A.H., Hartmann M. (2021). Acute Generalized Exanthematous Pustulosis in Close Temporal Association with MRNA-1273 Vaccine. Acta Derm. Venereol..

[167] Mizuta T., Kasami S., Shigehara Y., Kato M. (2020). Acute Generalized Exanthematous Pustulosis Caused by Iopamidol with Recurrence on Rechallenge with Iopromide. JAAD Case Rep..

[168] Mizuta T., Tanaka A. (2020). Tosufloxacin-Induced Acute Generalized Exanthematous Pustulosis Confirmed by a Drug-Induced Lymphocyte Stimulation Test. JAAD Case Rep..

[169] Mofarrah R., Mofarrah R., Oshriehye M., Ghobadi Aski S., Nazemi N., Nooshiravanpoor P. (2021). The Necessity of Patch Testing in Determining the Causative Drug of AGEP. J. Cosmet. Dermatol..

[170] Mohaghegh F., Jelvan M., Rajabi P. (2018). A Case of Prolonged Generalized Exanthematous Pustulosis Caused by Hydroxychloroquine-Literature Review. Clin. Case Rep..

[171] Montagnon C.M., Bridges A.G. (2020). Acute Generalized Exanthematous Pustulosis Secondary to Oral Nystatin. Mayo Clin. Proc..

[172] Moreno-Arrones O.M., Carrillo-Gijon R., Sendagorta E., Rios-Buceta L. (2018). Acute Generalized Exanthematous Pustulosis Simulating Stevens-Johnson Syndrome/Toxic Epidermal Necrolysis Associated with the Use of Vismodegib. JAAD Case Rep..

[173] Murad A., Murphy A. (2014). Cutaneous Vasculitis Overlap with Acute Generalised Exanthematous Pustulosis (AGEP). BMJ Case Rep..

[174] Murase C., Takeichi T., Sugiura K., Akiyama M. (2021). Acute Generalized Exanthematous Pustulosis Triggered by Acetaminophen in an IL36RN Variant Heterozygote. J. Dermatol..

[175] Nakamura Y., Takemoto A., Muto M. (2011). Acute Generalized Exanthematous Pustulosis Due to Etodolac in a Patient with an Iliopsoas Muscle Abscess. Acta Derm. Venereol..

[176] Nielsen R.M., Pallesen K.A. (2020). Photoinduced Acute Exanthematous Pustulosis Caused by Dicloxacillin and Exposure to Sunlight. Clin. Case Rep..

[177] O’Brian M., Kolitz E., Jeong H.S., Cao L., Vandergriff T., Glass D.A., Dominguez A.R. (2021). A Severe Presentation of Acute Generalized Exanthematous Pustulosis with Non-Infectious Circulatory Shock in an Adolescent. Pediatr. Dermatol..

[178] Ocerin-Guerra I., Gomez-Bringas C., Aspe-Unanue L., Ratón-Nieto J.A. (2012). Nystatin-Induced Acute Generalized Exanthematous Pustulosis. Actas Dermosifiliogr..

[179] Otake-Irie H., Nakajima S., Okamoto N., Toichi E., Nomura T., Kabashima K. (2020). Prolonged Acute Generalized Exanthematous Pustulosis and Atypical Target-like Lesions Induced by Hydroxychloroquine. J. Dermatol..

[180] Otsuka A., Tanizaki H., Okamoto N., Takagaki K. (2005). A Case of Acute Generalized Exanthematous Pustulosis Caused by Lincomycin. J. Dermatol..

[181] Ozkaya-Parlakay A., Azkur D., Kara A., Yildiz Y., Orhan D., Cengiz A.B., Ersoy-Evans S. (2011). Localized Acute Generalized Exanthematous Pustulosis with Amoxicillin and Clavulanic Acid. Turk J. Pediatr..

[182] Ozturk S., Ustun C., Pehlivan S., Ucak H. (2014). Acute Generalized Exanthematous Pustulosis Associated with Tigecycline. Ann. Dermatol..

[183] Ozturk U., Sungur M.A., Karakas T., Mulayim K., Ozturk P. (2015). Acute Generalized Exanthematous Pustulosis Induced by Iodixanol (Visipaque): A Serious Reaction to a Commonly Used Drug. Cutan. Ocul. Toxicol..

[184] Page B., Borradori L., Beltraminelli H., Yawalkar N., Hunger R.E. (2018). Acute Generalized Exanthematous Pustulosis Associated with Ipilimumab and Nivolumab. J. Eur. Acad. Dermatol. Venereol..

[185] Pakdeethai J., Ho S.-A., Aw D., Tan K.B. (2011). Acute Generalized Exanthematous Pustulosis-like, Folliculitic Drug Reaction Pattern Caused by Celecoxib. Dermatol. Ther..

[186] Paolino A., Walsh S., Basu T., Creamer D. (2018). Severe Drug-Induced Kidney Injury in Acute Generalized Exanthematous Pustulosis. Clin. Exp. Dermatol..

[187] Paradisi A., Bugatti L., Sisto T., Filosa G., Amerio P.L., Capizzi R. (2008). Acute Generalized Exanthematous Pustulosis Induced by Hydroxychloroquine: Three Cases and a Review of the Literature. Clinical. Ther..

[188] Park J.-J., Yun S.J., Lee J.-B., Kim S.-J., Won Y.H., Lee S.-C. (2010). A Case of Hydroxychloroquine Induced Acute Generalized Exanthematous Pustulosis Confirmed by Accidental Oral Provocation. Ann. Dermatol..

[189] Patel D., Hamarshi M., Newland M. (2018). Acute Generalized Exanthematous Pustulosis (AGEP) Secondary to Olanzapine. Crit. Care Med..

[190] Peterson A., Katzberg R.W., Fung M.A., Wootton-Gorges S.L., Dager W. (2006). Acute Generalized Exanthematous Pustulosis as a Delayed Dermatotoxic Reaction to IV-Administered Nonionic Contrast Media. Am. J. Roentgenol..

[191] Pettit C., Massick S., Bechtel M. (2018). Cannabidiol-Induced Acute Generalized Exanthematous Pustulosis. Dermatitis.

[192] Pettit C., Trinidad J., Kaffenberger B. (2020). A Case of Vancomycin-Induced Acute Generalized Exanthematous Pustulosis Confirmed by Patch Testing. J. Clin. Aesthet. Dermatol..

[193] Pita J., Fernandes R., Loureiro C., Todo Bom A. (2019). Acute Generalized Exanthematous Pustulosis. Rpia.

[194] Poliak N., Elias M., Cianferoni A., Treat J. (2010). Acute Generalized Exanthematous Pustulosis: The First Pediatric Case Caused by a Contrast Agent. Ann. Allergy Asthma Immunol..

[195] Power A.E., Graudins L.V., McLean C.A., Hopper I. (2015). Probable Fenofibrate-Induced Acute Generalized Exanthematous Pustulosis. Am. J. Health Syst. Pharm..

[196] Pretel M., Iñarrairaegui M., Lera J.M., Aguado L., Idoate M.A. (2014). Acute Generalized Exanthematous Pustulosis Induced by Sorafenib. JAMA Dermatol..

[197] Price K.N., Hendricks A.J., Goodrich M.E., Krase J.M., Shi V.Y. (2020). Widespread Pustular Eruption Following Probiotic Use. Dermatol. Online J..

[198] Prieto A., DE Barrio M., Lóupez-Sáaez P., Baeza M.L., de Benito V., Olalde S. (1997). Recurrent Localized Pustular Eruption Induced by Amoxicillin. Allergy.

[199] Punyaratabandhu P., Chirachanakul P. (2021). Cutaneous Eruption in COVID-19-Infected Patients in Thailand: An Observational Descriptive Study. J. Dermatol..

[200] Qu Y.-J., Jin S.-B., Han X.-C., Zheng L.-Q. (2016). Acute Localized Exanthematous Pustulosis Caused by Cefoperazone and Sodium Sulbactam. An. Bras. Dermatol..

[201] Rajgopal Bala H., Jalilian C., Goh M.S., Williams R., Tan G., Chong A.H. (2017). Two Cases of Amoxycillin-Induced Follicular Acute Localised Exanthematous Pustulosis. Australas. J. Dermatol..

[202] Rashid R.S., Ahmed I., Shim T.N. (2020). Acute Generalized Exanthematous Pustulosis Due to Dextromethorphan. Contact Dermat..

[203] Rastogi S., Modi M., Dhawan V. (2009). Acute Localized Exanthematous Pustulosis (ALEP) Caused by Ibuprofen A Case Report. Br. J. Oral Maxillofac. Surg..

[204] Reap L.E., Rodd C., Larios J., Marshall M. (2019). Hydrochlorothizide-Induced Acute Generalised Exanthematous Pustulosis Presenting with Bilateral Periorbital Impetigo. BMJ Case Rep..

[205] Reddy S., Bade N., Hasan S., Reddy K. (2018). Flutter, Fever, and a Fiery Red Rash: A Case of AGEP Secondary to Diltiazem. Ann. Allergy Asthma Immunol..

[206] Ridha Z., Guirguis J., Ouchene L., Chergui M., Litvinov I.V., Netchiporouk E. (2021). Acute Generalized Exanthematous Pustulosis Overlapping with Toxic Epidermal Necrolysis Successfully Treated with Etanercept. J. Eur. Acad. Dermatol. Venereol..

[207] Robustelli Test E., Vezzoli P., Carugno A., Raponi F., Gianatti A., Rongioletti F., Sena P. (2020). Acute Generalized Exanthematous Pustulosis with Erythema Multiforme-like Lesions Induced by Hydroxychloroquine in a Woman with Coronavirus Disease 2019 (COVID-19). J. Eur. Acad. Dermatol. Venereol..

[208] Rosen A., Del Paggio J.C., Chan B., Abu-Abed S., Rawls M., Ellis A.K. (2018). Acute Generalized Exanthematous Pustulosis with Multisystem Manifestations: Pinpoint Pustules and Purulent Lakes. Ann. Allergy Asthma Immunol..

[209] Ross C.L., Shevchenko A., Mollanazar N.K., Hsu S., Motaparthi K. (2018). Acute Generalized Exanthematous Pustulosis Due to Terbinafine. Dermatol. Ther..

[210] Ryder E.N.C., Perkins W. (2018). Acute Localised Exanthematous Pustulosis: Case Report, Review of the Literature and Proposed Diagnostic Criteria. Australas. J. Dermatol..

[211] Saliba E., Chrabieh R., Tannous Z. (2021). Fluconazole-Induced Acute Generalized Exanthematous Pustulosis. Am. J. Emerg. Med..

[212] Salman A., Yucelten D., Akin Cakici O., Kepenekli Kadayifci E. (2019). Acute Generalized Exanthematous Pustulosis Due to Ceftriaxone: Report of a Pediatric Case with Recurrence after Positive Patch Test. Pediatr. Dermatol..

[213] Sampson M.M., Klinkova O., Vitko J., Casanas B. (2018). A Plethora of Pustules: Acute Generalized Exanthematous Pustulosis. Am. J. Med..

[214] Sánchez-Velázquez A., Arroyo-Andrés J., Falkenhain-López D., Peralto J.L.R., Romero P.L.O., Díaz R.R., Calleja-Algarra A. (2021). Hydroxychloroquine-induced Acute Generalized Exanthematous Pustulosis: An Adverse Reaction to Keep in Mind during COVID-19 Pandemic. JDDG J. Der Dtsch. Dermatol. Ges..

[215] Sarradin V., Dalenc F., Sibaud V., Tournier E., Roché H. (2018). Acute Generalized Exanthematous Pustulosis Induced by Docetaxel and Recurrent With Letrozole: A Case Report. Clin. Breast Cancer.

[216] Schrom K., Pacifico A., Conic R.R.Z., Pigatto P.D.M., Malagoli P., Morrone A., Finelli R., Bragazzi N.L., Damiani G. (2019). Dabigatran-Associated Acute Generalized Exanthematous Pustulosis (AGEP) in a Psoriatic Patient Undergoing Ixekizumab and Its Pathogenetic Mechanism. Dermatol. Ther..

[217] Schwarz M., Kreuzer K.-A., Baskaynak G., Dörken B., Le Coutre P. (2002). Imatinib-Induced Acute Generalized Exanthematous Pustulosis (AGEP) in Two Patients with Chronic Myeloid Leukemia. Eur. J. Haematol..

[218] Scott A.D., Lee M., Kubba F., Chu A. (2015). Acute Generalized Exanthematous Pustulosis (AGEP) Secondary to Imatinib in a Patient with Chronic Myeloid Leukaemia. Clin. Exp. Dermatol..

[219] Senilă S., Seicean A., Fechete O., Grad A., Ungureanu L. (2017). Infliximab-Induced Acne and Acute Localized Exanthematous Pustulosis: Case Report. Dermatol. Ther..

[220] Shah N., Rijal A., Mishra D.R., Gallo E.S. (2020). Triad of Acute Generalized Exanthematous Pustulosis, Delirium, and Lactic Acidosis Due to Azithromycin. JAAD Case Rep..

[221] Shih H.-C., Hsiao Y.-P., Wu M.F., Yang J.-H. (2006). Gefitinib-Induced Acute Generalized Exanthematous Pustulosis in Two Patients with Advanced Non-Small-Cell Lung Cancer. Br. J. Dermatol..

[222] Shin H.-T., Park S.-W., Lee K.-T., Park H.-Y., Park J.-H., Lee D.-Y., Lee J.-H., Yang J.-M., Lee E.-S. (2011). A Case of Celecoxib Induced Acute Generalized Exanthematous Pustulosis. Ann. Dermatol..

[223] Shindo T., Masuda Y., Imai Y., Nagano T., Nishioka H. (2019). Case Report: Acute Generalized Exanthematous Pustulosis Caused by Praziquantel. Am. J. Trop. Med. Hyg..

[224] Shingade P.U., Wankhede V., Kataria P.S., Sonone N. (2014). Rare Case of Phenytoin Induced Acute Generalized Exanthematous Pustulosis with Cerebellar Syndrome. Indian J. Dermatol..

[225] Shuttleworth D. (1989). A Localized, Recurrent Pustular Eruption Following Amoxycillin Administration. Clin. Exp. Dermatol..

[226] Sim H.S., Seol J.E., Chun J.S., Seo J.K., Lee D., Sung H.S. (2011). Acute Localized Exanthematous Pustulosis on the Face. Ann. Dermatol..

[227] Skalli S., Barret P., Villier C., Bussières J.-F. (2011). Carbamazepine-Induced Acute Generalized Exanthematous Pustulosis: A Case Report. J. Pediatr. Pharmacol. Ther..

[228] Soria A., Amsler E., Bernier C., Milpied B., Tétart F., Morice C., Dezoteux F., Ferrier-Le Bouedec M.-C., Barbaud A., Staumont-Sallé D. (2021). DRESS and AGEP Reactions to Iodinated Contrast Media: A French Case Series. J. Allergy Clin. Immunol. Pract..

[229] Soria A., Barbaud A., Assier H., Avenel-Audran M., Tétart F., Raison-Peyron N., Amarger S., Girardin P., Francès C., FISARD (French Investigators for Skin Adverse Reaction to Drugs) (2015). Cutaneous Adverse Drug Reactions with Antimalarials and Allergological Skin Tests. Dermatology.

[230] Spadaro A., Gartland R., Conlon L.W. (2021). Images in Emergency Medicine: Acute Generalized Exanthematous Pustulosis. J. Emerg. Med..

[231] Stingeni L., Francisci D., Bianchi L., Hansel K., Tramontana M., Di Candilo F., Mannarino M.R., Pirro M. (2021). Severe Adverse Drug Reaction in SARS-CoV-2 Infection: AGEP Induced by Ceftriaxone and Confirmed by Patch Test. Contact Dermat..

[232] Suh H.Y., Bae J., Kim H.L., Kim K.H., Cha R.-H., Ahn J.Y., Youn J.I., Park M.Y. (2018). A Case of Acute Generalized Exanthematous Pustulosis after Injection of an Erythropoiesis-Stimulating Agent. Ann. Dermatol..

[233] Syed T., Abdullah A.S., Mubasher M., Yousaf Z., Mohamed M.F.H., Alweis R. (2021). Acute Generalized Exanthematous Pustulosis with Multiple Organ Failure. Case Rep. Dermatol..

[234] Tajmir-Riahi A., Wörl P., Harrer T., Schliep S., Schuler G., Simon M. (2017). Life-Threatening Atypical Case of Acute Generalized Exanthematous Pustulosis. Int. Arch. Allergy Immunol..

[235] Tak H., Koçak C., Sarıcı G., Dizen Namdar N., Kıdır M. (2015). An Uncommon Side Effect of Bupropion: A Case of Acute Generalized Exanthematous Pustulosis. Case Rep. Dermatol. Med..

[236] Tan C.M., Zipursky J.S. (2020). Acute Generalized Exanthematous Pustulosis Caused by an Intravenous Radiocontrast Medium. CMAJ.

[237] Tedbirt B., Viart-Commin M., Carvalho P., Courville P., Tétart F. (2021). Severe Acute Generalized Exanthematous Pustulosis (AGEP) Induced by Miconazole Oral Gel with Overlapping Features of Drug Reaction with Eosinophilia and Systemic Symptoms (DRESS). Contact Dermat..

[238] Thédenat B., Loche F., Albes B., Marguery M.C., Bazex J. (2001). Acute Generalized Exanthematous Pustulosis with Photodistribution Pattern Induced by Sertraline. Dermatology.

[239] Thomas J., Durack A., Flanagan N., Meligonis G. (2015). Residents’corner October 2015. DeRmpath&Clinic: Acute Generalised Exanthematous Pustulosis. Eur. J. Dermatol..

[240] Torres-Navarro I., Abril-Pérez C., Roca-Ginés J., Sánchez-Arráez J., Botella-Estrada R. (2020). A Case of Cefditoren-Induced Acute Generalized Exanthematous Pustulosis during COVID-19 Pandemics. Severe Cutaneous Adverse Reactions Are an Issue. J. Eur. Acad. Dermatol. Venereol..

[241] Toyoshima H., Mizuno M., Tanigawa M., Tanaka H., Nakanishi Y., Sakabe S. (2021). Acute Generalized Exanthematous Pustulosis in a Postpartum Woman. Clin. Case Rep..

[242] Tresch S., Cozzio A., Kamarashev J., Harr T., Schmid-Grendelmeier P., French L.E., Feldmeyer L. (2012). T Cell–Mediated Acute Localized Exanthematous Pustulosis Caused by Finasteride. J. Allergy Clin. Immunol..

[243] Treudler R., Grunewald S., Gebhardt C., Simon J.-C. (2009). Prolonged Course of Acute Generalized Exanthematous Pustulosis with Liver Involvement Due to Sensitization to Amoxicillin and Paracetamol. Acta Derm. Venereol..

[244] Tsutsumi R., Yoshida Y., Adachi K., Nanba E., Yamamoto O. (2018). Acute Localized Exanthematous Pustulosis Caused by a Herbal Medicine, Dai-Kenchu-To. Contact Dermat..

[245] Turrentine J.E., Dharamsi J.W., Miedler J.D., Pandya A.G. (2011). Acute Generalized Exanthematous Pustulosis (AGEP) Caused by Telavancin. J. Am. Acad. Dermatol..

[246] Umayahara T., Shimauchi T., Fujiyama T., Ito T., Hirakawa S., Tokura Y. (2013). Paediatric Acute Generalized Exanthematous Pustulosis Induced by Paracetamol with High Serum Levels of Interleukin-8 and -22: A Case Report. Acta Derm. Venereol..

[247] Velter C., Schissler C., Moulinas C., Tebacher-Alt M., Siedel J.-M., Cribier B., Lipsker D. (2017). Acute Generalized Exanthematous Pustulosis Caused by an Iodinated Contrast Radiocontrast Medium for Computed Tomography Arthrography of the Knee. Contact Dermat..

[248] Vickers J.L., Matherne R.J., Mainous E.G., Kelly B.C. (2008). Acute Localized Exanthematous Pustulosis: A Cutaneous Drug Reaction in a Dental Setting. J. Am. Dent. Assoc..

[249] Vigarios E., Tournier E., Pouessel D., Cohen-Jonathan-Moyal E., Sibaud V. (2017). Oral Lesions of Acute Generalized Exanthematous Pustulosis. Int. J. Dermatol..

[250] Villani A., Baldo A., De Fata Salvatores G., Desiato V., Ayala F., Donadio C. (2017). Acute Localized Exanthematous Pustulosis (ALEP): Review of Literature with Report of Case Caused by Amoxicillin-Clavulanic Acid. Dermatol. Ther..

[251] Wakelin S.H., James M.P. (1995). Diltiazem-Induced Acute Generalised Exanthematous Pustulosis. Clin. Exp. Dermatol..

[252] Wang G., Zhuo N., Li J. (2021). Acute Generalized Exanthematous Pustulosis Induced by Generic Hydroxychloroquine. J. Cosmet. Dermatol..

[253] Won J.H., Yun S.J., Kim S.J., Lee S.C., Won Y.H., Lee J.B. (2009). A Case of Acute Generalized Exanthematous Pustulosis Possibly Induced by Ritodrine. Ann. Dermatol..

[254] Wu C.-S., Chang W.-Y., Lan C.-C.E., Chen G.-S., Chiu H.-H. (2008). Acute Generalized Exanthematous Pustulosis Possibly Induced by Acarbose. Int. J. Dermatol..

[255] Wu M.-Y., Yen H., Wang C.-W., Chung W.-H. (2020). Curcumin-Induced Acute Generalized Exanthematous Pustulosis. Indian J. Dermatol. Venereol. Leprol..

[256] Wu R.-W., Lin T.-K. (2021). Oxford-AstraZeneca COVID-19 Vaccine-Induced Acute Localized Exanthematous Pustulosis. J. Dermatol..

[257] Yalçın B., Çakmak S., Yıldırım B. (2015). Successful Treatment of Hydroxychloroquine-Induced Recalcitrant Acute Generalized Exanthematous Pustulosis with Cyclosporine: Case Report and Literature Review. Ann. Dermatol..

[258] Yamamoto Y., Kadota M., Nishimura Y. (2004). A Case of Eperisone Hydrochloride-Induced Acute Generalized Exanthematous Pustulosis. J. Dermatol..

[259] Yamazaki Y., Watanabe R., Ishikawa T., Munetsugu T., Nishizawa A., Satoh T. (2018). Acute Generalized Exanthematous Pustulosis Associated with Helicobacter Pylori Eradication Therapy with Elevated Serum Procalcitonin. Eur. J. Dermatol..

[260] Yesudian P.D., Penny M., Azurdia R.M., King C.M. (2004). Ibuprofen-Induced Acute Generalized Exanthematous Pustulosis. Int. J. Dermatol..

[261] Yonit W., Ilan G., Idit S., Sarah B. (2004). A Case of Paracetamol-Induced Acute Generalized Exanthematous Pustulosis in a Pregnant Woman Localized in the Neck Region. SKINmed Dermatol. Clin..

[262] Zhang L., Qian M., Hong L., Wu Q. (2020). First Case Report of Acute Generalized Exanthematous Pustulosis (AGEP) Caused by Mifepristone. Contact Dermat..

[263] Zhang Z., Liu X. (2015). Acute Generalized Exanthematous Pustulosis. N. Engl. J. Med..

[264] Zheng J., Gao Y., Yi X., Ding Y. (2018). A Case of Ceftriaxone-Induced Acute Generalized Exanthematous Pustulosis/Generalized Pustular Psoriasis Overlap. Case Rep. Dermatol..

[265] Hotz C., Valeyrie-Allanore L., Haddad C., Bouvresse S., Ortonne N., Duong T.A., Ingen-Housz-Oro S., Roujeau J.C., Wolkenstein P., Chosidow O. (2013). Systemic Involvement of Acute Generalized Exanthematous Pustulosis: A Retrospective Study on 58 Patients. Br. J. Dermatol..

[266] Martinez-De la Torre A., van Weenen E., Kraus M., Weiler S., Feuerriegel S., Burden A.M. (2021). A Network Analysis of Drug Combinations Associated with Acute Generalized Exanthematous Pustulosis (AGEP). J. Clin. Med..

[267] Alhawassi T.M., Krass I., Bajorek B.V., Pont L.G. (2014). A Systematic Review of the Prevalence and Risk Factors for Adverse Drug Reactions in the Elderly in the Acute Care Setting. Clin. Interv. Aging.

[268] Lee H.Y., Chou D., Pang S.M., Thirumoorthy T. (2010). Acute Generalized Exanthematous Pustulosis: Analysis of Cases Managed in a Tertiary Hospital in Singapore. Int. J. Dermatol..

[269] Roujeau J.-C., Bioulac-Sage P., Bourseau C., Guillaume J.-C., Bernard P., Lok C., Plantin P., Claudy A., Delavierre C., Vaillant L. (1991). Acute Generalized Exanthematous Pustulosis: Analysis of 63 Cases. Arch. Dermatol..

[270] Prats-Uribe A., Sena A.G., Lai L.Y.H., Ahmed W.-U.-R., Alghoul H., Alser O., Alshammari T.M., Areia C., Carter W., Casajust P. (2021). Use of Repurposed and Adjuvant Drugs in Hospital Patients with COVID-19: Multinational Network Cohort Study. BMJ.

[271] Baldo B.A., Pham N.H. (2013). Adverse Reactions to Targeted and Non-Targeted Chemotherapeutic Drugs with Emphasis on Hypersensitivity Responses and the Invasive Metastatic Switch. Cancer Metastasis Rev..

[272] Cheng F.-J., Syu F.-K., Lee K.-H., Chen F.-C., Wu C.-H., Chen C.-C. (2016). Correlation between Drug-Drug Interaction-Induced Stevens-Johnson Syndrome and Related Deaths in Taiwan. J. Food Drug Anal..

