# Supplementary material for: Drug Triggers and Clinic of Acute Generalized Exanthematous Pustulosis (AGEP): A Literature Case Series of 297 Patients
Source: J Clin Med. 2022 Jan 13;11(2):397. doi: 10.3390/jcm11020397 (PMC8780223; doi:10.3390/jcm11020397)
Supplement: Supplementary file 1 [file jcm-11-00397-s001.zip › jcm-1550740 Supplementary/jcm-1550740-Supplementary Material S1.pdf]

Journal of
Clinical Medicine

MDPI

Supplementary Material S1

Drug Triggers and Clinic of Acute Generalized Exanthematous Pustulosis (AGEP): A Literature Case Series of 297 Patients

Enriqueta Vallejo-Yagüe †, Adrian Martinez-De la Torre †, Omar Mohamad, Shweta Sabu and Andrea M. Burden *

Department of Chemistry and Applied Biosciences, Institute of Pharmaceutical Sciences, ETH Zurich, 8092 Zurich, Switzerland; enriqueta.vallejo@pharma.ethz.ch (E.V.-Y.); adrian.martinez@pharma.ethz.ch (A.M.-D.l.T.); omohamad@student.ethz.ch (O.M.); shsabu@student.ethz.ch (S.S.)

* Correspondence: andrea.burden@pharma.ethz.ch; Tel.: +41-76-685-22-56

† These authors contributed equally to this work.

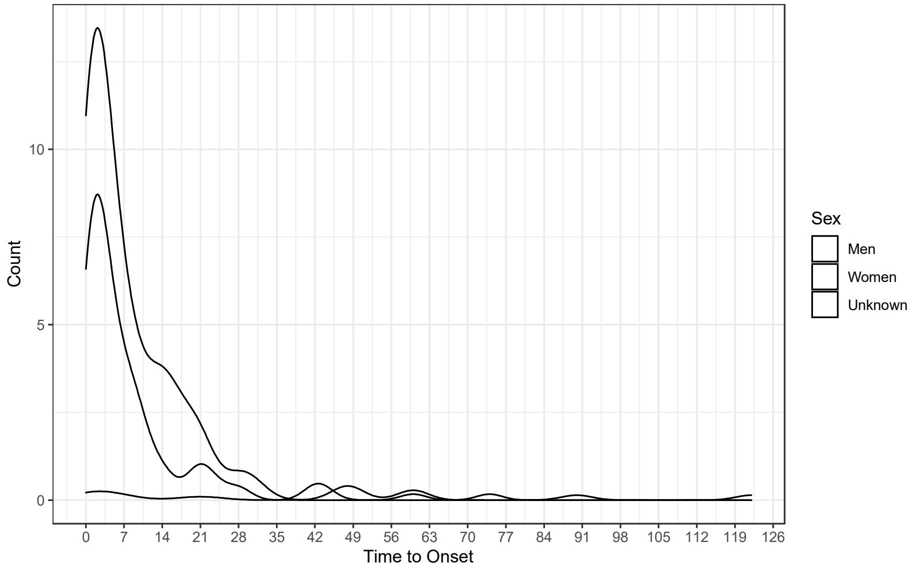

Figure S1. Time to onset (days) histogram.

Table S1. Patient baseline comorbidities.

	Overall (n=297)	Men (n=102)	Women (n=191)
**Infection**	**69 (23.2)**	**32 (31.4)**	**37 (19.4)**
infection	42 (14.1)	20 (19.6)	22 (11.5)
infection respiratory	15 (5.1)	5 (4.9)	10 (5.2)
infection oropharyngeal	7 (2.4)	4 (3.9)	3 (1.6)
infection, sepsis	2 (0.7)	2 (2)	0 (0)
infection parasite	2 (0.7)	1 (1)	1 (0.5)
infection, respiratory	1 (0.3)	0 (0)	1 (0.5)
**Cardiac/cardiovascular event/disease**	**65 (21.9)**	**23 (22.5)**	**42 (22)**
hypertension	29 (9.8)	8 (7.8)	21 (11)
cardiac event/disease	22 (7.4)	10 (9.8)	12 (6.3)
dyslipidemia	4 (1.3)	2 (2)	2 (1)
hyperlipidemia	3 (1)	1 (1)	2 (1)
hypercholesterolemia	2 (0.7)	0 (0)	2 (1)
deep vein thrombosis/pulmonary embolism	2 (0.7)	1 (1)	1 (0.5)
anemia	1 (0.3)	0 (0)	1 (0.5)
chronic venous insufficiency	1 (0.3)	1 (1)	0 (0)
thrombocitopenia	1 (0.3)	0 (0)	1 (0.5)
**Cerebral cardiovascular disease**	**4 (1.3)**	**1 (1)**	**3 (1.6)**
**Rheumatic/musculoskeletal disease and immune-mediated disease**	**50 (16.8)**	**9 (8.8)**	**41 (21.5)**
rheumatic disease	25 (8.4)	5 (4.9)	20 (10.5)
musculoskeletal pain	8 (2.7)	1 (1)	7 (3.7)
lupus erythematosus	6 (2)	1 (1)	5 (2.6)
joint pain	3 (1)	0 (0)	3 (1.6)
undifferentiated connective tissue disease	2 (0.7)	1 (1)	1 (0.5)
musculoskeletal disease	2 (0.7)	1 (1)	1 (0.5)
autoimmune disease	1 (0.3)	0 (0)	1 (0.5)
osteoporosis	1 (0.3)	0 (0)	1 (0.5)
crohn's disease	1 (0.3)	0 (0)	1 (0.5)
wrist synovitis	1 (0.3)	0 (0)	1 (0.5)
**Skin disease**	**41 (13.8)**	**18 (17.6)**	**22 (11.5)**
psoriasis	7 (2.4)	5 (4.9)	2 (1)
rosacea	3 (1)	2 (2)	1 (0.5)
pemphigus foliaceus	3 (1)	0 (0)	3 (1.6)
tinea cruris	2 (0.7)	0 (0)	2 (1)
skin burns	1 (0.3)	1 (1)	0 (0)
atopic dermatitis	1 (0.3)	1 (1)	0 (0)
dyshydrotic eczema	1 (0.3)	0 (0)	1 (0.5)
eczema	1 (0.3)	0 (0)	1 (0.5)
eczematous periocular dermatitis	1 (0.3)	0 (0)	1 (0.5)

erythematous facial dermatitis	1 (0.3)	1 (1)	0 (0)
folliculitis in the scalp	1 (0.3)	1 (1)	0 (0)
history of atopic dermatitis	1 (0.3)	1 (1)	0 (0)
neck descamation	1 (0.3)	1 (1)	0 (0)
necrotic crust	1 (0.3)	1 (1)	0 (0)
onycholysis	1 (0.3)	0 (0)	1 (0.5)
prurito	1 (0.3)	0 (0)	1 (0.5)
psoriasis	1 (0.3)	0 (0)	1 (0.5)
scabies	1 (0.3)	1 (1)	0 (0)
scleroderma	1 (0.3)	0 (0)	1 (0.5)
urticaria multiforme	1 (0.3)	0 (0)	0 (0)
vitiligo	1 (0.3)	0 (0)	1 (0.5)
dermatomyositis	1 (0.3)	0 (0)	1 (0.5)
acne	1 (0.3)	1 (1)	0 (0)
cutaneous sarcoidosis	1 (0.3)	0 (0)	1 (0.5)
granuloma annulare	1 (0.3)	0 (0)	1 (0.5)
hailey hailey disease	1 (0.3)	1 (1)	0 (0)
history of eczema	1 (0.3)	1 (1)	0 (0)
ichen planus	1 (0.3)	0 (0)	1 (0.5)
mucinosis	1 (0.3)	0 (0)	1 (0.5)
pemphigus vulgaris	1 (0.3)	0 (0)	1 (0.5)
**Cancer**	**34 (11.4)**	**15 (14.7)**	**19 (9.9)**
**Diabetes**	**19 (6.4)**	**6 (5.9)**	**13 (6.8)**
**Respiratory disease**	**16 (5.4)**	**6 (5.9)**	**10 (5.2)**
repiratory disease, sore throat	4 (1.3)	3 (2.9)	1 (0.5)
rhinitis	3 (1)	1 (1)	2 (1)
asthma	2 (0.7)	0 (0)	2 (1)
COPD	2 (0.7)	0 (0)	2 (1)
sinusits	2 (0.7)	1 (1)	1 (0.5)
common cold	1 (0.3)	0 (0)	1 (0.5)
coryzal symptoms	1 (0.3)	0 (0)	1 (0.5)
rhinorrhea	1 (0.3)	1 (1)	0 (0)
**Pregnancy/labour**	**6 (2)**	**0 (0)**	**6 (3.1)**
**Neurological disease**	**16 (5.4)**	**6 (5.9)**	**10 (5.2)**
other/unespecified neurological disease	3 (1)	1 (1)	2 (1)
depression	2 (0.7)	1 (1)	1 (0.5)
parkinson	2 (0.7)	1 (1)	1 (0.5)
neurological disorder, seizure	2 (0.7)	1 (1)	1 (0.5)
androgenic alopecia	1 (0.3)	1 (1)	0 (0)
carpal tunner syndrome	1 (0.3)	0 (0)	1 (0.5)
dementia	1 (0.3)	0 (0)	1 (0.5)
epilepsy	1 (0.3)	1 (1)	0 (0)

merkerson-rosenthal	1 (0.3)	0 (0)	1 (0.5)
migraine	1 (0.3)	0 (0)	1 (0.5)
schizophrenia	1 (0.3)	0 (0)	1 (0.5)
**Anorexia/eating disorder**	**2 (0.7)**	**0 (0)**	**2 (1)**
**Hormonal disease**	**6 (2)**	**1 (1)**	**5 (2.6)**
hormonal disease, hypothyroidism	5 (1.7)	1 (1)	4 (2.1)
hormonal disease, cushing's syndrome	1 (0.3)	0 (0)	1 (0.5)
**Kidney disease**	**11 (3.7)**	**4 (3.9)**	**6 (3.1)**
**Gastrointestinal disease**	**8 (2.7)**	**2 (2)**	**6 (3.1)**
**Abscess/cyst**	**6 (2)**	**2 (2)**	**4 (2.1)**
abscess	2 (0.7)	2 (2)	0 (0)
bartholin's cyst	1 (0.3)	0 (0)	1 (0.5)
facial	1 (0.3)	0 (0)	1 (0.5)
polycystic ovary syndrome	1 (0.3)	0 (0)	1 (0.5)
gastrointestinal disease, abscess	1 (0.3)	0 (0)	1 (0.5)
**Liver disease**	**1 (0.3)**	**0 (0)**	**1 (0.5)**
**Drug reaction**	**2 (0.7)**	**0 (0)**	**2 (1)**
drug reaction, guillain-barre syndrome	1 (0.3)	0 (0)	1 (0.5)
drug reaction, stevens-johnson syndrome	1 (0.3)	0 (0)	1 (0.5)
**Fever**	**4 (1.3)**	**2 (2)**	**2 (1)**
**Recent bite dog/spider/tick**	**3 (1)**	**1 (1)**	**2 (1)**
recent dog bite	1 (0.3)	0 (0)	1 (0.5)
recent spider bite	1 (0.3)	1 (1)	0 (0)
recent tick bite	1 (0.3)	0 (0)	1 (0.5)
**Other**	**28 (9.4)**	**9 (8.8)**	**19 (9.9)**
recent surgery	9 (3)	3 (2.9)	6 (3.1)
allergic reaction	3 (1)	1 (1)	2 (1)
obesity	3 (1)	0 (0)	3 (1.6)
cellulitis	2 (0.7)	1 (1)	1 (0.5)
macular degeneration	2 (0.7)	0 (0)	2 (1)
prostatic disease	2 (0.7)	2 (2)	0 (0)
alcohol/drug abuse	1 (0.3)	1 (1)	0 (0)
bladder disease	1 (0.3)	1 (1)	0 (0)
metabolic syndrome	1 (0.3)	0 (0)	1 (0.5)
monoclonal gammopathy	1 (0.3)	0 (0)	1 (0.5)
other	1 (0.3)	0 (0)	1 (0.5)
protein deficiency	1 (0.3)	0 (0)	1 (0.5)
weight control	1 (0.3)	0 (0)	1 (0.5)
**Missing/unknown**	**52 (17.5)**	**17 (16.7)**	**33 (17.3)**

Results as counts and percentage of total number of suspect drugs in the corresponding group. Abbreviations: ATC Anatomical Therapeutic Chemical Classification System.

Table S2. Treatment for the Acute Generalized Exanthematous Pustulosis (AGEP) reaction.

AGEP treatment	Overall (n=460)	Men (n=169)	Women (n=286)
Corticosteroids for systemic use	144 (31.3)	53 (31.4)	89 (31.1)
Dermatologicals (antifungals, amollients, antipsoriatics, antibiotics and chemotherapeutics for dermatological use)	121 (26.3)	39 (23.1)	81 (28.3)
Corticosteroids, dermatological preparations	103 (22.4)	35 (20.7)	67 (23.4)
Antiinfectives for systemic use	62 (13.5)	22 (13)	40 (14)
Antibacterials for systemic use	*53 (11.5)*	*21 (12.4)*	*32 (11.2)*
Immune sera and immunoglobulins	*4 (0.9)*	*0 (0)*	*4 (1.4)*
Antimycotics for systemic use	*2 (0.4)*	*0 (0)*	*2 (0.7)*
Antivirals for systemic use	*2 (0.4)*	*1 (0.6)*	*1 (0.3)*
Antimycobacterials	*1 (0.2)*	*0 (0)*	*1 (0.3)*
Antihistamines for systemic use	21 (4.6)	9 (5.3)	11 (3.8)
Nervous system (analgesics, anxiolytics, antidepressnats)	20 (4.3)	9 (5.3)	10 (3.5)
Immunomosuppressants	13 (2.8)	2 (1.2)	11 (3.8)
Other	28 (6.1)	9 (5.3)	19 (6.6)
Cardiovascular system (adrenergic and dopaminergic agents; diuretics)	*5 (1.1)*	*2 (1.2)*	*3 (1)*
Anti-acne preparations	*5 (1.1)*	*0 (0)*	*5 (1.7)*
Alimentary tract and metabolism	*4 (0.9)*	*2 (1.2)*	*2 (0.7)*
Blood and blood forming organs (albumin)	*2 (0.4)*	*1 (0.6)*	*1 (0.3)*
Gynecological antiinfectives and antiseptives,	*2 (0.4)*	*0 (0)*	*2 (0.7)*
Antiinflammatory and antirheumatic products	*2 (0.4)*	*1 (0.6)*	*1 (0.3)*
Pituitary and hypothalamic hormones and analogues	*1 (0.2)*	*0 (0)*	*1 (0.3)*
Missing ATC code	*7 (1.5)*	*3 (1.8)*	*4 (1.4)*

Abbreviations: ATC Anatomical Therapeutic Chemical Classification System.

Table S3. Suspect drugs. Information from 297 patients, who can contribute with more than one drug each. This table complements Table 3.

Suspect drugs	Overall 344	Men 127	Women 210
**ALIMENTARY TRACT AND METABOLISM**	**6 (1.7)**	**1 (0.8)**	**5 (2.4)**
**STOMATOLOGICAL PREPARATIONS**	**1 (0.3)**	**0 (0)**	**1 (0.5)**
Antiinfectives and antiseptics for local oral treatment	1 (0.3)	0 (0)	1 (0.5)
miconazole	1 (0.3)	0 (0)	1 (0.5)
**DRUGS FOR ACID RELATED DISORDERS**	**1 (0.3)**	**1 (0.8)**	**0 (0)**
H2-receptor antagonists	1 (0.3)	1 (0.8)	0 (0)
ranitidine	1 (0.3)	1 (0.8)	0 (0)
**DIGESTIVES, INCL. ENZYMES**	**1 (0.3)**	**0 (0)**	**1 (0.5)**
Enzyme preparations	1 (0.3)	0 (0)	1 (0.5)
**DRUGS USED IN DIABETES**	**2 (0.6)**	**0 (0)**	**2 (0.9)**
Sulfonamides, urea derivatives	1 (0.3)	0 (0)	1 (0.5)
gliclazide	1 (0.3)	0 (0)	1 (0.5)
Alpha glucosidase inhibitors	1 (0.3)	0 (0)	1 (0.5)
acarbose	1 (0.3)	0 (0)	1 (0.5)
**VITAMINS**	**1 (0.3)**	**0 (0)**	**1 (0.5)**
Ascorbic acid (vitamin C), plain	1 (0.3)	0 (0)	1 (0.5)
ascorbic acid (vit C)	1 (0.3)	0 (0)	1 (0.5)
**BLOOD AND BLOOD FORMING ORGANS**	**8 (2.3)**	**3 (2.4)**	**5 (2.4)**
**ANTITHROMBOTIC AGENTS**	**6 (1.7)**	**2 (1.6)**	**4 (1.9)**
Heparin group	4 (1.2)	1 (0.8)	3 (1.4)
enoxaparin	3 (0.9)	1 (0.8)	2 (0.9)
bemiparin	1 (0.3)	0 (0)	1 (0.5)
Direct thrombin inhibitors	1 (0.3)	1 (0.8)	0 (0)
dabigatran etexilate	1 (0.3)	1 (0.8)	0 (0)
Direct factor Xa inhibitors	1 (0.3)	0 (0)	1 (0.5)
apixaban	1 (0.3)	0 (0)	1 (0.5)
**ANTIANEMIC PREPARATIONS**	**2 (0.6)**	**1 (0.8)**	**1 (0.5)**
Iron, parenteral preparations	1 (0.3)	1 (0.8)	0 (0)
Other antianemic preparations	1 (0.3)	0 (0)	1 (0.5)
**CARDIOVASCULAR SYSTEM**	**9 (2.6)**	**2 (1.6)**	**6 (2.8)**
**CARDIAC THERAPY**	**1 (0.3)**	**0 (0)**	**1 (0.5)**
Antiarrhythmics, class III	1 (0.3)	0 (0)	1 (0.5)
amiodarone	1 (0.3)	0 (0)	1 (0.5)
**DIURETICS**	**1 (0.3)**	**0 (0)**	**1 (0.5)**
Thiazides, plain	1 (0.3)	0 (0)	1 (0.5)
hydrochlorothiazide	1 (0.3)	0 (0)	1 (0.5)
**BETA BLOCKING AGENTS**	**1 (0.3)**	**0 (0)**	**0 (0)**
Alpha and beta blocking agents	1 (0.3)	0 (0)	0 (0)

labetalol	1 (0.3)	0 (0)	0 (0)
**CALCIUM CHANNEL BLOCKERS**	**4 (1.2)**	**2 (1.6)**	**2 (0.9)**
Benzothiazepine derivatives	4 (1.2)	2 (1.6)	2 (0.9)
diltiazem	4 (1.2)	2 (1.6)	2 (0.9)
**AGENTS ACTING ON THE RENIN-ANGIOTENSIN SYSTEM**	**1 (0.3)**	**0 (0)**	**1 (0.5)**
Angiotensin II antagonists, plain	1 (0.3)	0 (0)	1 (0.5)
losartan	1 (0.3)	0 (0)	1 (0.5)
**LIPID MODIFYING AGENTS**	**1 (0.3)**	**0 (0)**	**1 (0.5)**
Fibrates	1 (0.3)	0 (0)	1 (0.5)
fenofibrate	1 (0.3)	0 (0)	1 (0.5)
**DERMATOLOGICALS**	**10 (2.9)**	**3 (2.4)**	**7 (3.3)**
**ANTIFUNGALS FOR DERMATOLOGICAL USE**	**10 (2.9)**	**3 (2.4)**	**7 (3.3)**
ANTIFUNGALS FOR TOPICAL USE	2 (0.6)	0 (0)	2 (0.9)
nystatin	3 (0.9)	0 (0)	3 (1.4)
ANTIFUNGALS FOR SYSTEMIC USE	8 (2.3)	3 (2.4)	5 (2.4)
Antifungals for systemic use	8 (2.3)	3 (2.4)	5 (2.4)
terbinafine	8 (2.3)	3 (2.4)	5 (2.4)
**GENITO URINARY SYSTEM AND SEX HORMONES**	**3 (0.9)**	**1 (0.8)**	**2 (0.9)**
**GYNECOLOGICAL ANTIINFECTIVES AND ANTISEPTICS**	**1 (0.3)**	**1 (0.8)**	**0 (0)**
Triazole derivatives	1 (0.3)	1 (0.8)	0 (0)
**OTHER GYNECOLOGICALS**	**1 (0.3)**	**0 (0)**	**1 (0.5)**
Sympathomimetics, labour repressants	1 (0.3)	0 (0)	1 (0.5)
ritodrine	1 (0.3)	0 (0)	1 (0.5)
**SEX HORMONES AND MODULATORS OF THE GENITAL SYSTEM**	**1 (0.3)**	**0 (0)**	**1 (0.5)**
Progesterone receptor modulators	1 (0.3)	0 (0)	1 (0.5)
mifepristone	1 (0.3)	0 (0)	1 (0.5)
**UROLOGICALS**	**1 (0.3)**	**1 (0.8)**	**0 (0)**
Testosterone-5-alpha reductase inhibitors	1 (0.3)	1 (0.8)	0 (0)
finasteride	1 (0.3)	1 (0.8)	0 (0)
**SYSTEMIC HORMONAL PREPARATIONS, EXCL. SEX HORMONES AND INSULINS**	**3 (0.9)**	**1 (0.8)**	**2 (0.9)**
**CORTICOSTEROIDS FOR SYSTEMIC USE**	**3 (0.9)**	**1 (0.8)**	**2 (0.9)**
Glucocorticoids	3 (0.9)	1 (0.8)	2 (0.9)
betamethasone	1 (0.3)	0 (0)	1 (0.5)
dexamethasone	1 (0.3)	0 (0)	1 (0.5)
prednisolone	1 (0.3)	1 (0.8)	0 (0)
**ANTIINFECTIVES FOR SYSTEMIC USE**	**126 (36.5)**	**55 (43.3)**	**66 (31.3)**
**ANTIBACTERIALS FOR SYSTEMIC USE**	**107 (31)**	**45 (35.4)**	**60 (28.4)**
TETRACYCLINES	2 (0.6)	1 (0.8)	1 (0.5)
Tetracyclines	2 (0.6)	1 (0.8)	1 (0.5)
doxycycline	1 (0.3)	0 (0)	1 (0.5)
tigecycline	1 (0.3)	1 (0.8)	0 (0)
BETA-LACTAM ANTIBACTERIALS, PENICILLINS	39 (11.3)	14 (11)	25 (11.8)

Penicillins with extended spectrum	13 (3.8)	5 (3.9)	8 (3.8)
amoxicillin	11 (3.2)	5 (3.9)	6 (2.8)
piperacillin	1 (0.3)	0 (0)	1 (0.5)
ampicillin, combinations	1 (0.3)	0 (0)	1 (0.5)
Beta-lactamase sensitive penicillins	1 (0.3)	1 (0.8)	0 (0)
benzylpenicillin	1 (0.3)	1 (0.8)	0 (0)
Beta-lactamase resistant penicillins	6 (1.7)	3 (2.4)	3 (1.4)
dicloxacillin	1 (0.3)	1 (0.8)	0 (0)
cloxacillin	1 (0.3)	1 (0.8)	0 (0)
oxacillin	1 (0.3)	0 (0)	1 (0.5)
flucloxacillin	3 (0.9)	1 (0.8)	2 (0.9)
Beta-lactamase inhibitors	1 (0.3)	0 (0)	1 (0.5)
sulbactam	1 (0.3)	0 (0)	1 (0.5)
Combinations of penicillins, incl. beta-lactamase inhibitors	18 (5.2)	5 (3.9)	13 (6.2)
ampicillin and enzyme inhibitor	1 (0.3)	1 (0.8)	0 (0)
amoxicillin and enzyme inhibitor	13 (3.8)	3 (2.4)	10 (4.7)
piperacillin and enzyme inhibitor	4 (1.2)	1 (0.8)	3 (1.4)
OTHER BETA-LACTAM ANTIBACTERIALS	24 (7)	10 (7.9)	13 (6.2)
First-generation cephalosporins	3 (0.9)	1 (0.8)	2 (0.9)
cefalexin	1 (0.3)	0 (0)	1 (0.5)
cefazolin	2 (0.6)	1 (0.8)	1 (0.5)
Second-generation cephalosporins	1 (0.3)	1 (0.8)	0 (0)
cefprozil	1 (0.3)	1 (0.8)	0 (0)
Third-generation cephalosporins	13 (3.8)	5 (3.9)	7 (3.3)
cefotaxime	1 (0.3)	0 (0)	1 (0.5)
ceftazidime	1 (0.3)	1 (0.8)	0 (0)
ceftriaxone	8 (2.3)	4 (3.1)	3 (1.4)
cefoperazone	1 (0.3)	0 (0)	1 (0.5)
cefdinir	1 (0.3)	0 (0)	1 (0.5)
cefditoren	1 (0.3)	0 (0)	1 (0.5)
Fourth-generation cephalosporins	3 (0.9)	2 (1.6)	1 (0.5)
cefepime	3 (0.9)	2 (1.6)	1 (0.5)
Carbapenems	3 (0.9)	1 (0.8)	2 (0.9)
meropenem	2 (0.6)	1 (0.8)	1 (0.5)
ertapenem	1 (0.3)	0 (0)	1 (0.5)
SULFONAMIDES AND TRIMETHOPRIM	8 (2.3)	6 (4.7)	2 (0.9)
sulfamethoxazole and trimethoprim	8 (2.3)	6 (4.7)	2 (0.9)
MACROLIDES, LINCOSAMIDES AND STREPTOGRAMINS	15 (4.3)	6 (4.7)	9 (4.3)
Macrolides	5 (1.4)	4 (3.1)	1 (0.5)
erythromycin	2 (0.6)	1 (0.8)	1 (0.5)
josamycin	1 (0.3)	1 (0.8)	0 (0)
azithromycin	2 (0.6)	2 (1.6)	0 (0)

Lincosamides	9 (2.6)	2 (1.6)	7 (3.3)
clindamycin	8 (2.3)	2 (1.6)	6 (2.8)
lincomycin	1 (0.3)	0 (0)	1 (0.5)
Streptogramins	1 (0.3)	0 (0)	1 (0.5)
pristinamycin	1 (0.3)	0 (0)	1 (0.5)
QUINOLONE ANTIBACTERIALS	5 (1.4)	3 (2.4)	2 (0.9)
Fluoroquinolones	5 (1.4)	3 (2.4)	2 (0.9)
ciprofloxacin	2 (0.6)	2 (1.6)	0 (0)
levofloxacin	2 (0.6)	1 (0.8)	1 (0.5)
tosufloxacin	1 (0.3)	0 (0)	1 (0.5)
OTHER ANTIBACTERIALS	14 (4.1)	5 (3.9)	8 (3.8)
Glycopeptide antibacterials	10 (2.9)	2 (1.6)	7 (3.3)
vancomycin	7 (2)	2 (1.6)	4 (1.9)
teicoplanin	2 (0.6)	0 (0)	2 (0.9)
telavancin	1 (0.3)	0 (0)	1 (0.5)
metronidazole	2 (0.6)	1 (0.8)	1 (0.5)
Other antibacterials	2 (0.6)	2 (1.6)	0 (0)
daptomycin	2 (0.6)	2 (1.6)	0 (0)
**ANTIMYCOTICS FOR SYSTEMIC USE**	**1 (0.3)**	**1 (0.8)**	**0 (0)**
fluconazole	1 (0.3)	1 (0.8)	0 (0)
**ANTIMYCOBACTERIALS**	**1 (0.3)**	**0 (0)**	**1 (0.5)**
rifampicin	1 (0.3)	0 (0)	1 (0.5)
**ANTIVIRALS FOR SYSTEMIC USE**	**8 (2.3)**	**7 (5.5)**	**1 (0.5)**
Nucleosides and nucleotides excl. reverse transcriptase inhibitors	1 (0.3)	0 (0)	1 (0.5)
aciclovir	1 (0.3)	0 (0)	1 (0.5)
Neuraminidase inhibitors	1 (0.3)	1 (0.8)	0 (0)
oseltamivir	1 (0.3)	1 (0.8)	0 (0)
Antivirals for treatment of HCV infections	2 (0.6)	2 (1.6)	0 (0)
ribavirin	2 (0.6)	2 (1.6)	0 (0)
Antivirals for treatment of HIV infections, combinations	2 (0.6)	2 (1.6)	0 (0)
lopinavir-ritonavir,kaletra	2 (0.6)	2 (1.6)	0 (0)
Other antivirals	2 (0.6)	2 (1.6)	0 (0)
favipiravir	2 (0.6)	2 (1.6)	0 (0)
**VACCINES**	**9 (2.6)**	**2 (1.6)**	**4 (1.9)**
**BACTERIAL VACCINES**	**2 (0.6)**	**0 (0)**	**0 (0)**
Pertussis vaccines	1 (0.3)	0 (0)	0 (0)
pertussis, purified antigen, combinations with toxoids	1 (0.3)	0 (0)	0 (0)
Pneumococcal vaccines	1 (0.3)	0 (0)	0 (0)
pneumococcus, purified polysaccharides antigen	1 (0.3)	0 (0)	0 (0)
VIRAL VACCINES	7 (2)	2 (1.6)	4 (1.9)
Influenza vaccines	1 (0.3)	0 (0)	1 (0.5)
Measles vaccines	1 (0.3)	0 (0)	0 (0)

measles, combinations with mumps and rubella, live attenuated	1 (0.3)	0 (0)	0 (0)
Other viral vaccines	5 (1.4)	2 (1.6)	3 (1.4)
covid-19 vaccines	5 (1.4)	2 (1.6)	3 (1.4)
**ANTINEOPLASTIC AND IMMUNOMODULATING AGENTS**	**50 (14.5)**	**18 (14.2)**	**33 (15.6)**
**ANTINEOPLASTIC AGENTS**	**42 (12.2)**	**15 (11.8)**	**28 (13.3)**
PLANT ALKALOIDS AND OTHER NATURAL PRODUCTS	3 (0.9)	1 (0.8)	2 (0.9)
Taxanes	3 (0.9)	1 (0.8)	2 (0.9)
paclitaxel	1 (0.3)	1 (0.8)	0 (0)
docetaxel	2 (0.6)	0 (0)	2 (0.9)
CYTOTOXIC ANTIBIOTICS AND RELATED SUBSTANCES	1 (0.3)	1 (0.8)	0 (0)
Other cytotoxic antibiotics	1 (0.3)	1 (0.8)	0 (0)
bleomycin	1 (0.3)	1 (0.8)	0 (0)
PROTEIN KINASE INHIBITORS	14 (4.1)	3 (2.4)	11 (5.2)
imatinib	5 (1.4)	0 (0)	5 (2.4)
gefitinib	2 (0.6)	1 (0.8)	1 (0.5)
erlotinib	2 (0.6)	1 (0.8)	1 (0.5)
vemurafenib	1 (0.3)	1 (0.8)	0 (0)
lapatinib	1 (0.3)	0 (0)	1 (0.5)
sorafenib	3 (0.9)	0 (0)	3 (1.4)
OTHER ANTINEOPLASTIC AGENTS	24 (7)	10 (7.9)	14 (6.6)
Platinum compounds	1 (0.3)	1 (0.8)	0 (0)
carboplatin	1 (0.3)	1 (0.8)	0 (0)
Monoclonal antibodies	8 (2.3)	6 (4.7)	2 (0.9)
rituximab	2 (0.6)	0 (0)	2 (0.9)
cetuximab	1 (0.3)	1 (0.8)	0 (0)
ipilimumab	2 (0.6)	2 (1.6)	0 (0)
nivolumab	1 (0.3)	1 (0.8)	0 (0)
pembrolizumab	1 (0.3)	1 (0.8)	0 (0)
atezolizumab	1 (0.3)	1 (0.8)	0 (0)
Protein kinase inhibitors	14 (4.1)	3 (2.4)	11 (5.2)
vismodegib	1 (0.3)	0 (0)	1 (0.5)
Other antineoplastic agents	1 (0.3)	0 (0)	1 (0.5)
**ENDOCRINE THERAPY**	**1 (0.3)**	**1 (0.8)**	**0 (0)**
HORMONE ANTAGONISTS AND RELATED AGENTS	1 (0.3)	1 (0.8)	0 (0)
Anti-androgens	1 (0.3)	1 (0.8)	0 (0)
enzalutamide	1 (0.3)	1 (0.8)	0 (0)
**IMMUNOSTIMULANTS**	**1 (0.3)**	**1 (0.8)**	**0 (0)**
Interferons	1 (0.3)	1 (0.8)	0 (0)
interferon beta natural	1 (0.3)	1 (0.8)	0 (0)
**IMMUNOSUPPRESSANTS**	**6 (1.7)**	**1 (0.8)**	**5 (2.4)**
Selective immunosuppressants	1 (0.3)	0 (0)	1 (0.5)
mycophenolic acid	1 (0.3)	0 (0)	1 (0.5)

Tumor necrosis factor alpha (TNF-α) inhibitors	2 (0.6)	1 (0.8)	1 (0.5)
etanercept	1 (0.3)	1 (0.8)	0 (0)
infliximab	1 (0.3)	0 (0)	1 (0.5)
Interleukin inhibitors	1 (0.3)	0 (0)	1 (0.5)
tocilizumab	1 (0.3)	0 (0)	1 (0.5)
Other immunosuppressants	2 (0.6)	0 (0)	2 (0.9)
azathioprine	2 (0.6)	0 (0)	2 (0.9)
**MUSCULO-SKELETAL SYSTEM**	**20 (5.8)**	**5 (3.9)**	**15 (7.1)**
**ANTIINFLAMMATORY AND ANTIRHEUMATIC PRODUCTS**	**18 (5.2)**	**4 (3.1)**	**14 (6.6)**
Acetic acid derivatives and related substances	3 (0.9)	1 (0.8)	2 (0.9)
etodolac	2 (0.6)	1 (0.8)	1 (0.5)
acemetacin	1 (0.3)	0 (0)	1 (0.5)
Oxicams	2 (0.6)	0 (0)	2 (0.9)
piroxicam	1 (0.3)	0 (0)	1 (0.5)
lornoxicam	1 (0.3)	0 (0)	1 (0.5)
Propionic acid derivatives	5 (1.4)	1 (0.8)	4 (1.9)
ibuprofen	4 (1.2)	1 (0.8)	3 (1.4)
flurbiprofen	1 (0.3)	0 (0)	1 (0.5)
Coxibs	6 (1.7)	2 (1.6)	4 (1.9)
celecoxib	4 (1.2)	1 (0.8)	3 (1.4)
valdecoxib	1 (0.3)	0 (0)	1 (0.5)
etoricoxib	1 (0.3)	1 (0.8)	0 (0)
Other antiinflammatory and antirheumatic agents, non-steroids	2 (0.6)	0 (0)	2 (0.9)
nimesulide	1 (0.3)	0 (0)	1 (0.5)
chondroitin sulfate	1 (0.3)	0 (0)	1 (0.5)
**MUSCLE RELAXANTS**	**1 (0.3)**	**0 (0)**	**1 (0.5)**
Other centrally acting agents	1 (0.3)	0 (0)	1 (0.5)
eperisone	1 (0.3)	0 (0)	1 (0.5)
**ANTIGOUT PREPARATIONS**	**1 (0.3)**	**1 (0.8)**	**0 (0)**
Preparations inhibiting uric acid production	1 (0.3)	1 (0.8)	0 (0)
allopurinol	1 (0.3)	1 (0.8)	0 (0)
**NERVOUS SYSTEM**	**28 (8.1)**	**15 (11.8)**	**13 (6.2)**
**ANESTHETICS**	**2 (0.6)**	**0 (0)**	**2 (0.9)**
Amides	2 (0.6)	0 (0)	2 (0.9)
mepivacaine	1 (0.3)	0 (0)	1 (0.5)
cinchocaine	1 (0.3)	0 (0)	1 (0.5)
**ANALGESICS**	**10 (2.9)**	**6 (4.7)**	**4 (1.9)**
OPIOIDS	2 (0.6)	1 (0.8)	1 (0.5)
Natural opium alkaloids	2 (0.6)	1 (0.8)	1 (0.5)
morphine	2 (0.6)	1 (0.8)	1 (0.5)
OTHER ANALGESICS AND ANTIPYRETICS	8 (2.3)	5 (3.9)	3 (1.4)
Salicylic acid and derivatives	1 (0.3)	1 (0.8)	0 (0)

acetylsalicylic acid	1 (0.3)	1 (0.8)	0 (0)
Pyrazolones	1 (0.3)	1 (0.8)	0 (0)
metamizole sodium	1 (0.3)	1 (0.8)	0 (0)
Anilides	6 (1.7)	3 (2.4)	3 (1.4)
paracetamol	6 (1.7)	3 (2.4)	3 (1.4)
**ANTIEPILEPTICS**	**6 (1.7)**	**3 (2.4)**	**3 (1.4)**
Hydantoin derivatives	1 (0.3)	1 (0.8)	0 (0)
phenytoin	1 (0.3)	1 (0.8)	0 (0)
Carboxamide derivatives	1 (0.3)	1 (0.8)	0 (0)
carbamazepine	1 (0.3)	1 (0.8)	0 (0)
Fatty acid derivatives	1 (0.3)	0 (0)	1 (0.5)
valproic acid	1 (0.3)	0 (0)	1 (0.5)
Other antiepileptics	3 (0.9)	1 (0.8)	2 (0.9)
lamotrigine	1 (0.3)	0 (0)	1 (0.5)
levetiracetam	1 (0.3)	0 (0)	1 (0.5)
cannabidiol	1 (0.3)	1 (0.8)	0 (0)
**PSYCHOLEPTICS**	**4 (1.2)**	**3 (2.4)**	**1 (0.5)**
ANTIPSYCHOTICS	2 (0.6)	1 (0.8)	1 (0.5)
Diazepines, oxazepines, thiazepines and oxepines	2 (0.6)	1 (0.8)	1 (0.5)
olanzapine	2 (0.6)	1 (0.8)	1 (0.5)
ANXIOLYTICS	2 (0.6)	2 (1.6)	0 (0)
Diphenylmethane derivatives	2 (0.6)	2 (1.6)	0 (0)
hydroxyzine	2 (0.6)	2 (1.6)	0 (0)
**PSYCHOANALEPTICS**	**4 (1.2)**	**2 (1.6)**	**2 (0.9)**
ANTIDEPRESSANTS	3 (0.9)	1 (0.8)	2 (0.9)
Selective serotonin reuptake inhibitors	2 (0.6)	1 (0.8)	1 (0.5)
paroxetine	1 (0.3)	0 (0)	1 (0.5)
sertraline	1 (0.3)	1 (0.8)	0 (0)
Other antidepressants	1 (0.3)	0 (0)	1 (0.5)
bupropion	1 (0.3)	0 (0)	1 (0.5)
PSYCHOSTIMULANTS, AGENTS USED FOR ADHD AND NOOTROPICS	1 (0.3)	1 (0.8)	0 (0)
Centrally acting sympathomimetics	1 (0.3)	1 (0.8)	0 (0)
methylphenidate	1 (0.3)	1 (0.8)	0 (0)
**OTHER NERVOUS SYSTEM DRUGS**	**2 (0.6)**	**1 (0.8)**	**1 (0.5)**
Drugs used in nicotine dependence	2 (0.6)	1 (0.8)	1 (0.5)
varenicline	2 (0.6)	1 (0.8)	1 (0.5)
**ANTIPARASITIC PRODUCTS, INSECTICIDES AND REPELLENTS**	**50 (14.5)**	**12 (9.4)**	**38 (18)**
**ANTIPROTOZOALS**	**48 (13.9)**	**10 (7.9)**	**38 (18)**
AGENTS AGAINST AMOEBIASIS AND OTHER PROTOZOAL DISEASES	1 (0.3)	1 (0.8)	0 (0)
Nitroimidazole derivatives	1 (0.3)	1 (0.8)	0 (0)
ANTIMALARIALS	46 (13.3)	8 (6.3)	38 (18)
Aminoquinolines	45 (13)	8 (6.3)	37 (17.5)

chloroquine	1 (0.3)	1 (0.8)	0 (0)
hydroxychloroquine	44 (12.8)	7 (5.5)	37 (17.5)
Biguanides	1 (0.3)	0 (0)	1 (0.5)
proguanil, combinations	1 (0.3)	0 (0)	1 (0.5)
AGENTS AGAINST LEISHMANIASIS AND TRYPANOSOMIASIS	1 (0.3)	1 (0.8)	0 (0)
benznidazole	1 (0.3)	1 (0.8)	0 (0)
**ANTHELMINTICS**	**1 (0.3)**	**1 (0.8)**	**0 (0)**
ANTITREMATODALS	1 (0.3)	1 (0.8)	0 (0)
Quinoline derivatives and related substances	1 (0.3)	1 (0.8)	0 (0)
praziquantel	1 (0.3)	1 (0.8)	0 (0)
**ECTOPARASITICIDES, INCL. SCABICIDES**	**1 (0.3)**	**1 (0.8)**	**0 (0)**
Chlorine containing products	1 (0.3)	1 (0.8)	0 (0)
lindane	1 (0.3)	1 (0.8)	0 (0)
**RESPIRATORY SYSTEM**	**7 (2)**	**2 (1.6)**	**5 (2.4)**
**NASAL PREPARATIONS**	**1 (0.3)**	**0 (0)**	**1 (0.5)**
Sympathomimetics	1 (0.3)	0 (0)	1 (0.5)
pseudoephedrine	1 (0.3)	0 (0)	1 (0.5)
**COUGH AND COLD PREPARATIONS**	**3 (0.9)**	**1 (0.8)**	**2 (0.9)**
Mucolytics	1 (0.3)	0 (0)	1 (0.5)
carbocisteine	1 (0.3)	0 (0)	1 (0.5)
Opium alkaloids and derivatives	2 (0.6)	1 (0.8)	1 (0.5)
pholcodine	1 (0.3)	0 (0)	1 (0.5)
dextromethorphan	1 (0.3)	1 (0.8)	0 (0)
**ANTIHISTAMINES FOR SYSTEMIC USE**	**3 (0.9)**	**1 (0.8)**	**2 (0.9)**
Phenothiazine derivatives	1 (0.3)	1 (0.8)	0 (0)
mequitazine	1 (0.3)	1 (0.8)	0 (0)
Piperazine derivatives	1 (0.3)	0 (0)	1 (0.5)
cetirizine	1 (0.3)	0 (0)	1 (0.5)
Other antihistamines for systemic use	1 (0.3)	0 (0)	1 (0.5)
fexofenadine	1 (0.3)	0 (0)	1 (0.5)
**SENSORY ORGANS**	**1 (0.3)**	**0 (0)**	**1 (0.5)**
**OPHTHALMOLOGICALS**	**1 (0.3)**	**0 (0)**	**1 (0.5)**
Antineovascularisation agents	1 (0.3)	0 (0)	1 (0.5)
ranibizumab	1 (0.3)	0 (0)	1 (0.5)
**VARIOUS**	**38 (11)**	**13 (10.2)**	**24 (11.4)**
**CONTRAST MEDIA**	**38 (11)**	**13 (10.2)**	**24 (11.4)**
X-RAY CONTRAST MEDIA, IODINATED	37 (10.7)	13 (10.2)	23 (10.9)
Watersoluble, nephrotropic, low osmolar X-ray contrast media	37 (10.7)	13 (10.2)	23 (10.9)
iohexol	6 (1.7)	2 (1.6)	4 (1.9)
iopamidol	1 (0.3)	0 (0)	1 (0.5)
iopromide	3 (0.9)	1 (0.8)	2 (0.9)
ioversol	5 (1.4)	3 (2.4)	2 (0.9)

iodixanol	11 (3.2)	4 (3.1)	7 (3.3)
iomeprol	10 (2.9)	3 (2.4)	6 (2.8)
iobitridol	1 (0.3)	0 (0)	1 (0.5)
MAGNETIC RESONANCE IMAGING CONTRAST MEDIA	1 (0.3)	0 (0)	1 (0.5)
Paramagnetic contrast media	1 (0.3)	0 (0)	1 (0.5)

Results as counts and percentage of total number of suspect drugs in the corresponding group. The following drugs, reported in one case report each, were not included due to missing Anatomical Therapeutic Chemical Classification System (ATC) code: curcumin, Neurotropin, probiotics, traditional Chinese medicine, and dai-kenchu-to herbal medicine. *Note that hydroxychloroquine has been classified as antimalarial following the ATC system, however, it was indicated for lupus, rheumatoid arthritis, other rheumatic and immune-mediating diseases, and COVID-19.

Table S4. All reported drugs, independently of being or not suspect. Information from 297 patients, who can contribute with more than one drug each. This table complements Table 4.

Drugs	Overall	Men	Women
All reported drugs	(n=716)	(n=265)	(n=441)
**ALIMENTARY TRACT AND METABOLISM**	**49 (6.8)**	**13 (4.9)**	**36 (8.2)**
**STOMATOLOGICAL PREPARATIONS**	**1 (0.1)**	**0 (0)**	**1 (0.2)**
Antiinfectives and antiseptics for local oral treatment	1 (0.1)	0 (0)	1 (0.2)
miconazole	1 (0.1)	0 (0)	1 (0.2)
**DRUGS FOR ACID RELATED DISORDERS**	**16 (2.2)**	**6 (2.3)**	**10 (2.3)**
ANTACIDS	1 (0.1)	1 (0.4)	0 (0)
Magnesium compounds	1 (0.1)	1 (0.4)	0 (0)
magnesium oxide	1 (0.1)	1 (0.4)	0 (0)
DRUGS FOR PEPTIC ULCER AND GASTRO-OESOPHAGEAL REFLUX DISEASE	15 (2.1)	5 (1.9)	10 (2.3)
H2-receptor antagonists	3 (0.4)	2 (0.8)	1 (0.2)
cimetidine	1 (0.1)	1 (0.4)	0 (0)
ranitidine	1 (0.1)	1 (0.4)	0 (0)
famotidine	1 (0.1)	0 (0)	1 (0.2)
Proton pump inhibitors	11 (1.5)	2 (0.8)	9 (2)
omeprazole	3 (0.4)	0 (0)	3 (0.7)
pantoprazole	5 (0.7)	1 (0.4)	4 (0.9)
lansoprazole	2 (0.3)	1 (0.4)	1 (0.2)
esomeprazole	1 (0.1)	0 (0)	1 (0.2)
Combinations for eradication of Helicobacter pylori	1 (0.1)	1 (0.4)	0 (0)
vonoprazan, amoxicillin and clarithromycin	1 (0.1)	1 (0.4)	0 (0)
**DRUGS FOR FUNCTIONAL GASTROINTESTINAL DISORDERS**	**3 (0.4)**	**2 (0.8)**	**1 (0.2)**
Synthetic anticholinergics, quaternary ammonium compounds	1 (0.1)	1 (0.4)	0 (0)
glycopyrronium bromide	1 (0.1)	1 (0.4)	0 (0)
Other drugs for functional gastrointestinal disorders	1 (0.1)	0 (0)	1 (0.2)
phloroglucinol	1 (0.1)	0 (0)	1 (0.2)
Propulsives	1 (0.1)	1 (0.4)	0 (0)
metoclopramide	1 (0.1)	1 (0.4)	0 (0)
**ANTIEMETICS AND ANTINAUSEANTS**	**3 (0.4)**	**1 (0.4)**	**2 (0.5)**
Serotonin (5HT3) antagonists	3 (0.4)	1 (0.4)	2 (0.5)
ondansetron	2 (0.3)	1 (0.4)	1 (0.2)
granisetron	1 (0.1)	0 (0)	1 (0.2)
**BILE AND LIVER THERAPY**	**1 (0.1)**	**0 (0)**	**1 (0.2)**
Bile acid preparations	1 (0.1)	0 (0)	1 (0.2)
ursodeoxycholic acid	1 (0.1)	0 (0)	1 (0.2)
**DIGESTIVES, INCL. ENZYMES**	**1 (0.1)**	**0 (0)**	**1 (0.2)**
Enzyme preparations	1 (0.1)	0 (0)	1 (0.2)
**DRUGS USED IN DIABETES**	**13 (1.8)**	**3 (1.1)**	**10 (2.3)**
INSULINS AND ANALOGUES	6 (0.8)	2 (0.8)	4 (0.9)

Insulins and analogues for injection, fast-acting	1 (0.1)	0 (0)	1 (0.2)
insulin aspart	1 (0.1)	0 (0)	1 (0.2)
Insulins and analogues for injection, long-acting	1 (0.1)	0 (0)	1 (0.2)
insulin detemir	1 (0.1)	0 (0)	1 (0.2)
**BLOOD GLUCOSE LOWERING DRUGS, EXCL. INSULINS**	**7 (1)**	**1 (0.4)**	**6 (1.4)**
Biguanides	3 (0.4)	1 (0.4)	2 (0.5)
metformin	3 (0.4)	1 (0.4)	2 (0.5)
Sulfonamides, urea derivatives	3 (0.4)	0 (0)	3 (0.7)
glibenclamide	1 (0.1)	0 (0)	1 (0.2)
gliclazide	1 (0.1)	0 (0)	1 (0.2)
glimepiride	1 (0.1)	0 (0)	1 (0.2)
Alpha glucosidase inhibitors	1 (0.1)	0 (0)	1 (0.2)
acarbose	1 (0.1)	0 (0)	1 (0.2)
**VITAMINS**	**6 (0.8)**	**1 (0.4)**	**5 (1.1)**
Vitamin D and analogues	2 (0.3)	0 (0)	2 (0.5)
colecalciferol	1 (0.1)	0 (0)	1 (0.2)
Vitamin B-complex, plain	1 (0.1)	0 (0)	1 (0.2)
Ascorbic acid (vitamin C), plain	3 (0.4)	1 (0.4)	2 (0.5)
ascorbic acid (vit C)	3 (0.4)	1 (0.4)	2 (0.5)
**MINERAL SUPPLEMENTS**	**3 (0.4)**	**0 (0)**	**3 (0.7)**
Calcium	2 (0.3)	0 (0)	2 (0.5)
calcium carbonate	1 (0.1)	0 (0)	1 (0.2)
calcium citrate	1 (0.1)	0 (0)	1 (0.2)
**OTHER ALIMENTARY TRACT AND METABOLISM PRODUCTS**	**2 (0.3)**	**0 (0)**	**2 (0.5)**
Various alimentary tract and metabolism products	2 (0.3)	0 (0)	2 (0.5)
anethole trithione	2 (0.3)	0 (0)	2 (0.5)
**BLOOD AND BLOOD FORMING ORGANS**	**34 (4.7)**	**17 (6.4)**	**17 (3.8)**
**ANTITHROMBOTIC AGENTS**	**28 (3.9)**	**14 (5.3)**	**14 (3.2)**
Vitamin K antagonists	2 (0.3)	2 (0.8)	0 (0)
warfarin	1 (0.1)	1 (0.4)	0 (0)
acenocoumarol	1 (0.1)	1 (0.4)	0 (0)
Heparin group	12 (1.7)	4 (1.5)	8 (1.8)
heparin	3 (0.4)	0 (0)	3 (0.7)
enoxaparin	7 (1)	3 (1.1)	4 (0.9)
nadroparin	1 (0.1)	1 (0.4)	0 (0)
bemiparin	1 (0.1)	0 (0)	1 (0.2)
Platelet aggregation inhibitors excl. heparin	11 (1.5)	7 (2.6)	4 (0.9)
clopidogrel	2 (0.3)	1 (0.4)	1 (0.2)
acetylsalicylic acid	10 (1.4)	7 (2.6)	3 (0.7)
Direct thrombin inhibitors	1 (0.1)	1 (0.4)	0 (0)
dabigatran etexilate	1 (0.1)	1 (0.4)	0 (0)
Direct factor Xa inhibitors	2 (0.3)	0 (0)	2 (0.5)

apixaban	2 (0.3)	0 (0)	2 (0.5)
**ANTIANEMIC PREPARATIONS**	**5 (0.7)**	**2 (0.8)**	**3 (0.7)**
IRON PREPARATIONS	3 (0.4)	1 (0.4)	2 (0.5)
Iron bivalent, oral preparations	2 (0.3)	0 (0)	2 (0.5)
ferrous sulfate	2 (0.3)	0 (0)	2 (0.5)
Iron, parenteral preparations	1 (0.1)	1 (0.4)	0 (0)
VITAMIN B12 AND FOLIC ACID	1 (0.1)	1 (0.4)	0 (0)
Folic acid and derivatives	1 (0.1)	1 (0.4)	0 (0)
folic acid	1 (0.1)	1 (0.4)	0 (0)
OTHER ANTIANEMIC PREPARATIONS	1 (0.1)	0 (0)	1 (0.2)
Other antianemic preparations	1 (0.1)	0 (0)	1 (0.2)
**BLOOD SUBSTITUTES AND PERFUSION SOLUTIONS**	**1 (0.1)**	**1 (0.4)**	**0 (0)**
Solutions producing osmotic diuresis	1 (0.1)	1 (0.4)	0 (0)
mannitol	1 (0.1)	1 (0.4)	0 (0)
**CARDIOVASCULAR SYSTEM**	**66 (9.2)**	**22 (8.3)**	**41 (9.3)**
**CARDIAC THERAPY**	**10 (1.4)**	**5 (1.9)**	**5 (1.1)**
CARDIAC GLYCOSIDES	1 (0.1)	0 (0)	1 (0.2)
Digitalis glycosides	1 (0.1)	0 (0)	1 (0.2)
digoxin	1 (0.1)	0 (0)	1 (0.2)
ANTIARRHYTHMICS, CLASS I AND III	4 (0.6)	2 (0.8)	2 (0.5)
Antiarrhythmics, class Ia	1 (0.1)	1 (0.4)	0 (0)
procainamide	1 (0.1)	1 (0.4)	0 (0)
Antiarrhythmics, class III	3 (0.4)	1 (0.4)	2 (0.5)
amiodarone	3 (0.4)	1 (0.4)	2 (0.5)
CARDIAC STIMULANTS EXCL. CARDIAC GLYCOSIDES	2 (0.3)	2 (0.8)	0 (0)
Adrenergic and dopaminergic agents	2 (0.3)	2 (0.8)	0 (0)
dobutamine	1 (0.1)	1 (0.4)	0 (0)
ephedrine	1 (0.1)	1 (0.4)	0 (0)
VASODILATORS USED IN CARDIAC DISEASES	3 (0.4)	1 (0.4)	2 (0.5)
Organic nitrates	3 (0.4)	1 (0.4)	2 (0.5)
isosorbide dinitrate	2 (0.3)	1 (0.4)	1 (0.2)
isosorbide mononitrate	1 (0.1)	0 (0)	1 (0.2)
Alpha-adrenoreceptor antagonists	1 (0.1)	1 (0.4)	0 (0)
**DIURETICS**	**15 (2.1)**	**5 (1.9)**	**9 (2)**
LOW-CEILING DIURETICS, THIAZIDES	5 (0.7)	1 (0.4)	4 (0.9)
Thiazides, plain	5 (0.7)	1 (0.4)	4 (0.9)
hydrochlorothiazide	5 (0.7)	1 (0.4)	4 (0.9)
LOW-CEILING DIURETICS, EXCL. THIAZIDES	2 (0.3)	0 (0)	2 (0.5)
Sulfonamides, plain	7 (1)	3 (1.1)	3 (0.7)
indapamide	2 (0.3)	0 (0)	2 (0.5)
Xanthine derivatives	1 (0.1)	0 (0)	1 (0.2)
HIGH-CEILING DIURETICS	5 (0.7)	3 (1.1)	1 (0.2)

furosemide	5 (0.7)	3 (1.1)	1 (0.2)
POTASSIUM-SPARING AGENTS	3 (0.4)	1 (0.4)	2 (0.5)
Aldosterone antagonists	2 (0.3)	1 (0.4)	1 (0.2)
spironolactone	2 (0.3)	1 (0.4)	1 (0.2)
Other potassium-sparing agents	1 (0.1)	0 (0)	1 (0.2)
triamterene	1 (0.1)	0 (0)	1 (0.2)
PERIPHERAL VASODILATORS	1 (0.1)	0 (0)	1 (0.2)
PERIPHERAL VASODILATORS	1 (0.1)	0 (0)	1 (0.2)
Purine derivatives	1 (0.1)	0 (0)	1 (0.2)
pentoxifylline	1 (0.1)	0 (0)	1 (0.2)
**BETA BLOCKING AGENTS**	**8 (1.1)**	**2 (0.8)**	**5 (1.1)**
Beta blocking agents, non-selective	3 (0.4)	1 (0.4)	2 (0.5)
propranolol	2 (0.3)	1 (0.4)	1 (0.2)
nadolol	1 (0.1)	0 (0)	1 (0.2)
Beta blocking agents, selective	3 (0.4)	0 (0)	3 (0.7)
atenolol	1 (0.1)	0 (0)	1 (0.2)
bisoprolol	2 (0.3)	0 (0)	2 (0.5)
Alpha and beta blocking agents	2 (0.3)	1 (0.4)	0 (0)
labetalol	1 (0.1)	0 (0)	0 (0)
carvedilol	1 (0.1)	1 (0.4)	0 (0)
**CALCIUM CHANNEL BLOCKERS**	**10 (1.4)**	**4 (1.5)**	**6 (1.4)**
SELECTIVE CALCIUM CHANNEL BLOCKERS WITH MAINLY VASCULAR EFFECTS	5 (0.7)	1 (0.4)	4 (0.9)
Dihydropyridine derivatives	5 (0.7)	1 (0.4)	4 (0.9)
nifedipine	3 (0.4)	0 (0)	3 (0.7)
nimodipine	2 (0.3)	1 (0.4)	1 (0.2)
SELECTIVE CALCIUM CHANNEL BLOCKERS WITH DIRECT CARDIAC EFFECTS	5 (0.7)	3 (1.1)	2 (0.5)
Phenylalkylamine derivatives	1 (0.1)	1 (0.4)	0 (0)
verapamil	1 (0.1)	1 (0.4)	0 (0)
Benzothiazepine derivatives	4 (0.6)	2 (0.8)	2 (0.5)
diltiazem	4 (0.6)	2 (0.8)	2 (0.5)
**AGENTS ACTING ON THE RENIN-ANGIOTENSIN SYSTEM**	**11 (1.5)**	**3 (1.1)**	**8 (1.8)**
ACE INHIBITORS, PLAIN	8 (1.1)	3 (1.1)	5 (1.1)
ACE inhibitors, plain	8 (1.1)	3 (1.1)	5 (1.1)
captopril	2 (0.3)	1 (0.4)	1 (0.2)
enalapril	3 (0.4)	0 (0)	3 (0.7)
lisinopril	2 (0.3)	1 (0.4)	1 (0.2)
ramipril	1 (0.1)	1 (0.4)	0 (0)
ANGIOTENSIN II ANTAGONISTS, PLAIN	2 (0.3)	0 (0)	2 (0.5)
Angiotensin II antagonists, plain	2 (0.3)	0 (0)	2 (0.5)
losartan	1 (0.1)	0 (0)	1 (0.2)
telmisartan	1 (0.1)	0 (0)	1 (0.2)
ANGIOTENSIN II ANTAGONISTS, COMBINATIONS	1 (0.1)	0 (0)	1 (0.2)

Angiotensin II antagonists, other combinations	1 (0.1)	0 (0)	1 (0.2)
olmesartan medoxomil, amlodipine and hydrochlorothiazide	1 (0.1)	0 (0)	1 (0.2)
**LIPID MODIFYING AGENTS**	**11 (1.5)**	**3 (1.1)**	**7 (1.6)**
LIPID MODIFYING AGENTS, PLAIN	11 (1.5)	3 (1.1)	7 (1.6)
HMG CoA reductase inhibitors	7 (1)	2 (0.8)	4 (0.9)
simvastatin	5 (0.7)	1 (0.4)	3 (0.7)
pravastatin	1 (0.1)	0 (0)	1 (0.2)
atorvastatin	1 (0.1)	1 (0.4)	0 (0)
Fibrates	3 (0.4)	0 (0)	3 (0.7)
gemfibrozil	2 (0.3)	0 (0)	2 (0.5)
fenofibrate	1 (0.1)	0 (0)	1 (0.2)
Bile acid sequestrants	1 (0.1)	1 (0.4)	0 (0)
colestyramine	1 (0.1)	1 (0.4)	0 (0)
**DERMATOLOGICALS**	**21 (2.9)**	**9 (3.4)**	**12 (2.7)**
ANTIFUNGALS FOR DERMATOLOGICAL USE	12 (1.5)	4 (1.5)	8 (1.8)
ANTIFUNGALS FOR TOPICAL USE	3 (0.4)	0 (0)	3 (0.7)
nystatin	3 (0.4)	0 (0)	3 (0.7)
ANTIFUNGALS FOR SYSTEMIC USE	9 (1.3)	4 (1.5)	5 (1.1)
Antifungals for systemic use	9 (1.3)	4 (1.5)	5 (1.1)
terbinafine	9 (1.3)	4 (1.5)	5 (1.1)
**ANTIPSORIATICS**	**4 (0.6)**	**3 (1.1)**	**1 (0.2)**
ANTIPSORIATICS FOR SYSTEMIC USE	4 (0.6)	3 (1.1)	1 (0.2)
Psoralens for systemic use	1 (0.1)	1 (0.4)	0 (0)
Retinoids for treatment of psoriasis	3 (0.4)	2 (0.8)	1 (0.2)
acitretin	3 (0.4)	2 (0.8)	1 (0.2)
**ANTIBIOTICS AND CHEMOTHERAPEUTICS FOR DERMATOLOGICAL USE**	**2 (0.3)**	**0 (0)**	**2 (0.5)**
CHEMOTHERAPEUTICS FOR TOPICAL USE	2 (0.3)	0 (0)	2 (0.5)
Antivirals	1 (0.1)	0 (0)	1 (0.2)
Other chemotherapeutics	1 (0.1)	0 (0)	1 (0.2)
**CORTICOSTEROIDS, DERMATOLOGICAL PREPARATIONS**	**4 (0.6)**	**2 (0.8)**	**2 (0.5)**
**GENITO URINARY SYSTEM AND SEX HORMONES**	**8 (1.1)**	**3 (1.1)**	**5 (1.1)**
**GYNECOLOGICAL ANTIINFECTIVES AND ANTISEPTICS**	**4 (0.6)**	**2 (0.8)**	**2 (0.5)**
Triazole derivatives	4 (0.6)	2 (0.8)	2 (0.5)
**OTHER GYNECOLOGICALS**	**1 (0.1)**	**0 (0)**	**1 (0.2)**
Sympathomimetics, labour repressants	1 (0.1)	0 (0)	1 (0.2)
ritodrine	1 (0.1)	0 (0)	1 (0.2)
**SEX HORMONES AND MODULATORS OF THE GENITAL SYSTEM**	**4 (0.6)**	**0 (0)**	**4 (0.9)**
ESTROGENS	2 (0.3)	0 (0)	2 (0.5)
Natural and semisynthetic estrogens, plain	2 (0.3)	0 (0)	2 (0.5)
estradiol	1 (0.1)	0 (0)	1 (0.2)
PROGESTOGENS	1 (0.1)	0 (0)	1 (0.2)
Pregnen (4) derivatives	1 (0.1)	0 (0)	1 (0.2)

progesterone	1 (0.1)	0 (0)	1 (0.2)
OTHER SEX HORMONES AND MODULATORS OF THE GENITAL SYSTEM	1 (0.1)	0 (0)	1 (0.2)
Progesterone receptor modulators	1 (0.1)	0 (0)	1 (0.2)
mifepristone	1 (0.1)	0 (0)	1 (0.2)
**UROLOGICALS**	**3 (0.4)**	**3 (1.1)**	**0 (0)**
DRUGS USED IN BENIGN PROSTATIC HYPERTROPHY	3 (0.4)	3 (1.1)	0 (0)
terazosin	1 (0.1)	1 (0.4)	0 (0)
finasteride	2 (0.3)	2 (0.8)	0 (0)
**SYSTEMIC HORMONAL PREPARATIONS, EXCL. SEX HORMONES AND INSULINS**	**35 (4.9)**	**10 (3.8)**	**25 (5.7)**
**PITUITARY AND HYPOTHALAMIC HORMONES AND ANALOGUES**	**1 (0.1)**	**1 (0.4)**	**0 (0)**
Somatropin and somatropin agonists	1 (0.1)	1 (0.4)	0 (0)
somatropin	1 (0.1)	1 (0.4)	0 (0)
**CORTICOSTEROIDS FOR SYSTEMIC USE**	**31 (4.3)**	**8 (3)**	**23 (5.2)**
Glucocorticoids	31 (4.3)	8 (3)	23 (5.2)
betamethasone	1 (0.1)	0 (0)	1 (0.2)
methylprednisolone	2 (0.3)	1 (0.4)	1 (0.2)
prednisolone	13 (1.8)	3 (1.1)	10 (2.3)
prednisone	9 (1.3)	1 (0.4)	8 (1.8)
hydrocortisone	1 (0.1)	1 (0.4)	0 (0)
deflazacort	1 (0.1)	0 (0)	1 (0.2)
**THYROID THERAPY**	**3 (0.4)**	**1 (0.4)**	**2 (0.5)**
Thyroid hormones	3 (0.4)	1 (0.4)	2 (0.5)
levothyroxine sodium	2 (0.3)	1 (0.4)	1 (0.2)
**ANTIINFECTIVES FOR SYSTEMIC USE**	**184 (25.7)**	**76 (28.7)**	**103 (23.3)**
**ANTIBACTERIALS FOR SYSTEMIC USE**	**154 (21.5)**	**62 (23.4)**	**90 (20.4)**
TETRACYCLINES	4 (0.6)	2 (0.8)	2 (0.5)
Tetracyclines	4 (0.6)	2 (0.8)	2 (0.5)
doxycycline	2 (0.3)	0 (0)	2 (0.5)
tetracycline	1 (0.1)	1 (0.4)	0 (0)
tigecycline	1 (0.1)	1 (0.4)	0 (0)
BETA-LACTAM ANTIBACTERIALS, PENICILLINS	45 (6.3)	16 (6)	29 (6.6)
Penicillins with extended spectrum	13 (1.8)	3 (1.1)	10 (2.3)
amoxicillin	10 (1.4)	3 (1.1)	7 (1.6)
piperacillin	1 (0.1)	0 (0)	1 (0.2)
ampicillin, combinations	2 (0.3)	0 (0)	2 (0.5)
Beta-lactamase sensitive penicillins	1 (0.1)	1 (0.4)	0 (0)
benzylpenicillin	1 (0.1)	1 (0.4)	0 (0)
Beta-lactamase resistant penicillins	6 (0.8)	3 (1.1)	3 (0.7)
dicloxacillin	1 (0.1)	1 (0.4)	0 (0)
cloxacillin	1 (0.1)	1 (0.4)	0 (0)
oxacillin	1 (0.1)	0 (0)	1 (0.2)
flucloxacillin	3 (0.4)	1 (0.4)	2 (0.5)

Beta-lactamase inhibitors	1 (0.1)	0 (0)	1 (0.2)
sulbactam	1 (0.1)	0 (0)	1 (0.2)
Combinations of penicillins, incl. beta-lactamase inhibitors	24 (3.3)	9 (3.4)	15 (3.4)
ampicillin and enzyme inhibitor	1 (0.1)	1 (0.4)	0 (0)
amoxicillin and enzyme inhibitor	18 (2.5)	6 (2.3)	12 (2.7)
piperacillin and enzyme inhibitor	5 (0.7)	2 (0.8)	3 (0.7)
OTHER BETA-LACTAM ANTIBACTERIALS	36 (5)	15 (5.7)	20 (4.5)
First-generation cephalosporins	7 (1)	2 (0.8)	5 (1.1)
cefalexin	3 (0.4)	0 (0)	3 (0.7)
cefazolin	4 (0.6)	2 (0.8)	2 (0.5)
Second-generation cephalosporins	2 (0.3)	1 (0.4)	1 (0.2)
cefprozil	1 (0.1)	1 (0.4)	0 (0)
flomoxef	1 (0.1)	0 (0)	1 (0.2)
Third-generation cephalosporins	18 (2.5)	8 (3)	9 (2)
cefotaxime	1 (0.1)	0 (0)	1 (0.2)
ceftazidime	1 (0.1)	1 (0.4)	0 (0)
ceftriaxone	12 (1.7)	6 (2.3)	5 (1.1)
cefoperazone	1 (0.1)	0 (0)	1 (0.2)
cefdinir	1 (0.1)	0 (0)	1 (0.2)
cefditoren	1 (0.1)	0 (0)	1 (0.2)
ceftriaxone, combinations	1 (0.1)	1 (0.4)	0 (0)
Fourth-generation cephalosporins	3 (0.4)	2 (0.8)	1 (0.2)
cefepime	3 (0.4)	2 (0.8)	1 (0.2)
Carbapenems	5 (0.7)	2 (0.8)	3 (0.7)
meropenem	4 (0.6)	2 (0.8)	2 (0.5)
ertapenem	1 (0.1)	0 (0)	1 (0.2)
SULFONAMIDES AND TRIMETHOPRIM	9 (1.3)	7 (2.6)	2 (0.5)
Combinations of sulfonamides and trimethoprim, incl. derivatives	9 (1.3)	7 (2.6)	2 (0.5)
sulfamethoxazole and trimethoprim	9 (1.3)	7 (2.6)	2 (0.5)
MACROLIDES, LINCOSAMIDES AND STREPTOGRAMINS	28 (3.9)	8 (3)	20 (4.5)
Macrolides	12 (1.7)	5 (1.9)	7 (1.6)
erythromycin	2 (0.3)	1 (0.4)	1 (0.2)
josamycin	1 (0.1)	1 (0.4)	0 (0)
clarithromycin	1 (0.1)	0 (0)	1 (0.2)
azithromycin	8 (1.1)	3 (1.1)	5 (1.1)
Lincosamides	14 (2)	3 (1.1)	11 (2.5)
clindamycin	13 (1.8)	3 (1.1)	10 (2.3)
lincomycin	1 (0.1)	0 (0)	1 (0.2)
Streptogramins	2 (0.3)	0 (0)	2 (0.5)
pristinamycin	2 (0.3)	0 (0)	2 (0.5)
AMINOGLYCOSIDE ANTIBACTERIALS	1 (0.1)	0 (0)	1 (0.2)
Other aminoglycosides	1 (0.1)	0 (0)	1 (0.2)

gentamicin	1 (0.1)	0 (0)	1 (0.2)
QUINOLONE ANTIBACTERIALS	8 (1.1)	4 (1.5)	4 (0.9)
Fluoroquinolones	8 (1.1)	4 (1.5)	4 (0.9)
ciprofloxacin	5 (0.7)	3 (1.1)	2 (0.5)
levofloxacin	2 (0.3)	1 (0.4)	1 (0.2)
tosufloxacin	1 (0.1)	0 (0)	1 (0.2)
OTHER ANTIBACTERIALS	23 (3.2)	10 (3.8)	12 (2.7)
Glycopeptide antibacterials	16 (2.2)	5 (1.9)	10 (2.3)
vancomycin	13 (1.8)	5 (1.9)	7 (1.6)
teicoplanin	2 (0.3)	0 (0)	2 (0.5)
telavancin	1 (0.1)	0 (0)	1 (0.2)
metronidazole	4 (0.6)	2 (0.8)	2 (0.5)
Other antibacterials	4 (0.6)	3 (1.1)	1 (0.2)
fosfomycin	1 (0.1)	0 (0)	1 (0.2)
linezolid	1 (0.1)	1 (0.4)	0 (0)
daptomycin	2 (0.3)	2 (0.8)	0 (0)
**ANTIMYCOTICS FOR SYSTEMIC USE**	**4 (0.6)**	**2 (0.8)**	**2 (0.5)**
fluconazole	3 (0.4)	1 (0.4)	2 (0.5)
voriconazole	1 (0.1)	1 (0.4)	0 (0)
**ANTIMYCOBACTERIALS**	**1 (0.1)**	**0 (0)**	**1 (0.2)**
DRUGS FOR TREATMENT OF TUBERCULOSIS	1 (0.1)	0 (0)	1 (0.2)
rifampicin	1 (0.1)	0 (0)	1 (0.2)
**ANTIVIRALS FOR SYSTEMIC USE**	**16 (2.2)**	**10 (3.8)**	**6 (1.4)**
Nucleosides and nucleotides excl. reverse transcriptase inhibitors	4 (0.6)	2 (0.8)	2 (0.5)
aciclovir	3 (0.4)	1 (0.4)	2 (0.5)
valaciclovir	2 (0.3)	1 (0.4)	1 (0.2)
Nucleoside and nucleotide reverse transcriptase inhibitors	1 (0.1)	0 (0)	1 (0.2)
entecavir	1 (0.1)	0 (0)	1 (0.2)
Neuraminidase inhibitors	2 (0.3)	2 (0.8)	0 (0)
oseltamivir	2 (0.3)	2 (0.8)	0 (0)
Antivirals for treatment of HCV infections	2 (0.3)	2 (0.8)	0 (0)
ribavirin	2 (0.3)	2 (0.8)	0 (0)
Antivirals for treatment of HIV infections, combinations	5 (0.7)	2 (0.8)	3 (0.7)
lopinavir-ritonavir,kaletra	4 (0.6)	2 (0.8)	2 (0.5)
darunavir and cobicistat	1 (0.1)	0 (0)	1 (0.2)
Other antivirals	2 (0.3)	2 (0.8)	0 (0)
favipiravir	2 (0.3)	2 (0.8)	0 (0)
**VACCINES**	**9 (1.3)**	**2 (0.8)**	**4 (0.9)**
BACTERIAL VACCINES	2 (0.3)	0 (0)	0 (0)
Pertussis vaccines	1 (0.1)	0 (0)	0 (0)
pertussis, purified antigen, combinations with toxoids	1 (0.1)	0 (0)	0 (0)
Pneumococcal vaccines	1 (0.1)	0 (0)	0 (0)

pneumococcus, purified polysaccharides antigen	1 (0.1)	0 (0)	0 (0)
VIRAL VACCINES	7 (1)	2 (0.8)	4 (0.9)
Influenza vaccines	1 (0.1)	0 (0)	1 (0.2)
Measles vaccines	1 (0.1)	0 (0)	0 (0)
measles, combinations with mumps and rubella, live attenuated	1 (0.1)	0 (0)	0 (0)
Other viral vaccines	5 (0.7)	2 (0.8)	3 (0.7)
covid-19 vaccines	5 (0.7)	2 (0.8)	3 (0.7)
**ANTINEOPLASTIC AND IMMUNOMODULATING AGENTS**	**69 (9.6)**	**31 (11.7)**	**38 (8.6)**
**ANTINEOPLASTIC AGENTS**	**45 (6.3)**	**22 (8.3)**	**23 (5.2)**
ALKYLATING AGENTS	4 (0.6)	2 (0.8)	2 (0.5)
Nitrogen mustard analogues	2 (0.3)	0 (0)	2 (0.5)
cyclophosphamide	2 (0.3)	0 (0)	2 (0.5)
Other alkylating agents	2 (0.3)	2 (0.8)	0 (0)
temozolomide	2 (0.3)	2 (0.8)	0 (0)
ANTIMETABOLITES	5 (0.7)	3 (1.1)	2 (0.5)
Pyrimidine analogues	5 (0.7)	3 (1.1)	2 (0.5)
cytarabine	1 (0.1)	1 (0.4)	0 (0)
gemcitabine	2 (0.3)	2 (0.8)	0 (0)
capecitabine	1 (0.1)	0 (0)	1 (0.2)
fluorouracil, combinations	1 (0.1)	0 (0)	1 (0.2)
PLANT ALKALOIDS AND OTHER NATURAL PRODUCTS	6 (0.8)	4 (1.5)	2 (0.5)
Podophyllotoxin derivatives	2 (0.3)	2 (0.8)	0 (0)
etoposide	2 (0.3)	2 (0.8)	0 (0)
Taxanes	4 (0.6)	2 (0.8)	2 (0.5)
paclitaxel	1 (0.1)	1 (0.4)	0 (0)
docetaxel	3 (0.4)	1 (0.4)	2 (0.5)
CYTOTOXIC ANTIBIOTICS AND RELATED SUBSTANCES	3 (0.4)	1 (0.4)	2 (0.5)
Anthracyclines and related substances	2 (0.3)	0 (0)	2 (0.5)
doxorubicin	1 (0.1)	0 (0)	1 (0.2)
epirubicin	1 (0.1)	0 (0)	1 (0.2)
Other cytotoxic antibiotics	1 (0.1)	1 (0.4)	0 (0)
bleomycin	1 (0.1)	1 (0.4)	0 (0)
imatinib	5 (0.7)	0 (0)	5 (1.1)
gefitinib	2 (0.3)	1 (0.4)	1 (0.2)
erlotinib	2 (0.3)	1 (0.4)	1 (0.2)
vemurafenib	1 (0.1)	1 (0.4)	0 (0)
lapatinib	1 (0.1)	0 (0)	1 (0.2)
sorafenib	3 (0.4)	0 (0)	3 (0.7)
OTHER ANTINEOPLASTIC AGENTS	27 (3.8)	12 (4.5)	15 (3.4)
Platinum compounds	3 (0.4)	3 (1.1)	0 (0)
cisplatin	2 (0.3)	2 (0.8)	0 (0)
carboplatin	1 (0.1)	1 (0.4)	0 (0)

Monoclonal antibodies	8 (1.1)	6 (2.3)	2 (0.5)
rituximab	2 (0.3)	0 (0)	2 (0.5)
cetuximab	1 (0.1)	1 (0.4)	0 (0)
ipilimumab	2 (0.3)	2 (0.8)	0 (0)
nivolumab	1 (0.1)	1 (0.4)	0 (0)
pembrolizumab	1 (0.1)	1 (0.4)	0 (0)
atezolizumab	1 (0.1)	1 (0.4)	0 (0)
Protein kinase inhibitors	14 (2)	3 (1.1)	11 (2.5)
vismodegib	1 (0.1)	0 (0)	1 (0.2)
Other antineoplastic agents	2 (0.3)	0 (0)	2 (0.5)
hydroxycarbamide	1 (0.1)	0 (0)	1 (0.2)
**ENDOCRINE THERAPY**	**4 (0.6)**	**4 (1.5)**	**0 (0)**
HORMONES AND RELATED AGENTS	1 (0.1)	1 (0.4)	0 (0)
Gonadotropin releasing hormone analogues	1 (0.1)	1 (0.4)	0 (0)
goserelin	1 (0.1)	1 (0.4)	0 (0)
HORMONE ANTAGONISTS AND RELATED AGENTS	3 (0.4)	3 (1.1)	0 (0)
Anti-androgens	2 (0.3)	2 (0.8)	0 (0)
bicalutamide	1 (0.1)	1 (0.4)	0 (0)
enzalutamide	1 (0.1)	1 (0.4)	0 (0)
Other hormone antagonists and related agents	1 (0.1)	1 (0.4)	0 (0)
degarelix	1 (0.1)	1 (0.4)	0 (0)
**IMMUNOSTIMULANTS**	**3 (0.4)**	**1 (0.4)**	**2 (0.5)**
Interferons	3 (0.4)	1 (0.4)	2 (0.5)
interferon alfa natural	1 (0.1)	0 (0)	1 (0.2)
interferon beta natural	1 (0.1)	1 (0.4)	0 (0)
interferon beta-1a	1 (0.1)	0 (0)	1 (0.2)
**IMMUNOSUPPRESSANTS**	**17 (2.4)**	**4 (1.5)**	**13 (2.9)**
Selective immunosuppressants	2 (0.3)	0 (0)	2 (0.5)
mycophenolic acid	1 (0.1)	0 (0)	1 (0.2)
leflunomide	1 (0.1)	0 (0)	1 (0.2)
Tumor necrosis factor alpha (TNF-α) inhibitors	3 (0.4)	1 (0.4)	2 (0.5)
etanercept	2 (0.3)	1 (0.4)	1 (0.2)
infliximab	1 (0.1)	0 (0)	1 (0.2)
Interleukin inhibitors	4 (0.6)	1 (0.4)	3 (0.7)
ustekinumab	1 (0.1)	0 (0)	1 (0.2)
tocilizumab	2 (0.3)	0 (0)	2 (0.5)
ixekizumab	1 (0.1)	1 (0.4)	0 (0)
Calcineurin inhibitors	3 (0.4)	0 (0)	3 (0.7)
ciclosporin	2 (0.3)	0 (0)	2 (0.5)
tacrolimus	1 (0.1)	0 (0)	1 (0.2)
Other immunosuppressants	5 (0.7)	2 (0.8)	3 (0.7)
azathioprine	2 (0.3)	0 (0)	2 (0.5)

methotrexate	3 (0.4)	2 (0.8)	1 (0.2)
**MUSCULO-SKELETAL SYSTEM**	**35 (4.9)**	**9 (3.4)**	**26 (5.9)**
**ANTIINFLAMMATORY AND ANTIRHEUMATIC PRODUCTS**	**28 (3.9)**	**7 (2.6)**	**21 (4.8)**
Acetic acid derivatives and related substances	6 (0.8)	2 (0.8)	4 (0.9)
diclofenac	2 (0.3)	0 (0)	2 (0.5)
etodolac	2 (0.3)	1 (0.4)	1 (0.2)
acemetacin	1 (0.1)	0 (0)	1 (0.2)
diclofenac, combinations	1 (0.1)	1 (0.4)	0 (0)
Oxicams	3 (0.4)	0 (0)	3 (0.7)
piroxicam	1 (0.1)	0 (0)	1 (0.2)
lornoxicam	1 (0.1)	0 (0)	1 (0.2)
meloxicam	1 (0.1)	0 (0)	1 (0.2)
Propionic acid derivatives	10 (1.4)	3 (1.1)	7 (1.6)
ibuprofen	6 (0.8)	3 (1.1)	3 (0.7)
ketoprofen	1 (0.1)	0 (0)	1 (0.2)
flurbiprofen	2 (0.3)	0 (0)	2 (0.5)
tiaprofenic acid	1 (0.1)	0 (0)	1 (0.2)
Coxibs	7 (1)	2 (0.8)	5 (1.1)
celecoxib	5 (0.7)	1 (0.4)	4 (0.9)
valdecoxib	1 (0.1)	0 (0)	1 (0.2)
etoricoxib	1 (0.1)	1 (0.4)	0 (0)
Other antiinflammatory and antirheumatic agents, non-steroids	2 (0.3)	0 (0)	2 (0.5)
nimesulide	1 (0.1)	0 (0)	1 (0.2)
chondroitin sulfate	1 (0.1)	0 (0)	1 (0.2)
**MUSCLE RELAXANTS**	**3 (0.4)**	**1 (0.4)**	**2 (0.5)**
MUSCLE RELAXANTS, PERIPHERALLY ACTING AGENTS	1 (0.1)	1 (0.4)	0 (0)
Other quaternary ammonium compounds	1 (0.1)	1 (0.4)	0 (0)
pancuronium	1 (0.1)	1 (0.4)	0 (0)
MUSCLE RELAXANTS, CENTRALLY ACTING AGENTS	2 (0.3)	0 (0)	2 (0.5)
Carbamic acid esters	1 (0.1)	0 (0)	1 (0.2)
methocarbamol	1 (0.1)	0 (0)	1 (0.2)
Other centrally acting agents	1 (0.1)	0 (0)	1 (0.2)
eperisone	1 (0.1)	0 (0)	1 (0.2)
**ANTIGOUT PREPARATIONS**	**3 (0.4)**	**1 (0.4)**	**2 (0.5)**
Preparations inhibiting uric acid production	1 (0.1)	1 (0.4)	0 (0)
allopurinol	1 (0.1)	1 (0.4)	0 (0)
Preparations with no effect on uric acid metabolism	2 (0.3)	0 (0)	2 (0.5)
colchicine	2 (0.3)	0 (0)	2 (0.5)
**DRUGS FOR TREATMENT OF BONE DISEASES**	**1 (0.1)**	**0 (0)**	**1 (0.2)**
Bisphosphonates	1 (0.1)	0 (0)	1 (0.2)
alendronic acid	1 (0.1)	0 (0)	1 (0.2)
**NERVOUS SYSTEM**	**87 (12.1)**	**41 (15.5)**	**45 (10.2)**

**ANESTHETICS**	**13 (1.8)**	**10 (3.8)**	**3 (0.7)**
ANESTHETICS, GENERAL	5 (0.7)	5 (1.9)	0 (0)
Halogenated hydrocarbons	3 (0.4)	3 (1.1)	0 (0)
halothane	1 (0.1)	1 (0.4)	0 (0)
isoflurane	1 (0.1)	1 (0.4)	0 (0)
sevoflurane	1 (0.1)	1 (0.4)	0 (0)
Barbiturates, plain	1 (0.1)	1 (0.4)	0 (0)
thiopental	1 (0.1)	1 (0.4)	0 (0)
Other general anesthetics	1 (0.1)	1 (0.4)	0 (0)
propofol	1 (0.1)	1 (0.4)	0 (0)
ANESTHETICS, LOCAL	8 (1.1)	5 (1.9)	3 (0.7)
Amides	8 (1.1)	5 (1.9)	3 (0.7)
bupivacaine	1 (0.1)	1 (0.4)	0 (0)
lidocaine	1 (0.1)	1 (0.4)	0 (0)
mepivacaine	2 (0.3)	1 (0.4)	1 (0.2)
cinchocaine	1 (0.1)	0 (0)	1 (0.2)
bupivacaine, combinations	1 (0.1)	1 (0.4)	0 (0)
lidocaine, combinations	2 (0.3)	1 (0.4)	1 (0.2)
**ANALGESICS**	**32 (4.5)**	**13 (4.9)**	**18 (4.1)**
OPIOIDS	10 (1.4)	3 (1.1)	7 (1.6)
Natural opium alkaloids	4 (0.6)	1 (0.4)	3 (0.7)
morphine	1 (0.1)	0 (0)	1 (0.2)
oxycodone	1 (0.1)	0 (0)	1 (0.2)
dihydrocodeine	1 (0.1)	0 (0)	1 (0.2)
morphine, combinations	1 (0.1)	1 (0.4)	0 (0)
Phenylpiperidine derivatives	2 (0.3)	1 (0.4)	1 (0.2)
fentanyl	2 (0.3)	1 (0.4)	1 (0.2)
Opioids in combination with non-opioid analgesics	1 (0.1)	0 (0)	1 (0.2)
tramadol and paracetamol	1 (0.1)	0 (0)	1 (0.2)
Other opioids	3 (0.4)	1 (0.4)	2 (0.5)
tramadol	3 (0.4)	1 (0.4)	2 (0.5)
OTHER ANALGESICS AND ANTIPYRETICS	21 (2.9)	10 (3.8)	10 (2.3)
Salicylic acid and derivatives	1 (0.1)	1 (0.4)	0 (0)
Pyrazolones	3 (0.4)	1 (0.4)	2 (0.5)
metamizole sodium	3 (0.4)	1 (0.4)	2 (0.5)
Anilides	17 (2.4)	8 (3)	8 (1.8)
paracetamol	17 (2.4)	8 (3)	8 (1.8)
ANTIMIGRAINE PREPARATIONS	1 (0.1)	0 (0)	1 (0.2)
Selective serotonin (5HT1) agonists	1 (0.1)	0 (0)	1 (0.2)
sumatriptan	1 (0.1)	0 (0)	1 (0.2)
**ANTIEPILEPTICS**	**14 (2)**	**6 (2.3)**	**8 (1.8)**
ANTIEPILEPTICS	14 (2)	6 (2.3)	8 (1.8)

Hydantoin derivatives	2 (0.3)	1 (0.4)	1 (0.2)
phenytoin	2 (0.3)	1 (0.4)	1 (0.2)
Benzodiazepine derivatives	6 (0.8)	2 (0.8)	4 (0.9)
clonazepam	1 (0.1)	1 (0.4)	0 (0)
Carboxamide derivatives	1 (0.1)	1 (0.4)	0 (0)
carbamazepine	1 (0.1)	1 (0.4)	0 (0)
Fatty acid derivatives	2 (0.3)	0 (0)	2 (0.5)
valproic acid	2 (0.3)	0 (0)	2 (0.5)
Other antiepileptics	8 (1.1)	3 (1.1)	5 (1.1)
lamotrigine	2 (0.3)	0 (0)	2 (0.5)
gabapentin	2 (0.3)	0 (0)	2 (0.5)
levetiracetam	3 (0.4)	2 (0.8)	1 (0.2)
cannabidiol	1 (0.1)	1 (0.4)	0 (0)
**ANTI-PARKINSON DRUGS**	**2 (0.3)**	**2 (0.8)**	**0 (0)**
DOPAMINERGIC AGENTS	2 (0.3)	2 (0.8)	0 (0)
Dopa and dopa derivatives	1 (0.1)	1 (0.4)	0 (0)
levodopa and decarboxylase inhibitor	1 (0.1)	1 (0.4)	0 (0)
Dopamine agonists	1 (0.1)	1 (0.4)	0 (0)
pramipexole	1 (0.1)	1 (0.4)	0 (0)
**PSYCHOLEPTICS**	**12 (1.7)**	**5 (1.9)**	**7 (1.6)**
ANTIPSYCHOTICS	3 (0.4)	1 (0.4)	2 (0.5)
Phenothiazines with piperazine structure	1 (0.1)	0 (0)	1 (0.2)
prochlorperazine	1 (0.1)	0 (0)	1 (0.2)
Diazepines, oxazepines, thiazepines and oxepines	2 (0.3)	1 (0.4)	1 (0.2)
olanzapine	2 (0.3)	1 (0.4)	1 (0.2)
ANXIOLYTICS	7 (1)	2 (0.8)	5 (1.1)
chlordiazepoxide	1 (0.1)	0 (0)	1 (0.2)
lorazepam	1 (0.1)	0 (0)	1 (0.2)
alprazolam	2 (0.3)	0 (0)	2 (0.5)
Diphenylmethane derivatives	3 (0.4)	2 (0.8)	1 (0.2)
hydroxyzine	3 (0.4)	2 (0.8)	1 (0.2)
HYPNOTICS AND SEDATIVES	2 (0.3)	2 (0.8)	0 (0)
midazolam	1 (0.1)	1 (0.4)	0 (0)
Benzodiazepine related drugs	1 (0.1)	1 (0.4)	0 (0)
zopiclone	1 (0.1)	1 (0.4)	0 (0)
**PSYCHOANALEPTICS**	**11 (1.5)**	**3 (1.1)**	**8 (1.8)**
ANTIDEPRESSANTS	9 (1.3)	2 (0.8)	7 (1.6)
Non-selective monoamine reuptake inhibitors	1 (0.1)	0 (0)	1 (0.2)
doxepin	1 (0.1)	0 (0)	1 (0.2)
Selective serotonin reuptake inhibitors	4 (0.6)	2 (0.8)	2 (0.5)
paroxetine	2 (0.3)	1 (0.4)	1 (0.2)
sertraline	2 (0.3)	1 (0.4)	1 (0.2)

Other antidepressants	4 (0.6)	0 (0)	4 (0.9)
trazodone	1 (0.1)	0 (0)	1 (0.2)
bupropion	1 (0.1)	0 (0)	1 (0.2)
venlafaxine	1 (0.1)	0 (0)	1 (0.2)
duloxetine	1 (0.1)	0 (0)	1 (0.2)
PSYCHOSTIMULANTS, AGENTS USED FOR ADHD AND NOOTROPICS	2 (0.3)	1 (0.4)	1 (0.2)
Centrally acting sympathomimetics	1 (0.1)	1 (0.4)	0 (0)
methylphenidate	1 (0.1)	1 (0.4)	0 (0)
caffeine	1 (0.1)	0 (0)	1 (0.2)
Anticholinesterases	1 (0.1)	1 (0.4)	0 (0)
**OTHER NERVOUS SYSTEM DRUGS**	**3 (0.4)**	**2 (0.8)**	**1 (0.2)**
PARASYMPATHOMIMETICS	1 (0.1)	1 (0.4)	0 (0)
neostigmine	1 (0.1)	1 (0.4)	0 (0)
DRUGS USED IN ADDICTIVE DISORDERS	2 (0.3)	1 (0.4)	1 (0.2)
Drugs used in nicotine dependence	2 (0.3)	1 (0.4)	1 (0.2)
varenicline	2 (0.3)	1 (0.4)	1 (0.2)
**ANTIPARASITIC PRODUCTS, INSECTICIDES AND REPELLENTS**	**55 (7.7)**	**12 (4.5)**	**43 (9.7)**
**ANTIPROTOZOALS**	**53 (7.4)**	**10 (3.8)**	**43 (9.7)**
AGENTS AGAINST AMOEBIASIS AND OTHER PROTOZOAL DISEASES	1 (0.1)	1 (0.4)	0 (0)
Nitroimidazole derivatives	1 (0.1)	1 (0.4)	0 (0)
Other agents against amoebiasis and other protozoal diseases	1 (0.1)	1 (0.4)	0 (0)
atovaquone	1 (0.1)	1 (0.4)	0 (0)
ANTIMALARIALS	51 (7.1)	8 (3)	43 (9.7)
Aminoquinolines	49 (6.8)	8 (3)	41 (9.3)
chloroquine	2 (0.3)	1 (0.4)	1 (0.2)
hydroxychloroquine	47 (6.6)	7 (2.6)	40 (9)
Biguanide	1 (0.1)	0 (0)	1 (0.2)
proguanil, combinations	1 (0.1)	0 (0)	1 (0.2)
Methanolquinolines	1 (0.1)	0 (0)	1 (0.2)
quinine	1 (0.1)	0 (0)	1 (0.2)
AGENTS AGAINST LEISHMANIASIS AND TRYPANOSOMIASIS	1 (0.1)	1 (0.4)	0 (0)
benznidazole	1 (0.1)	1 (0.4)	0 (0)
**ANTHELMINTICS**	**1 (0.1)**	**1 (0.4)**	**0 (0)**
ANTITREMATODALS	1 (0.1)	1 (0.4)	0 (0)
Quinoline derivatives and related substances	1 (0.1)	1 (0.4)	0 (0)
praziquantel	1 (0.1)	1 (0.4)	0 (0)
ECTOPARASITICIDES, INCL. SCABICIDES, INSECTICIDES AND REPELLENTS	1 (0.1)	1 (0.4)	0 (0)
ECTOPARASITICIDES, INCL. SCABICIDES	1 (0.1)	1 (0.4)	0 (0)
Chlorine containing products	1 (0.1)	1 (0.4)	0 (0)
lindane	1 (0.1)	1 (0.4)	0 (0)
**RESPIRATORY SYSTEM**	**23 (3.2)**	**8 (3)**	**15 (3.4)**
**NASAL PREPARATIONS**	**5 (0.7)**	**2 (0.8)**	**3 (0.7)**

NASAL DECONGESTANTS FOR SYSTEMIC USE	5 (0.7)	2 (0.8)	3 (0.7)
Sympathomimetics	5 (0.7)	2 (0.8)	3 (0.7)
phenylpropanolamine	1 (0.1)	0 (0)	1 (0.2)
pseudoephedrine	2 (0.3)	1 (0.4)	1 (0.2)
pseudoephedrine, combinations	2 (0.3)	1 (0.4)	1 (0.2)
**DRUGS FOR OBSTRUCTIVE AIRWAY DISEASES**	**2 (0.3)**	**0 (0)**	**2 (0.5)**
Selective beta-2-adrenoreceptor agonists	1 (0.1)	0 (0)	1 (0.2)
ADRENERGICS FOR SYSTEMIC USE	1 (0.1)	0 (0)	1 (0.2)
salbutamol	1 (0.1)	0 (0)	1 (0.2)
OTHER SYSTEMIC DRUGS FOR OBSTRUCTIVE AIRWAY DISEASES	1 (0.1)	0 (0)	1 (0.2)
Leukotriene receptor antagonists	1 (0.1)	0 (0)	1 (0.2)
pranlukast	1 (0.1)	0 (0)	1 (0.2)
**COUGH AND COLD PREPARATIONS**	**9 (1.3)**	**3 (1.1)**	**6 (1.4)**
EXPECTORANTS, EXCL. COMBINATIONS WITH COUGH SUPPRESSANTS	5 (0.7)	1 (0.4)	4 (0.9)
Expectorants	1 (0.1)	0 (0)	1 (0.2)
guaifenesin	1 (0.1)	0 (0)	1 (0.2)
Mucolytics	4 (0.6)	1 (0.4)	3 (0.7)
carbocisteine	3 (0.4)	0 (0)	3 (0.7)
ambroxol	1 (0.1)	1 (0.4)	0 (0)
COUGH SUPPRESSANTS, EXCL. COMBINATIONS WITH EXPECTORANTS	4 (0.6)	2 (0.8)	2 (0.5)
Opium alkaloids and derivatives	4 (0.6)	2 (0.8)	2 (0.5)
codeine	1 (0.1)	0 (0)	1 (0.2)
pholcodine	2 (0.3)	1 (0.4)	1 (0.2)
dextromethorphan	1 (0.1)	1 (0.4)	0 (0)
**ANTIHISTAMINES FOR SYSTEMIC USE**	**7 (1)**	**3 (1.1)**	**4 (0.9)**
Substituted alkylamines	2 (0.3)	1 (0.4)	1 (0.2)
chlorphenamine	1 (0.1)	1 (0.4)	0 (0)
chlorphenamine, combinations	1 (0.1)	0 (0)	1 (0.2)
Phenothiazine derivatives	2 (0.3)	2 (0.8)	0 (0)
promethazine	1 (0.1)	1 (0.4)	0 (0)
mequitazine	1 (0.1)	1 (0.4)	0 (0)
Piperazine derivatives	1 (0.1)	0 (0)	1 (0.2)
cetirizine	1 (0.1)	0 (0)	1 (0.2)
Other antihistamines for systemic use	2 (0.3)	0 (0)	2 (0.5)
fexofenadine	2 (0.3)	0 (0)	2 (0.5)
**SENSORY ORGANS**	**9 (1.3)**	**0 (0)**	**9 (2)**
**OPHTHALMOLOGICALS**	**9 (1.3)**	**0 (0)**	**9 (2)**
ANTIINFLAMMATORY AGENTS	1 (0.1)	0 (0)	1 (0.2)
Corticosteroids, plain	1 (0.1)	0 (0)	1 (0.2)
dexamethasone	5 (0.7)	2 (0.8)	3 (0.7)
ANTIGLAUCOMA PREPARATIONS AND MIOTICS	7 (1)	0 (0)	7 (1.6)
Carbonic anhydrase inhibitors	3 (0.4)	0 (0)	3 (0.7)

acetazolamide	1 (0.1)	0 (0)	1 (0.2)
dorzolamide	2 (0.3)	0 (0)	2 (0.5)
Beta blocking agents	2 (0.3)	0 (0)	2 (0.5)
levobunolol	1 (0.1)	0 (0)	1 (0.2)
timolol, combinations	1 (0.1)	0 (0)	1 (0.2)
Prostaglandin analogues	2 (0.3)	0 (0)	2 (0.5)
latanoprost	1 (0.1)	0 (0)	1 (0.2)
travoprost	1 (0.1)	0 (0)	1 (0.2)
OCULAR VASCULAR DISORDER AGENTS	1 (0.1)	0 (0)	1 (0.2)
Antineovascularisation agents	1 (0.1)	0 (0)	1 (0.2)
ranibizumab	1 (0.1)	0 (0)	1 (0.2)
**VARIOUS**	**41 (5.7)**	**14 (5.3)**	**26 (5.9)**
**CONTRAST MEDIA**	**41 (5.7)**	**14 (5.3)**	**26 (5.9)**
X-RAY CONTRAST MEDIA, IODINATED	39 (5.4)	14 (5.3)	24 (5.4)
Watersoluble, nephrotropic, low osmolar X-ray contrast media	39 (5.4)	14 (5.3)	24 (5.4)
iohexol	6 (0.8)	2 (0.8)	4 (0.9)
iopamidol	1 (0.1)	0 (0)	1 (0.2)
iopromide	3 (0.4)	1 (0.4)	2 (0.5)
ioversol	5 (0.7)	3 (1.1)	2 (0.5)
iodixanol	11 (1.5)	4 (1.5)	7 (1.6)
iomeprol	12 (1.7)	4 (1.5)	7 (1.6)
iobitridol	1 (0.1)	0 (0)	1 (0.2)
X-RAY CONTRAST MEDIA, NON-IODINATED	1 (0.1)	0 (0)	1 (0.2)
Barium sulfate containing X-ray contrast media	1 (0.1)	0 (0)	1 (0.2)
MAGNETIC RESONANCE IMAGING CONTRAST MEDIA	1 (0.1)	0 (0)	1 (0.2)
Paramagnetic contrast media	1 (0.1)	0 (0)	c

Results as counts and percentage of total number of all reported drugs in the corresponding group. The following substances, reported in one case report each, were not included due to missing Anatomical Therapeutic Chemical Classification System (ATC) code: acetyl choline chloride, loxoprofen, carperitide, prophylline, curcumin, Neurotropin, probiotics, traditional Chinese medicine, and dai-kenchu-to herbal medicine. *Note that hydroxychloroquine has been classified as antimalarial following the ATC system, however, it was indicated for lupus, rheumatoid arthritis, other rheumatic and immune-mediating diseases, and COVID-19.
